# Supplementary material for: Parents’ experiences with a sick or injured child during the COVID-19 lockdown: an online survey in the Netherlands
Source: BMJ Open. 2021 Dec 2;11(12):e055811. doi: 10.1136/bmjopen-2021-055811 (PMC8640193; doi:10.1136/bmjopen-2021-055811)
Supplement: Supplementary data [file bmjopen-2021-055811supp001.pdf]

SUPPLEMENTARY FILE 1

Appendix A: Timeline of measures taken during the first and second lockdown period

**Date**	**Measures**
	First lockdown March 23rd – June 1st 2020
March 23rd	- > 2 persons in public area prohibited with the exception of one household<br>- Group formation only with 1.5 meter distancing rule, maximum 100 persons<br>- Closing of catering industries and schools<br>- Contact professions prohibited<br>- Stores open with 1.5 meter distancing rule and hygiene measures<br>- Households can have maximum of 3 persons visiting them with 1.5 meter distancing rule<br>- Work from home as much as possible<br>- In case of violation 390 euro fine, companies 4350 euro fine
May 11th	- Children day-care and primary schools reopening (in smaller groups)<br>- Contact professions reopening<br>- Playing sports outside allowed with 1.5 meter distance<br>- Opening libraries
June 1st	- Face mask mandatory in public transport, only use public transport when necessary<br>- Restaurants / catering industries reopening for max 30 persons, booking in advance<br>- Museums, cinemas reopening for max 100 persons<br>- High school reopening
June 8th	- Primary school fully reopened
August 6th	- Announcement of the extension of a flight restriction to European countries, the United Kingdom, all countries on the Western hemisphere.
October 14th	- Closing of catering industries

	- A ban on selling alcohol between 8 pm and 7 am<br>- Maximum of 3 visitors per home, maximum of 30 people in interior spaces, outside no more than 4 people from other households.<br>- A ban on late night shopping
December 1st	- Facemasks become mandatory in public places, in schools, in public transport and for contact professions
	Second lockdown December 15th 2020 - February 16th 2021
December 15th	- Closing of non-essential shops<br>- Closing of theatres, cinemas, museums, zoo's, sauna's and libraries<br>- Contact professions closing (not including physiotherapists)<br>- Closing of restaurants and schools (schools starting from December 16th)<br>- Households can have maximal 2 persons visiting them with 1.5 meter distancing rule (on Christmas a maximum of 3 persons)<br>- Work from home as much as possible
December 29th	- Travellers to the Netherlands need to provide a negative PCR-test (not older than 72 hours)
January 6th	- Start of the vaccination program (healthcare workers from the emergency medicine)
January 23rd	- Mandatory curfew from 09 pm until 04.30 am
February 8th	Primary school and day-care reopening
