# Supplementary material for: Parents’ experiences with a sick or injured child during the COVID-19 lockdown: an online survey in the Netherlands
Source: BMJ Open. 2021 Dec 2;11(12):e055811. doi: 10.1136/bmjopen-2021-055811 (PMC8640193; doi:10.1136/bmjopen-2021-055811)
Supplement: Supplementary data [file bmjopen-2021-055811supp002.pdf]

SUPPLEMENTARY FILE 2

Appendix B: Original English version of the online survey

Thank you for helping us with our survey. We would like to find out what impact the Stay Home period is having on parent's actions and decision when caring for, or seeking help for, a sick or injured child. The Stay Home period is sometimes called the lockdown that started on the 23rd March when the government put restrictions on movements to stop the spread of COVID-19. The findings will help us to learn from parents' experiences and direct the development of support for parents with a sick or injured child in the future.

If your child has been ill or injured while you have been following government advice to Stay Home, we would be most grateful if you could complete our survey. We would like to know about your experiences of caring for a sick or injured child at this time, whether or not you decided to seek medical help. We would like parents to complete the survey whether or not their child needed hospital care.

If you do kindly complete and submit the survey this will indicate that you are happy for us to use your responses in our project. Your responses are, of course, anonymous so it will not be possible to identify your replies to remove them after you have completed the survey. Your survey responses will go directly to the survey team.

We will write up the findings from the project and share these in academic journals, in communication with health service leaders and policy makers, at conferences, in education, in future research projects and online. If you provide any free text comments your words may be used but it will not be possible to identify you. If you would like a summary of our findings please contact one of the project leads.

The leads for this project are Professor Sarah Neill sarah.neill@plymouth.ac.uk and Rachel Carter r.carter@plymouth.ac.uk

I can confirm that I have read the information above, I am happy to complete the questionnaire and I am happy for the information I give to be used for the project. Tick box

Are you a UK resident with a child who has been ill or injured since you were asked to Stay Home?

Yes – directed to the questionnaire

No - we thank you for your interest but we are only looking for parents living in the UK whose children have been ill or injured whilst being asked to Stay Home. We hope that you and your family stay well during this period.

It may be that your child has had a number of illnesses or injuries in the last few weeks since you were asked to stay at home. We would like you to select the most serious of these and answer the remaining questions with that in mind.

Question 1 About the child who was ill or injured

a. How old is your child?
   - Under 12 months old
   - 12 months and under 24 months old
   - 2 years and under 5 years old
   - 5 years and under 12 years old
   - 12 years and under 16 years old
   - 16 and under 18 years old

b. Was your child born a girl or boy?
   Girl
   Boy
c. Does your child have any pre-existing illnesses, such as chronic or long term illness, complex needs or a recurring illness?

If yes, please tell us what they are:

Question 2 What signs or symptoms of illness or injury did your child have? Please tick all that apply
Skin and appearance
- Skin very pale or blue, or the inside of their lips and tongue are blue
- Skin appeared mottled
- Rash which disappeared when pressed
- Rash which did not disappear with pressure (the 'Glass test')

Breathing difficulties
- Breathing stopped for a long time (more than 10 seconds at a time),
- Breathing paused (apnoeic episodes) for 5-10 seconds
- Struggling to breathe
- Sucking in muscles under their ribs, between the ribs or at the neck with each breath
- Grunting with the effort of trying to breathe
- Breathing faster than normal
- Noisy breathing (wheeze or a stridor)

Body temperature
- Under 3 months of age with a temperature of over 38ºC/100.4ºF
- 3 and 6 months of age with a temperature over 39ºC/102.2ºF

- Over 6 months old with a temperature above 38ºC/100.4ºF for more than 5 days
- Under 1 month of age and had a low temperature below 36ºC/96.8ºF

Dehydration (seemed to be too dry)
- Dry mouth
- Sunken eyes
- No tears
- Drowsy
- Passing less urine than usual
- Passing more urine than usual
- Drinking more than normal

Pain
- Tummy pain
- Back pain
- Painful testicles
- Headache
- Persistent pain in an injured arm or leg
- Earache
- Sore throat

Change in behaviour
- Agitated
- Persistent crying/inconsolable
- Fit/seizure
- Confused
- Wouldn't wake up or, if woken up, they wouldn't stay awake
- Unresponsive

Injury
- Burn
- Head injury
- Leg or arm injury
- Fall
- Cut(s)
- Bleeding
- Limping
- Difficulty in moving a part of the body

Other, please give details

Question 3 Before the advice to Stay Home, what would your *usual* response have been to this illness or injury? (Please tick one only)

- Care for my child at home
- Ask for medical help
- Something else, please give details

If you would *normally* have asked for medical help please go to Question 4

If you would *not normally* have asked for medical help please go to Question 5

If you answered *something else*, please go to Question 5

Question 4 Where would you *normally* have asked for medical help for your child with this illness or injury <u>in the first instance</u>? (Choose one only)

- NHS Direct 111
- GP surgery
- Via my GP's website
- Video consultation
- Walk in centre
- Urgent care centre
- Minor injuries unit
- GP Out of Hours service
- Calling 999
- Accident and Emergency
- Somewhere else, please say where……

Question 5 Did you ask for medical help for your child during this most recent illness or injury?

Yes, please continue to Question 6

No, please go to Question 9

Question 6 Where did you ask for medical help during this most recent illness or injury? (Please tick all that apply)

- NHS Direct 111
- GP surgery
- Via my GP's website

- Video consultation
- Walk in centre
- Urgent care centre
- Minor injuries unit
- GP Out of Hours service
- Called 999
- Accident and Emergency
- Somewhere else, please say where……

Question 7 Was your child admitted to hospital for this most recent illness or injury? Yes/No

If yes go to question 10

If no, continue to the next question

Question 8 What, if anything else, would you like to tell us about your experience, for example, whether advice was helpful or not helpful.

Go to Question 10 for all respondents

Question 9 Why did you decide not to ask for medical help? Please tick all that apply

- I was not sure if my child was ill or injured enough to need medical help
- I was worried about myself or a member of my family catching COVID19
- I was worried that I might be criticised for using the service if it wasn't an emergency
- I thought the Stay Home advice meant I couldn't go to a health centre or a hospital
- I was worried about using a service when it is needed more urgently by other people
- I was worried that the service would be extremely busy and that I would have to wait for too long
- I don't have a car and didn't want to use public transport
- I don't have anyone to look after my other children
- Something else - please give details

Question 10 What else did you do about your child's illness or injury? Please tick all that apply

a. I waited to see whether or not my child got better
b. I treated my child's illness/injury myself with:
    - paracetamol (Calpol)
    - ibuprofen (Brufen/Junifen)

- a prescribed inhaler
- Vicks or Karvol
- A cold compress/ice pack
- Cleansing
- Bandaging
- A sling to rest an injured arm
- Elevated an injured limb
- Cold drinks
- Warm drinks
- Cool bath
- Warm bath
- Treats and attention
- Distracting my child
- Home remedies, please explain what these were
- Other, please tell us what this was

c. I looked for information on how to manage the illness or injury at home from:
- family
- friends
- a family health professional
- books
- TV
- magazines
- newspapers
- radio
- the internet
- an NHS app
- Other app
- Social media (such as Twitter, Facebook, Instagram, YouTube)
- Google
- NHS Choices
- GP website
- Other website
- Other: please state

d. I used information I already had from (tick all that apply):
- family
- friends
- a family health professional
- books

- TV
- Magazines
- Newspapers
- radio
- an NHS app
- Other app
- Social media (such as Twitter, Facebook, Instagram, YouTube)
- Google
- NHS Choices
- GP website
- Other website
- School or college course
- A mixture of these resources
- Other: please state

Question 11 Helpfulness of information

a. What, if any, information was helpful and if so why?

b. What, if any, information was unhelpful and if so why?

Question 12 Advice provided during the Stay Home period

a. Which of the following sources of advice have you seen about when to use health services while families are being told to Stay Home? Please tick all that apply

- Government sources (such as Government TV or radio broadcast, the letter from Boris Johnson, daily briefings from Government)
- NHS sources (such as NHS Choices website, GP website, 111 service announcements)
- Other expert online sources (such as University, research organisations etc)
- Experts on Social Media sites (such as Twitter, Facebook)
  - Family/Friends on Social Media sites
  - Other, please give details

b. Have you seen advice about when to (Please tick all that apply)
   - call NHS111?
   - call your GP/family doctor?
   - attend accident and emergency with your children?

c. What, if anything, was new about advice on health service use since you have been told to Stay Home?

Question 13 Have the changes to health services during the Stay Home period affected how ill your child has been? Yes, Maybe, No

d. If yes, please tell us how.

Question 14 Have the changes to health services during the Stay Home period affected any treatment your child received? Yes, Maybe, No, Not applicable

e. If yes, please us how.

Please tell us about your family

Question 15 How old are the children in the household?

**Age of children**	**Please tell us the number of children in each age group**
0-4 years	
5-11 years	
12-15 years	
16-18 years	

Question 16 What area do you live in?

- Scotland
- Wales
- Northern Ireland
- East of England
- London
- Midlands
- North East and Yorkshire
- North West
- South East
- South West

Question 17 Which of the following best describes the area in which you live?

- Rural
- Urban

Questions 18 What access do you have to technology: Please tick all that apply

a. What type of mobile phone do you have?
    - for calls and texting only
    - Smartphone
b. What type of mobile phone contract do you have?
    - Pay as you go mobile phone
    - Monthly contract mobile phone
c. What type of computer do you have?
    - laptop
    - desktop computer
d. How much WiFi access do you have?
    - Unlimited WiFi access
    - Limited WiFi access
    - Other access to the internet, please state

Question 19 Do you have access to outdoor space? Yes/No

Question 20 We know that some children are still able to attend school. Where were the school aged children in your family when the illness or injury happened?

- All of my children were attending school
- Some of my children were attending school
- All of my children were staying at home

Question 21 What if, anything else, would you like to tell the project team?

Thank you for taking the time to complete our questionnaire.

If you would like to receive a summary of the project findings please email one of the project leads:

Professor Sarah Neill *sarah.neill@plymouth.ac.uk* or Rachel Carter *r.carter@plymouth.ac.uk*

If you have any concerns or complaints about the project please contact the Research Administrator to the Faculty Research Ethics and Integrity Committee (*hhsethics@plymouth.ac.uk*)
