# Supplementary material for: Parents’ experiences with a sick or injured child during the COVID-19 lockdown: an online survey in the Netherlands
Source: BMJ Open. 2021 Dec 2;11(12):e055811. doi: 10.1136/bmjopen-2021-055811 (PMC8640193; doi:10.1136/bmjopen-2021-055811)
Supplement: Supplementary data [file bmjopen-2021-055811supp003.pdf]

SUPPLEMENTARY FILE 3

Appendix C: Adapted Dutch version of the online survey

Bedankt voor uw hulp bij onze enquête. Wij willen graag weten wat voor gevolgen de "lockdown periode" heeft gehad op de acties en beslissingen van ouders bij de zorg of het zoeken van hulp voor een ziek of gewond kind. Onze "intelligente lockdown" ging in op 23 maart 2020 toen de overheid beperkingen oplegde om zo de verspreiding van COVID-19 te stoppen. De resultaten zullen ons helpen om te leren van de ervaringen van ouders en de ondersteuning voor ouders met een ziek of gewond kind in de toekomst te verbeteren.

Indien uw kind ziek of gewond was terwijl u het advies van de overheid opvolgde om zoveel mogelijk thuis te blijven gedurende de intelligente lockdown periode van 23 maart tot 1 juni 2020, zouden we u zeer dankbaar zijn als u onze enquête wilt invullen. We willen weten wat uw ervaringen zijn met de zorg voor een ziek of gewond kind gedurende deze periode en of u besloot om wel of niet medische hulp te zoeken. We willen graag weten of uw kind een vorm van gezondheidszorg (zoals het raadplegen van de huisarts, de huisartsenpost of ziekenhuiszorg) nodig had.

Indien u de enquête volledig invult en indient, geeft u toestemming aan ons om uw antwoorden voor ons onderzoek te gebruiken. Uw antwoorden zijn volledig anoniem en niet naar u of uw kind te herleiden. Uw antwoorden gaan rechtstreeks naar het onderzoeksteam.

De enquête bestaat uit ongeveer 20 vragen en het invullen hiervan zal maximaal 10 minuten duren.

We zullen de resultaten van het onderzoek delen in wetenschappelijke tijdschriften en websites, in communicatie met artsen en beleidsmakers, op congressen, in onderwijs en in toekomstige onderzoeksprojecten. Als u opmerkingen in de vrije tekst invult, kunnen uw woorden worden gebruikt, maar is het niet mogelijk u te identificeren. Indien u een samenvatting van onze resultaten wilt, neem dan contact op met een van de projectleiders.

Dit project is een samenwerking tussen het Erasmus MC Sophia Kinderziekenhuis in Nederland en University of Plymouth en University College London in Engeland. Deze studie is goedgekeurd door de Commissie Wetenschappelijk Onderzoek COVID-19 van het Erasmus Medisch Centrum te Rotterdam (CWO COVID-19 EMC).

De projectleiders zijn professor Henriette Moll, kinderarts Dorine Borensztajn en arts-onderzoeker Chantal Tan.

Indien u nog vragen heeft over het onderzoek, kunt u contact opnemen met de projectleiders door een mail te sturen naar *ervaringen.ouders@erasmusmc.nl*.

Introductie vragen over deelname aan enquête en manier van werving

1) Ik kan bevestigen dat ik bovenstaande informatie heb gelezen en ik wil graag de enquête invullen en informatie geven voor het onderzoek.

- Ja
- Nee

2) Bent u een inwoner van Nederland en was uw kind ziek of gewond kind terwijl u het advies van de overheid opvolgde om zoveel mogelijk thuis te blijven gedurende de lockdown periode?

- Ja – u wordt doorverwezen naar de vragenlijst
- Nee – we danken u voor uw interesse, maar we zijn alleen op zoek naar ouders die in Nederland wonen en wiens kind(eren) ziek of gewond was tijdens de lockdown periode.

3). Hoe bent u bij deze vragenlijst terecht gekomen?

- Ik zag een bericht met de link naar dit formulier in een Facebook groep voor ouders/moeders
- Ik zag een bericht met de link naar dit formulier via-via op Facebook
- Ik zag een bericht met de link naar dit formulier op LinkedIn
- Ik zag een bericht met de link naar dit formulier op Twitter
- Ik kreeg een bericht met de link naar dit formulier via WhatsApp toegestuurd

1. Vragen over het zieke of gewonde kind

Het kan zijn dat uw kind meerdere ziekte episodes of verwondingen heeft gehad tijdens de “lockdown periode”. We willen graag dat u de meest ernstige hiervan selecteert en de volgende vragen met die ziekte of verwonding in gedachte beantwoordt.

a). Hoe oud was uw kind ten tijde van de ziekte of verwonding?

- Jonger dan 12 maanden oud
- 12 maanden of ouders maar jonger dan 24 maanden oud
- 2 jaar of ouders maar jonger dan 5 jaar oud
- 5 jaar of ouder maar jonger dan 12 jaar oud
- 12 jaar of ouders maar jonger dan 16 jaar oud
- 16 of 17 jaar oud

b). Is uw kind een jongen of een meisje?

- Meisje
- Jongen

c). Is uw kind bekend met reeds bestaande ziekten, zoals chronische of langdurige ziekten, complexe behoeften of terugkerende ziekten?
Indien ja, graag toelichting welke ziekte(n) dit zijn:

2. Welke kenmerken of symptomen van ziekte of verwonding had uw kind.
Vink alles aan wat van toepassing is

- Huid en uiterlijk
  - Huid erg bleek of blauw, of binnenkant van de lippen en tong blauw
  - Vlekkerige huid
  - Uitslag die verdween wanneer erop werd gedrukt
  - Uitslag die niet verdween wanneer erop werd gedrukt
- Ademhalingsproblemen
  - Ademhaling stopte voor een lange periode (>10 seconden per keer)
  - Ademhaling onderbroken (ademstops) voor 5-10 seconden
  - Moeite hebben met ademen
  - Intrekkingen in de hals, en/of onder of tussen de ribben bij elke ademhaling
  - Kreunen bij de ademhaling
  - Sneller ademen dan normaal
  - Hoorbare ademhaling (gierende of piepende ademhaling)
- Lichaamstemperatuur
  - Jonger dan 3 maanden oud met een temperatuur boven de 38oC
  - Tussen de 3 en 6 maanden oud met een temperatuur boven de 39oC
  - Ouder dan 6 maanden oud met een temperatuur boven de 38oC voor langer dan 5 dagen
  - Jonger dan 1 maand oud met een temperatuur onder de 36oC
- Uitdroging
  - Droge mond
  - Ingevallen ogen
  - Geen tranen
  - Suf
  - Minder plassen dan normaal
  - Meer plassen dan normaal
  - Minder drinken dan normaal
  - Meer drinken dan normaal
- Pijn
  - Buikpijn
  - Rugpijn
  - Pijnlijke testikels
  - Hoofdpijn
  - Persisterende pijn in een gewond arm of been
  - Oorpijn
  - Keelpijn

- Verandering in gedrag
  - Geagiteerd (sneller boos of geïrriteerd zijn)
  - Aanhoudend huilen/ontroostbaar
  - Toeval of epileptische aanval
  - Verward
  - Niet wakker worden of niet wakker kunnen blijven
  - Niet reagerend
- Verwonding
  - Verbranding
  - Hoofdverwonding
  - Verwonding aan been of arm
  - Val
  - Snijwond
  - Bloeding
  - Mank lopen
  - Moeite om een lichaamsdeel te bewegen
- Andere ziekte of verwonding

Mocht de ziekte en/of verwonding van uw kind niet hierboven vermeld staan, graag hieronder uw toelichting

3. Wat zou uw gebruikelijke reactie zijn op deze ziekte of verwonding zijn geweest vóór de intelligente lockdown periode?

- Om medische hulp vragen → ga naar vraag 4
- Thuis voor mijn kind zorgen → ga naar vraag 5
- Anders → ga naar vraag 5

4. Waar zou u normaliter allereerst om medische hulp hebben gevraagd voor uw kind bij deze ziekte of verwonding?

- Eigen waarnemend huisarts (overdag)
- Telefonisch consult met de huisarts
- Inloop spreekuur bij de huisarts
- Website eigen huisarts
- Website thuisarts.nl
- Huisartsenpost (in de avond/nacht)
- Behandelend kinderarts, waar mijn kind onder controle is
- 112 bellen
- Spoedeisende hulp
- Anders

5. Heeft u om medische hulp gevraagd voor de ziekte of verwonding van uw kind tijdens de lockdown?

- Ja → ga naar vraag 6
- Nee → ga naar vraag 9

6. Waar heeft u om medische hulp gevraagd voor deze ziekte of verwonding tijdens de lockdown?

Vink alles aan wat van toepassing is

- Eigen waarnemend huisarts (overdag)
- Telefonisch consult met de huisarts
- Inloop spreekuur bij de huisarts
- Website eigen huisarts
- Website thuisarts.nl
- Huisartsenpost (in de avond/nacht)
- Behandelend kinderarts, waar mijn kind onder controle is
- 112 bellen
- Spoedeisende hulp
- Anders

7. Was uw kind opgenomen in het ziekenhuis vanwege de ziekte of verwonding tijdens de lockdown?

8. Wat zou u nog willen vertellen over uw ervaring? Bijvoorbeeld of het advies wat u heeft gekregen nuttig was of niet. → ga naar vraag 10

9. Waarom had u besloten om geen medische hulp te vragen? Vink alles aan wat van toepassing is

- Ik wist niet zeker of mijn kind ziek of gewond genoeg was om medische hulp te zoeken
- Ik was bezorgd dat ikzelf, mijn kind of een familielid COVID-19 zou krijgen
- Ik was bezorgd dat ik kritiek zou krijgen als ik medische zorg zou gebruiken als het geen noodgeval was
- Ik dacht dat het advies om zoveel mogelijk thuis te blijven tijdens de lockdown periode betekende dat ik niet naar een gezondheidsinstelling of ziekenhuis kon gaan
- Ik maakte me zorgen over het gebruik van medische zorg wanneer deze voor andere mensen meer nodig was
- Ik was bang dat de gezondheidsinstelling (huisarts of ziekenhuis) extreem druk zou zijn en dat ik lang zou moeten wachten
- Ik heb geen auto en ik wilde geen gebruik maken van het openbaar vervoer
- Ik heb niemand die op mijn andere kinderen kan passen

- Anders

10. Wat heeft u nog meer gedaan aan de ziekte of verwonding van uw kind?

- Ik heb gewacht om aan te kijken of mijn kind al dan niet beter werd
- Ik heb de ziekte of verwonding van mijn kind zelf behandeld. Vink alles aan wat van toepassing is
    - Paracetamol
    - Ibuprofen
    - Een voorgeschreven puffer
    - Vicks
    - Een koude kompres/koeling met ijs
    - Schoonmaken van de wond
    - Verbinden
    - Een draagdoek om een gewond arm te laten rusten
    - Een gewond ledemaat ondersteunen door het hoog te houden
    - Koude dranken
    - Warme dranken
    - Koud bad
    - Warm bad
    - Traktaties en aandacht
    - Mijn kind afleiden
    - Huismiddeltjes, graag uw toelichting
    - Anders
- Ik heb informatie gezocht over hoe ik thuis de ziekte of verwonding kon behandelen**.** Kunt u toelichten waar of via wie u deze informatie heeft opgezocht?
    - Familie
    - Vrienden
    - Een vriend of familielid die zorgmedewerker is
    - Boeken
    - TV
    - Tijdschriften
    - Kranten
    - Radio
    - Het internet
    - App: moet ik naar de dokter?
    - Andere apps
    - Sociale media (zoals Twitter, Facebook, Instagram, Youtube)
    - Google
    - Thuisarts.nl
    - Een andere website

  - Anders
- Ik heb informatie gebruikt welke ik al had. Kunt u toelichten waarvan of van wie u deze informatie al had gekregen?
  - Familie
  - Vrienden
  - Een vriend of familielid die zorgmedewerker is
  - Boeken
  - TV
  - Tijdschriften
  - Kranten
  - Radio
  - Het internet
  - App: moet ik naar de dokter?
  - Andere apps
  - Sociale media (zoals Twitter, Facebook, Instagram, Youtube)
  - Google
  - Thuisarts.nl
  - Een andere website
  - Anders
  -
- Niet van toepassing

11. Als de gevonden informatie nuttig was om u te helpen met de ziekte of verwonding van uw kind, wat vond u hier nuttig aan en waarom?
Als de gevonden informatie niet nuttig was, wat vond u hier niet nuttig aan en waarom?

12. Welke van de volgende bronnen voor advies bent u tegen gekomen wanneer u medische zorg nodig had gedurende de lockdown. Vink alles aan wat van toepassing is.

- Overheidsbronnen (dagelijks nieuws van overheid/RIVM)
- Gezondheidszorg bronnen (zoals website van uw eigen huisarts, thuisarts.nl, app moet ik naar de dokter?)
- Andere online expert bronnen (zoals universitaire of onderzoeksorganisaties etc)
- Deskundigen op sociale media (zoals Twitter, Facebook)
- Familie/vrienden op sociale media
- Anders

Heeft u advies gevonden in deze bron(nen) over wanneer u…

- ... de huisartsenpost moest bellen?
- … uw eigen huisarts moet bellen?

- … naar de spoedeisende hulp moet komen met uw kind?

Wat was nieuw aan het advies wat u heeft gezien omtrent het gebruik van medische zorg gedurende de lockdown?

13. Hebben veranderingen in de medische zorg gedurende de lockdown invloed gehad op hoe ziek uw kind was?

- Ja → graag uw toelichting
- Nee

14. Hebben veranderingen in medische zorg gedurende de lockdown invloed gehad op de behandeling die uw kind heeft gekregen?

- Ja → graag uw toelichting
- Nee
- Misschien
- Niet van toepassing

15. Hoe oud waren de kinderen in uw huishouden gedurende de lockdown periode? Vul het aantal kinderen per leeftijdsgroep in

- Leeftijd van de kinderen 0-4 jaar
- Leeftijd van de kinderen 5-11 jaar
- Leeftijd van de kinderen 12-15 jaar
- Leeftijd van de kinderen 16-17 jaar

16. In welke provincie woont u?

- Drenthe
- Flevoland
- Friesland
- Gelderland
- Groningen
- Limburg
- Noord-Brabant
- Noord-Holland
- Overijssel
- Utrecht
- Zeeland
- Zuid-Holland

Welke van de volgende opties omschrijft uw woonomgeving het beste?

- Grote stad
- Kleine stad/dorp
- Platteland

Had u de mogelijkheid om tijdens de lockdown buiten in uw tuin of op uw balkon te zijn?

- Ja
- Nee

17. Welke toegang heeft u tot technologie?

Wat voor mobiele telefoon heeft u ?

- Prepaid mobiele telefoon
- Maand abonnement
- Niet van toepassing

Wat voor soort computer heeft u?

- Een laptop
- Computer
- Ik heb geen computer

In welke mate heeft u toegang tot WiFi?

- Onbeperkte WiFi toegang
- Beperkte WiFi toegang
- Geen WiFi toegang
- Anders

18. We weten dan sommige kinderen nog naar school konden gaan. Waar waren de schoolgaande kinderen in uw gezin wanneer ze ziek of gewond waren gedurende de lockdown?

- Al mijn kinderen gingen naar school
- Sommige van mijn kinderen gingen naar school
- Al mijn kinderen bleven thuis

19. Heeft u nog opmerkingen voor het onderzoeksteam?

Hartelijk dank voor uw tijd en interesse om deze vragenlijst te beantwoorden.

Indien u een samenvatting van de bevindingen van het onderzoek zou willen ontvangen, stuur dan een mail naar de projectleider: Professor Henriette Moll (*ervaringen.ouders@erasmusmc.nl*)

Indien u enige zorgen of klachten heeft over het onderzoek, neem dan graag contact op met een onafhankelijk kinderarts (*secretariaatalkg@erasmusmc.nl*)
