# Supplementary material for: Parents’ experiences with a sick or injured child during the COVID-19 lockdown: an online survey in the Netherlands
Source: BMJ Open. 2021 Dec 2;11(12):e055811. doi: 10.1136/bmjopen-2021-055811 (PMC8640193; doi:10.1136/bmjopen-2021-055811)
Supplement: Supplementary data [file bmjopen-2021-055811supp004.pdf]

SUPPLEMENTARY FILE 4

Appendix D: Introduction of the survey on social media

Advertising text:

De coronaperiode is een zware dobber voor ouders. Is uw kind ziek of gewond geweest in de lockdown periode en wilt u bijdragen aan onderzoek van het Sophia Kinderziekenhuis, vul dan deze online enquête anoniem in (maximale duur 10 minuten):

h*ttps://forms.gle/GNyiP7G2xKnoSR9j8*

Alvast bedankt voor uw medewerking. Delen van dit bericht wordt zeer gewaardeerd!

Translation of advertising text:

The corona period is a tough one for parents. If your child has been ill or injured during the lockdown period and you want to contribute to research of the Sophia Children's Hospital, please complete this online survey anonymously (maximum duration 10 minutes):

https://forms.gle /GNyiP7G2xKnoSR9j8

Thank you in advance for your cooperation. Sharing this post is much appreciated!

Advertising image:

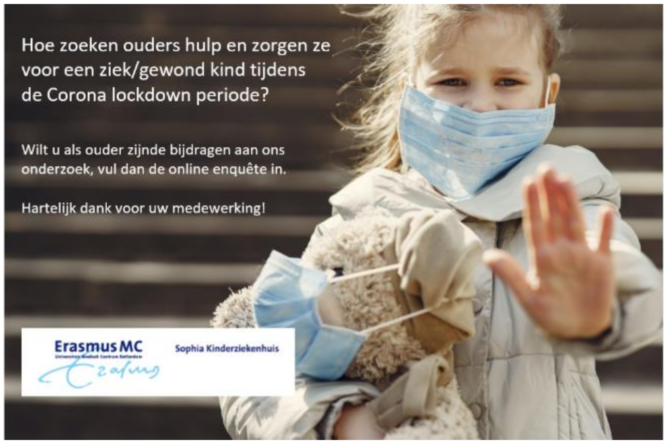

*The image was obtained from the following webpage and was freely available to use: https://www.pexels.com/photo/serious-girl-in-protective-mask-holding-plush-toy-in-mask-and-showing-palm-against-steps-4000605/

Translation of text on advertising image:

How do parents seek help and care for a sick or injured child during the corona lockdown period? Do you as a parent want to contribute to research, please fill in this online survey. Thank you very much for your cooperation!
