# Supplementary material for: Parents’ experiences with a sick or injured child during the COVID-19 lockdown: an online survey in the Netherlands
Source: BMJ Open. 2021 Dec 2;11(12):e055811. doi: 10.1136/bmjopen-2021-055811 (PMC8640193; doi:10.1136/bmjopen-2021-055811)
Supplement: Supplementary data [file bmjopen-2021-055811supp005.pdf]

SUPPLEMENTARY FILE 5

Appendix E: Table for McNemar's test

		Did seek help during lockdown		
**Would have sought help before lockdown**		*Yes*	*No*	*Total children*
	Yes	*83*	*4*	*87*
	No	*9*	*9*	*18*
	Total children	*92*	*13*	*105*

McNemar's test p=0.2
