# Supplementary material for: Parents’ experiences with a sick or injured child during the COVID-19 lockdown: an online survey in the Netherlands
Source: BMJ Open. 2021 Dec 2;11(12):e055811. doi: 10.1136/bmjopen-2021-055811 (PMC8640193; doi:10.1136/bmjopen-2021-055811)
Supplement: Supplementary data [file bmjopen-2021-055811supp006.pdf]

SUPPLEMENTARY FILE 6

Appendix F: Parents' help seeking behaviour related to children's characteristics

	**Sick/injured children (N=105)**	**Would have sought help before lockdown ¥**	**Did seek help during lockdown ¥**
**Age** (years)			
<5	41 (39)	37 (90)	39 (95)
5-11	43 (41)	33 (76)	36 (84)
12-17	21(20)	17(81)	17 (81)
**Gender**			
Boys	61 (58)	49 (80)	52 (85)
Girls	44 (42)	38 (86)	40 (91)
**Presenting symptom***			
Skin	22 (21)	18 (82)	21 (95)
Breathing	25 (24)	21 (84)	24 (96)
Body temperature	16 (15)	16 (100)	16 (100)
Dehydration	21 (20)	18 (86)	20 (95)
Pain	45 (45)	40 (89)	44 (94)
Change of behaviour	42 (40)	32 (76)	38 (91)
Injury	23 (22)	19(83)	23 (100)
Other	19 (18)	17 (89)	15 (75)
**Hospital admission**	37 (35)	36 (97)	37 (100)

Absolute numbers and percentages (%) are shown

* Possible to have more than 1 presenting symptom

¥ Percentage of children in that specific variable group
