# Supplementary material for: Parents’ experiences with a sick or injured child during the COVID-19 lockdown: an online survey in the Netherlands
Source: BMJ Open. 2021 Dec 2;11(12):e055811. doi: 10.1136/bmjopen-2021-055811 (PMC8640193; doi:10.1136/bmjopen-2021-055811)
Supplement: Supplementary data [file bmjopen-2021-055811supp007.pdf]

SUPPLEMENTARY FILE 7

Appendix G: Selection of quotes from parents

Severity of illness – quotes based on 32 open text answers

Positive experiences	Negative experiences
**Health care services**	
"Normally the waiting time at the emergency department can take hours. However, now we were seen and treated within 30 minutes. This spared my child hours of pain !!! Thus a very positive experience!!!!"	"My son had to undergo surgery in a tertiary hospital. Normal waiting time would be 2 weeks, but now it was extended to 1.5 month. During these times, he cried a lot and was in a lot of pain; pure hell."
"She underwent surgery on her heart defect during the lockdown."	"The doctor couldn't see my son, so the severity of the wound wasn't clear. The advice "to call back in a few days" wasn't of much help…"
	"Because my child was not seen at the GP out of office for his double ear infection, he received his antibiotic treatment later."
**Parents' behaviour**	
	"We waited a day / night too long to seek medical help out of fear."
**Reported health care professionals' behaviour**	
"The emergency department was empty and we were nearly the only ones at the paediatric ward. The doctors had a lot of time for us."	"They wanted the patients to leave as soon as possible."

Comparable quotes are not separately described

Treatment – quotes based on 39 open text answers

Positive experiences	Negative experiences
**Health care services**	
" All elective surgeries were postponed and the paediatric ward has never been this empty. We received the best medical care during this period. There was enough time to properly start palliative care."	"My son needed to wait much longer on his surgery than if there had not have been a lockdown."
	"My child did not undergo surgery but received a higher dose of antibiotics."
**Parents' behaviour**	
	"We waited longer to consult the GP. Normally we would have contacted the GP earlier, but we did not out of fear of getting infected with COVID-19 and the impact of the protective clothing health care workers wore on our child."

BMJ Publishing Group Limited (BMJ) disclaims all liability and responsibility arising from any reliance placed on this supplemental material which has been supplied by the author(s)

**Reported health care professionals' behaviour**	
	" We have spent hours in the emergency department in an isolated room without any communication. The nurses gave us little attention and we were asked to take care of the nightshift ourselves because it was difficult for the nurses to change into protective clothing. Reason for us to go home the next day."
	"The doctor could not see us for a consultation. I felt unheard and not understood."
	"Our GP was very worried, leading to daily consultations while it all started with a simple fever."

Comparable quotes are not separately described
