# Supplementary material for: Parents’ experiences with a sick or injured child during the COVID-19 lockdown: an online survey in the Netherlands
Source: BMJ Open. 2021 Dec 2;11(12):e055811. doi: 10.1136/bmjopen-2021-055811 (PMC8640193; doi:10.1136/bmjopen-2021-055811)
Supplement: Author's manuscript [file bmjopen-2021-055811.draft_revisions.pdf]

BMJ Open

BMJ Open is committed to open peer review. As part of this commitment we make the peer review history of every article we publish publicly available.

When an article is published we post the peer reviewers' comments and the authors' responses online. We also post the versions of the paper that were used during peer review. These are the versions that the peer review comments apply to.

The versions of the paper that follow are the versions that were submitted during the peer review process. They are not the versions of record or the final published versions. They should not be cited or distributed as the published version of this manuscript.

BMJ Open is an open access journal and the full, final, typeset and author-corrected version of record of the manuscript is available on our site with no access controls, subscription charges or pay-per-view fees (http://bmjopen.bmj.com).

If you have any questions on BMJ Open's open peer review process please email info.bmjopen@bmj.com

BMJ Open

PARENTS' EXPERIENCES WITH A SICK OR INJURED CHILD DURING THE COVID-19 LOCKDOWN: AN ONLINE SURVEY IN THE NETHERLANDS

Journal:	*BMJ Open*
Manuscript ID	bmjopen-2021-055811
Article Type:	Original research
Date Submitted by the Author:	26-Jul-2021

Complete List of Authors:	Tan, Chantal; Erasmus MC Sophia Children Hospital, General Paediatrics<br>Lutgert, Eveline; Erasmus MC Sophia Children Hospital, General Paediatrics<br>Neill, Sarah; University of Plymouth, Faculty of Health<br>Carter, Rachel; University of Plymouth, Faculty of Health<br>Jones, Ray; University of Plymouth, Faculty of Health, Education, and Society<br>Chynoweth, Jade; University of Plymouth, Faculty of Health<br>Borensztajn, Dorine; Erasmus MC Sophia Children Hospital, General Paediatrics<br>Lakhanpaul, Monica; University College London, Institute of Child Health<br>moll, Henriette; Erasmus MC Sophia Children Hospital, Department of Pediatrics

Keywords:	COVID-19, PAEDIATRICS, Community child health < PAEDIATRICS

SCHOLARONE™
Manuscripts

I, the Submitting Author has the right to grant and does grant on behalf of all authors of the Work (as defined in the below author licence), an exclusive licence and/or a non-exclusive licence for contributions from authors who are: i) UK Crown employees; ii) where BMJ has agreed a CC-BY licence shall apply, and/or iii) in accordance with the terms applicable for US Federal Government officers or employees acting as part of their official duties; on a worldwide, perpetual, irrevocable, royalty-free basis to BMJ Publishing Group Ltd ("BMJ") its licensees and where the relevant Journal is co-owned by BMJ to the co-owners of the Journal, to publish the Work in this journal and any other BMJ products and to exploit all rights, as set out in our licence.

The Submitting Author accepts and understands that any supply made under these terms is made by BMJ to the Submitting Author unless you are acting as an employee on behalf of your employer or a postgraduate student of an affiliated institution which is paying any applicable article publishing charge ("APC") for Open Access articles. Where the Submitting Author wishes to make the Work available on an Open Access basis (and intends to pay the relevant APC), the terms of reuse of such Open Access shall be governed by a Creative Commons licence – details of these licences and which Creative Commons licence will apply to this Work are set out in our licence referred to above.

Other than as permitted in any relevant BMJ Author's Self Archiving Policies, I confirm this Work has not been accepted for publication elsewhere, is not being considered for publication elsewhere and does not duplicate material already published. I confirm all authors consent to publication of this Work and authorise the granting of this licence.

For peer review only

PARENTS' EXPERIENCES WITH A SICK OR INJURED CHILD DURING THE COVID-19 LOCKDOWN: AN ONLINE SURVEY IN THE NETHERLANDS

Chantal D. Tan[1], Eveline K. Lutgert[1], Sarah Neill[2], Rachel Carter[2], Ray Jones[2], Jade Chynoweth[2], Dorine M. Borensztajn[1], Monica Lakhanpaul[3], Henriette A. Moll[1]

Author affiliations:

1. Erasmus MC-Sophia Children's Hospital, Department of General Paediatrics, Rotterdam, the Netherlands
2. Faculty of Health, University of Plymouth, United Kingdom
3. Integrated Community Child Health Population, Policy & Practice Department, UCL Great Ormond Street Institute of Child Health, University College London, United Kingdom

Corresponding author:

Chantal D. Tan, MD

Department of General Paediatrics, Erasmus MC – Sophia Children's Hospital

P.O. Box 2060, 3000 CB, Rotterdam, the Netherlands

Telephone: +31 10 703 72 39

E-mail: c.tan@erasmusmc.nl

Word count: 3546 words

For peer review only

Abstract

Objective: To assess the impact of the COVID-19 lockdown on parents' help seeking behaviour and care for a sick or injured child in the Netherlands.

Design and setting: An online survey on parents' experiences with a sick or injured child during the COVID-19 lockdown periods was disseminated through social media.

Participants: Parents living in the Netherlands with a sick or injured child during the lockdown periods from March until June 2020 and from December 2020 until February 2021 were eligible to participate.

Outcome measures: Descriptive statistics and thematic analysis were used to analyse family and children's characteristics, parents' response to a sick or injured child, and the perceived impact of the lockdown on child's severity of illness and treatment reported by parents. Analyses were stratified for children with and without chronic conditions.

Results: Of the 105 parents who completed the survey, 83% reported they would have sought medical help before lockdown compared to 88% who did seek help during lockdown. Parents reported that changes in health services affected their child's severity of illness (31%) and their treatment (39%), especially for children with chronic conditions. Negative experiences included long waiting lists, delayed referral and more consultations by telephone. During lockdown, there was no change in help seeking behaviour by parents of children with a chronic condition (N=51) compared to parents of children without a chronic condition.

Conclusion: Parents in the Netherlands who completed the survey were not deterred from seeking medical help for their sick or injured child during the COVID-19 lockdown periods. However, changes in health services affected child's severity of illness and treatment, especially for children with chronic conditions.

For peer review only

Article summary

Strengths and limitations of this study

• Online surveys enable the collection of anonymised data and facilitate the collection of data from a wide range of patients regardless of their residency.

• The survey was disseminated through social media to reach the general population.

• The survey had several 'other' options where parents could give a more detailed explanation of their answers in addition to the multiple-choice answers.

• The advertisement through social media could have caused selection bias as parents who do not have social media or parents with limited (digital) literacy could not have filled in the survey.

• Our study population might not be a good reflection of the general population of ill or injured children as almost 50% of the children had a chronic condition.

For peer review only

Background

Globally, health care professionals reported that the number of paediatric patients visiting medical services declined significantly while lockdowns were in effect due to the Coronavirus disease 2019 (COVID-19) pandemic. The estimated decline of paediatric visits to Emergency Departments (EDs) during lockdown ranged from 30% in the United Kingdom to 89% in Italy.(1-4) In the Netherlands all medical visits to Dutch EDs declined by 25% during the first lockdown from March 23rd until June 1st 2020.(5) The Dutch Healthcare Authority has reported that paediatric urgent referrals by general practitioners (GPs) declined more than referrals by GPs to other specialties.(6) This decline could partially be explained by a lower incidence of illness or injury in children during lockdown as limited contact with others reduced exposure to infectious diseases and restricted activities reduced accidental injuries.(7) However, other specialties have shown a recuperation of the number of referrals after the first lockdown, while the number of paediatric referrals has not.(6) The Netherlands had two lockdown periods where civilians were strongly advised to mainly stay at home and schools were closed.

Other explanations for the decline in paediatric medical visits are parental concern about COVID-19 and barriers raised by healthcare professionals.(8) On one hand, there may be positive outcomes from parents' self-care of their children as they develop ways of coping with and managing their child's illness or injury themselves. On the other hand, it is also important to notice the possible negative outcomes of the decrease in parents seeking help for a sick or injured child. In a survey set out by the Dutch Paediatric Society amongst 1400 Dutch paediatricians, there were 51 cases reporting unnecessary collateral harm as a result of delayed presentation to medical health services due to the COVID-19 pandemic. These reports include several cases of diabetic ketoacidosis, sepsis, and cancer.(9) Similar results were found in surveys set out in other European countries.(8, 10, 11) Unfortunately, these surveys do not differentiate between delay caused by parents or by health care professionals.

It is imperative that we try to unravel the reasoning behind the help seeking behaviour of parents for ill or injured children and the impact that the COVID-19 lockdown had on their behaviour. Special attention should be given to parents of children with chronic conditions. They may seek less medical care as they are afraid of COVID-19 infection for their vulnerable child. Concerns were also raised regarding the mental health of children and adolescents during these difficult times. Based on a survey in the United States, 35% of

For peer review only

adolescents receive mental health support solely in an educational setting.(12) With schools closing, it is possible that certain services couldn't be accessed properly.(13) Children also ended up spending more time at home and were socially isolated, which may have negatively affected their mental health.(14, 15)

We aimed to assess the impact of the COVID-19 lockdown on parents' experiences with a sick or injured child in the Netherlands, stratified for children with and without chronic condition. In more detail, we focus on whether and where parents sought help and whether and why their help seeking behaviour had changed due to the lockdown period. In addition, we assessed parents' perceptions of the impact of the lockdown on their child's severity of illness and treatment.

Methods

Study design

This study was a COVID-19 related project in collaboration with the University College London and the University of Plymouth. It was an observational study consisting of an online survey assessing parents' help seeking behaviour for, and care of, a sick or injured child during two COVID-19 lockdown periods. Approval by the ethical committee of the Erasmus MC Sophia was obtained. Informed consent of the participating parents was obtained.

Study population and setting

Parents living in the Netherlands with a sick or injured child during the lockdown periods from March 23rd until June 1st 2020 and from December 15th 2020 until February 16th 2021 were included. The main measures taken during these lockdown periods are shown in chronological order in Appendix A. *(Supplementary file 1)* We excluded parents who did not live in the Netherlands or who did not have a sick or injured child during the afore mentioned lockdown periods. Our aim was to recruit about 100 respondents to our survey. For a sample size of 100, the 95% confidence interval for a 50% estimate of proportions is 40.2% to 59.8%.(16, 17)

Data collection

The original English questionnaire was translated to the Dutch language and some answer options were adapted to the Dutch health care system. *(Supplementary file 2 Appendix B, Supplementary file 3 Appendix C)*. The questionnaire was launched as an online survey

For peer review only

created in Google Forms, which enabled the collection of anonymised data. Online surveys facilitate the collection of data from a wide range of parents regardless of their residency. The survey was mainly composed of multiple-choice questions, but 'other' options to add free text were available. This 'other' option also enabled parents to give a more detailed explanation on their previous chosen multiple-choice answer. Respondents were recruited through social media and snowballing. Information about the survey was posted by the research group on Facebook, Twitter, LinkedIn, Instagram, WhatsApp and professional contacts were sent a request to share the information about the study with their social contacts. Facebook was divided into personal Facebook accounts and Facebook groups, of which the latter included private Facebook groups for parents as well as public Facebook pages of the Erasmus MC and the Sophia Children's Hospital. A short introduction to the survey and a link to the survey was written to share on social media *(Supplementary file 4 Appendix D).* A reminder was posted on social media every two weeks. Key persons who had shared the survey on their social media and their estimated reach were recorded in a document to keep track of the spread of the survey and the number of respondents.

Patient and public involvement

The public was involved in the design and conduct of the study. Feedback on the questionnaire was given by paediatricians and PhD students of the research group of General Paediatrics Erasmus MC Sophia. Feedback of parents on the questions of the survey and the total duration to fill in the survey were taken into account as well. However, modifications were not needed and these parents and the public helped disseminating the survey on social media. We plan to disseminate the results to clinicians and health policy makers so that decisions concerning access to health services and support for parents can be made based on evidence.

Ethical considerations

The study was approved by the Medical Ethics Committee Erasmus Medical Centre (MEC-2020-0627) and by the Scientific Research Committee SARS-CoV-2 and COVID-19 Erasmus Medical Centre. Information about the study and its purpose was explained at the beginning of the online survey. The survey introduction also included a statement about the anonymity of the responses, which explained that no personal identifiable data would be collected. At the end of this information section parents were asked to check a statement, which clearly stated that choosing to complete and submit responses to the survey gave consent to their responses being used in our study. Since we were only including parents in the survey and not the

For peer review only

children themselves, there were no concerns about capacity to consent. Information on data usage and dissemination was also provided. Lastly, the respondents had the opportunity to contact the research team if they had additional questions or if they wanted to receive a summary of the study findings. Email addresses were provided for both the project lead and an independent paediatrician to whom any concerns or complaints could be directed.

Data analysis

Data were analysed using SPSS software version 25.0 and free text data was subjected to thematic analysis. First, descriptive statistics were used for the characteristics of parents and families who completed the survey. Information on living area, access to WiFi, children in the family, and whether they have laptops, computers or mobile phones, was collected. Secondly, descriptive statistics for children's characteristics, stratified for chronic condition, were used. The following question on chronic conditions was included in the survey: "Does your child have any pre-existing illnesses, such as chronic or long term illness, complex needs or a recurring illness?" When parents answered 'yes' they were asked to specify what kind of chronic condition or recurring illness their child had. Children's characteristics included age, gender, presenting symptom and hospital admission. Age was categorised into three predefined age groups: <5 years, 5-11 years and 12-17 years. Presenting symptoms were categorised into the following eight subgroups: skin, breathing, body temperature, dehydration, pain, change of behaviour, injury and other. Thirdly, parents' response to a sick or injured child was described, stratified for chronic condition. Parents were asked whether they would have sought help before lockdown and whether they did seek help during lockdown. Lastly, we analysed whether changes in health services during the lockdown affected severity of illness of the child and the child's treatment, stratified for chronic condition. McNemar's test and X^2-tests were used for quantitative analyses with a p-value <0.05 considered as statistically significant. Qualitative analyses using thematic analysis according to Braun and Clarke were used for free text answers from parents on questions regarding the impact of changes in health services on severity of illness of the child and the treatment the child received.(18) Two authors (CT and EL) categorised the free text answers independently to minimise subjectivity of a single researcher's judgement. The experiences of parents regarding the perceived impact of changes in health services were categorised into negative and positive responses. Furthermore, translated quotes of parents were given to show nuances in the data.

For peer review only - http://bmjopen.bmj.com/site/about/guidelines.xhtml

Results

A total of 105 respondents filled in the online COVID-19 parent survey in the Netherlands. During the two-month period from October 19th to December 19th 2020, 76 respondents completed the survey regarding the first lockdown period. During the second lockdown, an additional 29 respondents completed the survey from January 16th to February 16th 2021. Figure 1 shows through which social media channel the online survey had reached parents. The online survey reached 60% of the parents via Facebook (personal and groups), 28% via WhatsApp, and 12% via Twitter and LinkedIn.

Parent and family characteristics

The parent and family characteristics are shown in table 1. Most families came from small cities in an urban area (69%) and nearly half were from the province South-Holland (47%). All parents had access to WiFi, nearly all had laptops or computers (95%), mobile phones (90%) and access to outdoor space during lockdown (97%). The majority of the school aged children in the family were staying at home as schools were closed (76%) when the illness or injury happened during lockdown.

Table 1: Parent and family characteristics (N=105)

	**Parent of sick or injured child**
**Description of living area**	
Rural – village	6 (6)
Urban - small city	72 (69)
Urban – big city	27 (26)
**Province**	
South Holland	49 (47)
Zeeland	15 (14)
North Brabant	12 (11)
The other 7 provinces	29 (28)
**WiFi access**	105 (100)
**Mobile phone**	95 (90)
**Laptop or computer**	100 (95)
**Access to outdoor space during lockdown** (garden/balcony)	102 (97)

For peer review only

Where were the school aged children in the family when the illness/injury happened during the lockdown	
All of the children were staying at home	80 (76)
All the children were attending school	14 (13)
Some of the children were attending school	11 (11)

Absolute numbers and percentages (%) are shown

Children's characteristics

In table 2 the children's characteristics are shown for the total population and stratified for chronic condition. Forty nine percent of the children had a chronic condition or recurring illness (51/105) such as psychomotor retardation/epilepsy, scoliosis, asthma, eczema or recurring otitis media. The majority of the children were below the age of twelve and were boys. The most common presenting symptoms were pain (45%) and change of behaviour (40%). Children with chronic conditions were older and were more often reported to have breathing problems and fewer injuries compared to children without chronic conditions. Other presenting symptoms were for example hearing problems, congenital malformations (cleft lip, scoliosis), and facial nerve paralysis. Children with chronic conditions were less frequently admitted to the hospital during lockdown compared to children without chronic conditions (31% versus 39%).

Table 2: Children's characteristics stratified for chronic condition

	Total (N=105)	Children with chronic condition (N=51)	Children without chronic condition (N=54)
**Age** (years)			
<5	41 (39)	12 (24)	29 (54)
5-11	43 (41)	25 (49)	18 (33)
12-17	21 (20)	14 (28)	7 (13)
**Gender**			
Boys	61 (58)	28 (55)	33 (61)
Girls	44 (42)	23 (45)	21 (39)
**Presenting symptom***			

For peer review only

Skin and appearance	22 (21)	11 (22)	11 (20)
Breathing	25 (24)	15 (29)	10 (19)
Body temperature	16 (15)	9 (18)	7 (13)
Dehydration	21 (20)	11 (22)	10 (19)
Pain	45 (45)	24 (47)	23 (43)
Change of behaviour	42 (40)	21 (41)	21 (39)
Injury	23 (22)	8 (16)	15 (28)
Other	19 (18)	7 (14)	13 (24)
**Hospital admission**			
Admitted	37 (35)	16 (31)	21 (39)

Absolute numbers and percentages (%) are shown

* Possible to have more than 1 presenting symptom

Parents' response to a sick or injured child

Parents' response to a sick or injured child before and during lockdown, stratified for chronic condition, is shown in table 3. Parents reported that 83% (87/105) would have sought help before lockdown, whereas 88% (92/105) of the parents did seek help during lockdown. Four parents reported that they would have sought help before lockdown but did not seek help during lockdown, whereas nine parents would not have sought help before lockdown but did seek help during lockdown. (McNemar's test, p=0.2) *(Supplementary file 5 Appendix E).* The thirteen parents (13/105, 12%) who decided not to ask for medical help during lockdown stated that they were not sure if their child was ill or injured enough to need medical help, were worried about themselves or a family member catching COVID-19, thought that the advice "to stay at home" meant that they could not go to a health centre or hospital or were worried about using health services when they were needed more urgently by other people. Before the lockdown, parents of children with a chronic condition would have sought help for their sick or injured child in 77% (39/51) compared to 89% (48/54) of the parents of children without a chronic condition (X^2-test, p=0.1). During the lockdown, parents of children with a chronic condition did seek help for their sick or injured child in 82% (42/51) compared to 93% (50/54) of the parents of children without a chronic condition (X^2-test, p=0.1). Help seeking behaviour before and during lockdown related to children's characteristics is shown in Appendix F. *(Supplementary file 6)* Parents of children below the age of five sought help more frequently and the percentage of parents seeking help decreased with increasing child's

For peer review only

age. The percentage of parents seeking help for their sick or injured child was comparable for all presenting symptom groups except for the 'other' presenting symptom group.

Table 3: Parents' response to a sick or injured child reported by parents

	**Would have sought help before lockdown**	**Did seek help during lockdown**	**Total children**
**With chronic condition**	39 (77)	42 (82)	51
**Without chronic condition**	48 (89)	50 (93)	54
**Total**	87 (83)	92 (88)	105

Absolute numbers and percentages (%) are shown

The reported medical sources used by parents before lockdown were comparable with the reported sources used during lockdown. The majority of the parents contacted the GP and some parents sought help from their child's paediatrician or another medical professional such as the midwife. A small percentage of the parents called the Dutch emergency number or went to the ED. Parents were asked which other actions they undertook for their sick or injured child. Some parents stated that they chose to wait-and-see whether their child got better. When they did decide to treat the illness or injury themselves, they most often treated their child with paracetamol. Parents sought information on how to treat their child on the internet and by asking their social circle of friends and family. When parents stated that information and advice given were useful, this was mainly because they were reassured that what they were doing was right. A quote from a parent was "The information was useful since it reassured me, useful tips and information on when to seek medical help (again) were given". When parents stated that information and advice given were not useful, this was mainly because it was general advice and not child specific. A quote of a parent was "Every child is unique and requires an individual approach".

Impact of changes in health services

Parents reported that changes in health services during lockdown had impact on the severity of illness of their child in 31% (32/105) and on their child's treatment in 39% (41/105) (table 4). Parents' explanations were mostly negative and included the following responses: long

For peer review only

waiting list for diagnostics or treatment, postponed surgery or out-patient clinic consultation, no assessment by a physician, more consultations by telephone, fewer checks and doctor visits during admission. Lastly some parents stated they waited too long to seek help because they were anxious. Contrarily, there were also some positive responses including shorter turnaround time at the ED and more attention from medical staff during admission as the ward was almost empty. Parents of children with a chronic condition reported that changes in health services affected the severity of their child's illness in 33% (17/51) compared to 28% (15/54) of the parents of children without a chronic condition (X^2-test, p=0.5). Parents of children with a chronic condition reported that changes in health services affected their child's treatment in 47% (24/51) compared to 32% (17/54) of the parents of children without a chronic condition (X^2-test, p=0.1).

Table 4: Impact of changes in health services on severity of illness and child's treatment reported by parents

	**Changes in health services**		
	**Did affect child's severity of illness**	**Did affect child's treatment**	**Total children**
**With chronic condition**	17 (33)	24 (47)	51
**Without chronic condition**	15 (28)	17 (32)	54
**Total**	32 (31)	41 (39)	105

Absolute number are shown and percentages (%) are shown

Discussion and conclusion

Main findings

Parents in the Netherlands with a sick or injured child during the COVID-19 lockdown and who completed the online survey, were not deterred from seeking medical help for their sick or injured child. Almost 50% of the sick or injured children in our study had a chronic condition or recurring illness. Parents of children without a chronic condition would have sought help more often before lockdown and did seek help more frequently during lockdown than parents of children with a chronic condition. This might be explained by the fact that

For peer review only

parents of children with chronic conditions are better able to judge their child's illness or to treat the illness themselves since they have more medical experience. However, more parents of children with a chronic condition reported that changes in health services during lockdown had impact on their child's severity of illness and treatment compared to parents of children without a chronic condition. Parents of children with a chronic condition may have been more anxious during the COVID-19 pandemic about catching COVID-19 themselves or their child, who is perceived to be more vulnerable than a healthy child. The child's age was associated with parents' help seeking behaviour as parents of children below the age of five sought medical help more frequently, both before and during lockdown. This finding is also described by Sands et al. where the majority (70%) of medical attendees in children were below the age of five.(19) Remarkably, the number of parents who did seek medical help during the lockdown was high in all presenting symptom groups. This might indicate that parents who completed the survey probably had a more severely ill child. This could also explain the high hospital admission rate (35%) in our study. It has been reported that there is an increase in mental health problems in children due to the lockdown.(20) Where breathing problems are a common presenting symptom to seek medical help, 40% of the children in our study had a change of behaviour as presenting symptom which is typical for the lockdown period.(19)

Strengths and limitations

This online survey was the first national survey in the Netherlands on parents' help seeking behaviour and care for a sick or injured child during the lockdown period due to the COVID-19 pandemic. The survey was circulated through social media to reach the general population, which was also geographically spread throughout the Netherlands. The survey had several 'other' options where parents could give a more detailed explanation of their answers in addition to the multiple-choice answers. There were also some limitations. Firstly, the survey was advertised through social media, which could have caused selection bias since parents who do not have social media or who are not active on social media could not have filled in the survey. Parents with limited (digital) literacy would also have been unable to fill in the survey. The majority of the respondents live in the province South-Holland, which could be explained by the fact that ErasmusMC Sophia is located in South-Holland. Secondly, our study population might not be a good reflection of the general population of ill or injured children as almost 50% of the children had a chronic condition or recurring illness. This might be explained by the dissemination strategy or parents of children with a chronic condition

For peer review only

being more motivated to participate. However, this allowed us to perform analyses stratified for chronic condition which is independent of our sample of parents who had completed the survey. Furthermore, children with a chronic condition are a vulnerable group, who are more prone to serious illness and this study provides important insights in the help seeking behaviour of their parents.

Implications for clinical practice

This study showed that the majority of parents who completed our survey did seek help for a sick or injured child during the lockdown period. However, there were barriers in the help seeking process such as postponed doctors' visits, no physician's assessment and fewer face-to-face consultations were undertaken as more were performed by telephone. A small group of parents decided not to seek help for their sick or injured child because they were not sure if their child was ill or injured enough to need medical help reflecting pre-pandemic research findings (21), were worried about catching COVID-19 or thought that the advice to stay at home meant that they could not attend medical health services. This finding is important since parents should have adequate information on when and where to seek help for a sick or injured child. However, throughout 2020 and 2021, healthcare professionals have been contacting the Dutch media to emphasize the message that EDs and hospitals continue to be available to provide care and treatment for all patients if they need medical help.(22-24)

Conclusion

Parents in the Netherlands who completed the online survey about help seeking behaviour and care for a sick or injured child during the COVID-19 lockdown were not deterred from seeking medical help for their sick or injured child. However, changes in health services did affect their child's severity of illness and treatment, especially for children with a chronic condition. Although the impact was mostly negative there were some positive consequences as well. These findings are important to ensure that we inform parents, especially of children with chronic conditions, on when and where to seek help during lockdown periods to prevent delayed presentation of children with illness or injury to health services.

For peer review only

References

1. Isba R, Edge R, Jenner R, et al. Where have all the children gone? Decreases in paediatric emergency department attendances at the start of the COVID-19 pandemic of 2020. *Arch Dis Child.* 2020;105(7):704.

2. Raucci U, Musolino AM, Di Lallo D, et al. Impact of the COVID-19 pandemic on the Emergency Department of a tertiary children's hospital. *Ital J Pediatr.* 2021;47(1):21.

3. Morello F, Bima P, Ferreri E, et al. After the first wave and beyond lockdown: long-lasting changes in emergency department visit number, characteristics, diagnoses, and hospital admissions. *Intern Emerg Med.* 2021:1-8.

4. Sokoloff WC, Krief WI, Giusto KA, et al. Pediatric emergency department utilization during the COVID-19 pandemic in New York City. *Am J Emerg Med.* 2021;45:100-4.

5. Toet H, Sprik, E. & Blatter, B. Effecten van de Corona maatregelen op SEH-bezoeken? Stand van zaken tot en met september 2020. Kenniscentrum letselpreventie; 2020.

6. Zorgautoriteit dN. Analyse van de gevolgen van de coronacrisis voor de reguliere zorg 2020 [updated 29-04-2020. Available from: https://puc.overheid.nl/nza/doc/PUC_306627_22/1/.

7. Hassoun A, Pugh S, Merced I, et al. The power of social isolation on paediatric emergency visits during COVID-19 lockdown. *Acta Paediatr.* 2020;109(11):2425-6.

8. Lazzerini M, Barbi E, Apicella A, et al. Delayed access or provision of care in Italy resulting from fear of COVID-19. *Lancet Child Adolesc Health.* 2020;4(5):e10-e1.

9. Jansen D, Illy KE. Delayed presentation to regular Dutch paediatric care in COVID-19 times: a national survey. *BMJ Paediatr Open.* 2020;4(1):e000834.

10. Lynn RM, Avis JL, Lenton S, et al. Delayed access to care and late presentations in children during the COVID-19 pandemic: a snapshot survey of 4075 paediatricians in the UK and Ireland. *Arch Dis Child.* 2021;106(2):e8.

11. Ciacchini B, Tonioli F, Marciano C, et al. Reluctance to seek pediatric care during the COVID-19 pandemic and the risks of delayed diagnosis. *Ital J Pediatr.* 2020;46(1):87.

12. Ali MM, West K, Teich JL, et al. Utilization of Mental Health Services in Educational Setting by Adolescents in the United States. *J Sch Health.* 2019;89(5):393-401.

13. Golberstein E, Wen H, Miller BF. Coronavirus Disease 2019 (COVID-19) and Mental Health for Children and Adolescents. *JAMA Pediatr.* 2020;174(9):819-20.

14. Rauschenberg C, Schick A, Goetzl C, et al. Social isolation, mental health, and use of digital interventions in youth during the COVID-19 pandemic: a nationally representative survey. *Eur Psychiatry.* 2021:1-32.

For peer review only

15. Chen X, Qi H, Liu R, et al. Depression, anxiety and associated factors among Chinese adolescents during the COVID-19 outbreak: a comparison of two cross-sectional studies. *Transl Psychiatry*. 2021;11(1):148.

16. Raosoft. Sample size calculator: Raosoft Inc.; [Available from: http://www.raosoft.com/samplesize.html.

17. Systems CR. Sample Size Calculator: Creative Research Systems; [Available from: https://www.surveysystem.com/sscalc.htm.

18. Braun V, Clarke V. Using thematic analysis in psychology. *Qual Res Psychol* 2006:3: 77 - 101.

19. Sands R, Shanmugavadivel D, Stephenson T, et al. Medical problems presenting to paediatric emergency departments: 10 years on. *Emerg Med J*. 2012;29(5):379-82.

20. Newlove-Delgado T, McManus S, Sadler K, et al. Child mental health in England before and during the COVID-19 lockdown. *Lancet Psychiatry.* 2021.

21. Neill SJ, Jones CH, Lakhanpaul M, et al. Parents' help-seeking behaviours during acute childhood illness at home: A contribution to explanatory theory. *J Child Health Care.* 2016;20(1):77-86.

22. Willems I. Ziekenhuis St Jansdal slaat alarm over zorgmijders: 'Er staat hier écht geen meneer corona voor de deur': De Stentor; 2021 [updated 19-02-2021. Available from: https://www.destentor.nl/veluwe/ziekenhuis-st-jansdal-slaat-alarm-over-zorgmijders-er-staat-hier-echt-geen-meneer-corona-voor-de-deur-br~a76a3e50/?referrer=https%3A%2F%2Fwww.google.com%2F.

23. Sevil M. Huisartsen: kom met serieuze klachten wel langs: Het Parool; 2020 [updated 30-03-2020. Available from: https://www.parool.nl/nieuws/huisartsen-kom-met-serieuze-klachten-wel-langs~b8e4495b/.

24. NOS. Zorgen op spoedeisende hulp om wegblijvende patiënten: 'Kom vooral wel': NOS; 2020 [updated 27-03-2020. Available from: https://nos.nl/artikel/2328538-zorgen-op-spoedeisende-hulp-om-wegblijvende-patienten-kom-vooral-wel.html.

For peer review only

Text for figure 1:

Figure 1: Pie chart demonstrating through which social media channel the online survey had reached parents

Authors contributions

Chantal Tan and Eveline Lutgert contributed to the conceptualisation and design of the study, coordinated the data collection, carried out the analyses, and drafted the initial and final manuscript. Dorine Borensztajn, Sarah Neil, Rachel Carter, Ray Jones and Jade Chynoweth contributed to the conceptualisation and design of the study, contributed to the conceptualisation and design of the study, supervised the analyses and critically reviewed the manuscript. Henriette Moll contributed to conceptualisation and design of the study, contributed to interpretation of the data, critically reviewed the manuscript and supervised the study. All authors approved the final manuscript as submitted and agree to be accountable for all aspects of the work.

Funding statement:

This project has received funding from the European Union's Horizon 2020 research and innovation programme under grant agreement No. 848196. The funding sources had no role in the design and conduct of the study; collection, management, analysis, and interpretation of the data; preparation, review, or approval of the manuscript; and decision to submit the manuscript for publication.

Competing interests statement:

There are no competing interests.

Patient consent for publication:

Not required.

Ethics approval:

The study was approved by the Medical Ethics Committee Erasmus Medical Centre (MEC-2020-0627) and by the Scientific Research Committee SARS-CoV-2 and COVID-19 Erasmus Medical Centre.

For peer review only

Data availability statement:

Data are available upon request. Data from this study are available upon request to the corresponding author of the study (c.tan@erasmusmc.nl), subject to local rules and regulations.

For peer review only

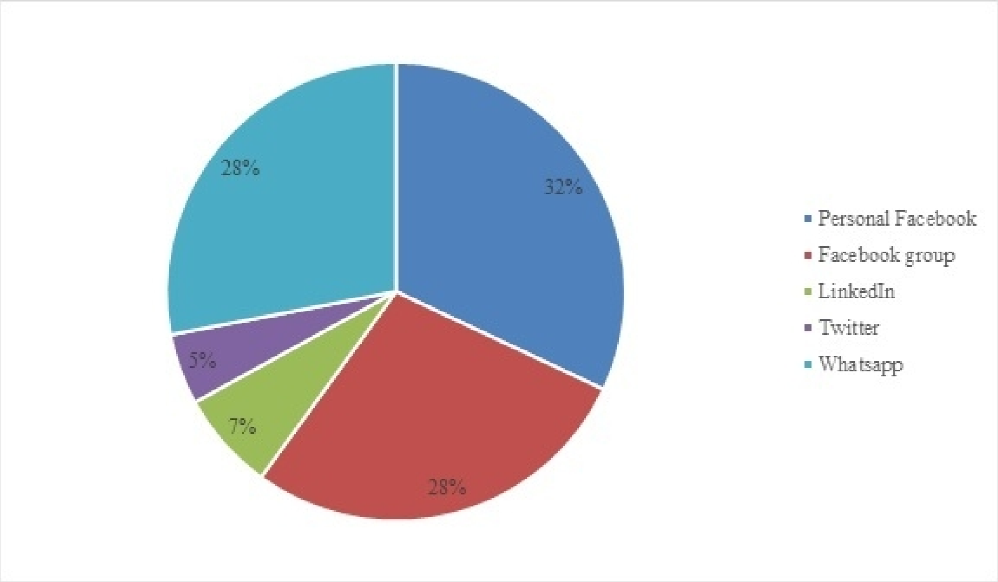

Figure 1: Pie chart demonstrating through which social media channel the online survey had reached parents

48x28mm (300 x 300 DPI)

SUPPLEMENTARY FILE 1

Appendix A: Timeline of measures taken during the first and second lockdown period

**Date**	**Measures**
	First lockdown March 23rd – June 1st 2020
March 23rd	- > 2 persons in public area prohibited with the exception of one household<br>- Group formation only with 1.5 meter distancing rule, maximum 100 persons<br>- Closing of catering industries and schools<br>- Contact professions prohibited<br>- Stores open with 1.5 meter distancing rule and hygiene measures<br>- Households can have maximum of 3 persons visiting them with 1.5 meter distancing rule<br>- Work from home as much as possible<br>- In case of violation 390 euro fine, companies 4350 euro fine
May 11th	- Children day-care and primary schools reopening (in smaller groups)<br>- Contact professions reopening<br>- Playing sports outside allowed with 1.5 meter distance<br>- Opening libraries
June 1st	- Face mask mandatory in public transport, only use public transport when necessary<br>- Restaurants / catering industries reopening for max 30 persons, booking in advance<br>- Museums, cinemas reopening for max 100 persons<br>- High school reopening
June 8th	- Primary school fully reopened
August 6th	- Announcement of the extension of a flight restriction to European countries, the United Kingdom, all countries on the Western hemisphere.
October 14th	- Closing of catering industries

For peer review only

	- A ban on selling alcohol between 8 pm and 7 am<br>- Maximum of 3 visitors per home, maximum of 30 people in interior spaces, outside no more than 4 people from other households.<br>- A ban on late night shopping
December 1st	- Facemasks become mandatory in public places, in schools, in public transport and for contact professions
	Second lockdown December 15th 2020 - February 16th 2021
December 15th	- Closing of non-essential shops<br>- Closing of theatres, cinemas, museums, zoo's, sauna's and libraries<br>- Contact professions closing (not including physiotherapists)<br>- Closing of restaurants and schools (schools starting from December 16th)<br>- Households can have maximal 2 persons visiting them with 1.5 meter distancing rule (on Christmas a maximum of 3 persons)<br>- Work from home as much as possible
December 29th	- Travellers to the Netherlands need to provide a negative PCR-test (not older than 72 hours)
January 6th	- Start of the vaccination program (healthcare workers from the emergency medicine)
January 23rd	- Mandatory curfew from 09 pm until 04.30 am
February 8th	Primary school and day-care reopening

For peer review only

SUPPLEMENTARY FILE 2

Appendix B: Original English version of the online survey

Thank you for helping us with our survey. We would like to find out what impact the Stay Home period is having on parent's actions and decision when caring for, or seeking help for, a sick or injured child. The Stay Home period is sometimes called the lockdown that started on the 23rd March when the government put restrictions on movements to stop the spread of COVID-19. The findings will help us to learn from parents' experiences and direct the development of support for parents with a sick or injured child in the future.

If your child has been ill or injured while you have been following government advice to Stay Home, we would be most grateful if you could complete our survey. We would like to know about your experiences of caring for a sick or injured child at this time, whether or not you decided to seek medical help. We would like parents to complete the survey whether or not their child needed hospital care.

If you do kindly complete and submit the survey this will indicate that you are happy for us to use your responses in our project. Your responses are, of course, anonymous so it will not be possible to identify your replies to remove them after you have completed the survey. Your survey responses will go directly to the survey team.

We will write up the findings from the project and share these in academic journals, in communication with health service leaders and policy makers, at conferences, in education, in future research projects and online. If you provide any free text comments your words may be used but it will not be possible to identify you. If you would like a summary of our findings please contact one of the project leads.

The leads for this project are Professor Sarah Neill sarah.neill@plymouth.ac.uk and Rachel Carter r.carter@plymouth.ac.uk

I can confirm that I have read the information above, I am happy to complete the questionnaire and I am happy for the information I give to be used for the project. Tick box

Are you a UK resident with a child who has been ill or injured since you were asked to Stay Home?

Yes – directed to the questionnaire

No - we thank you for your interest but we are only looking for parents living in the UK whose children have been ill or injured whilst being asked to Stay Home. We hope that you and your family stay well during this period.

For peer review only

It may be that your child has had a number of illnesses or injuries in the last few weeks since you were asked to stay at home. We would like you to select the most serious of these and answer the remaining questions with that in mind.

Question 1 About the child who was ill or injured

a. How old is your child?
    - o Under 12 months old
    - o 12 months and under 24 months old
    - o 2 years and under 5 years old
    - o 5 years and under 12 years old
    - o 12 years and under 16 years old
    - o 16 and under 18 years old

b. Was your child born a girl or boy?
    Girl
    Boy

c. Does your child have any pre-existing illnesses, such as chronic or long term illness, complex needs or a recurring illness?

If yes, please tell us what they are:

Question 2 What signs or symptoms of illness or injury did your child have? Please tick all that apply

Skin and appearance
- Skin very pale or blue, or the inside of their lips and tongue are blue
- Skin appeared mottled
- Rash which disappeared when pressed
- Rash which did not disappear with pressure (the ‘Glass test’)

Breathing difficulties
- Breathing stopped for a long time (more than 10 seconds at a time),
- Breathing paused (apnoeic episodes) for 5-10 seconds
- Struggling to breathe
- Sucking in muscles under their ribs, between the ribs or at the neck with each breath
- Grunting with the effort of trying to breathe
- Breathing faster than normal
- Noisy breathing (wheeze or a stridor)

Body temperature
- Under 3 months of age with a temperature of over 38°C/100.4^{o}F
- 3 and 6 months of age with a temperature over 39°C/102.2^{o}F

For peer review only

- Over 6 months old with a temperature above 38ºC/100.4ºF for more than 5 days
- Under 1 month of age and had a low temperature below 36ºC/96.8ºF

Dehydration (seemed to be too dry)

- Dry mouth
- Sunken eyes
- No tears
- Drowsy
- Passing less urine than usual
- Passing more urine than usual
- Drinking more than normal

Pain

- Tummy pain
- Back pain
- Painful testicles
- Headache
- Persistent pain in an injured arm or leg
- Earache
- Sore throat

Change in behaviour

- Agitated
- Persistent crying/inconsolable
- Fit/seizure
- Confused
- Wouldn't wake up or, if woken up, they wouldn't stay awake
- Unresponsive

Injury

- Burn
- Head injury
- Leg or arm injury
- Fall
- Cut(s)
- Bleeding
- Limping
- Difficulty in moving a part of the body

Other, please give details

For peer review only

Question 3 Before the advice to Stay Home, what would your *usual* response have been to this illness or injury? (Please tick one only)

- Care for my child at home
- Ask for medical help
- Something else, please give details

If you would *normally* have asked for medical help please go to Question 4

If you would *not normally* have asked for medical help please go to Question 5

If you answered *something else*, please go to Question 5

Question 4 Where would you *normally* have asked for medical help for your child with this illness or injury <u>in the first instance</u>? (Choose one only)

- NHS Direct 111
- GP surgery
- Via my GP's website
- Video consultation
- Walk in centre
- Urgent care centre
- Minor injuries unit
- GP Out of Hours service
- Calling 999
- Accident and Emergency
- Somewhere else, please say where……

Question 5 Did you ask for medical help for your child during this most recent illness or injury?

Yes, please continue to Question 6

No, please go to Question 9

Question 6 Where did you ask for medical help during this most recent illness or injury? (Please tick all that apply)

- NHS Direct 111
- GP surgery
- Via my GP's website

For peer review only

- Video consultation
- Walk in centre
- Urgent care centre
- Minor injuries unit
- GP Out of Hours service
- Called 999
- Accident and Emergency
- Somewhere else, please say where……

Question 7 Was your child admitted to hospital for this most recent illness or injury? Yes/No

If yes go to question 10

If no, continue to the next question

Question 8 What, if anything else, would you like to tell us about your experience, for example, whether advice was helpful or not helpful.

Go to Question 10 for all respondents

Question 9 Why did you decide not to ask for medical help? Please tick all that apply

- I was not sure if my child was ill or injured enough to need medical help
- I was worried about myself or a member of my family catching COVID19
- I was worried that I might be criticised for using the service if it wasn't an emergency
- I thought the Stay Home advice meant I couldn't go to a health centre or a hospital
- I was worried about using a service when it is needed more urgently by other people
- I was worried that the service would be extremely busy and that I would have to wait for too long
- I don't have a car and didn't want to use public transport
- I don't have anyone to look after my other children
- Something else - please give details

Question 10 What else did you do about your child's illness or injury? Please tick all that apply

a. I waited to see whether or not my child got better
b. I treated my child's illness/injury myself with:
    - paracetamol (Calpol)
    - ibuprofen (Brufen/Junifen)

For peer review only

- a prescribed inhaler
- Vicks or Karvol
- A cold compress/ice pack
- Cleansing
- Bandaging
- A sling to rest an injured arm
- Elevated an injured limb
- Cold drinks
- Warm drinks
- Cool bath
- Warm bath
- Treats and attention
- Distracting my child
- Home remedies, please explain what these were
- Other, please tell us what this was

c. I looked for information on how to manage the illness or injury at home from:

- family
- friends
- a family health professional
- books
- TV
- magazines
- newspapers
- radio
- the internet
- an NHS app
- Other app
- Social media (such as Twitter, Facebook, Instagram, YouTube)
- Google
- NHS Choices
- GP website
- Other website
- Other: please state

d. I used information I already had from (tick all that apply):

- family
- friends
- a family health professional
- books

For peer review only

- TV
- Magazines
- Newspapers
- radio
- an NHS app
- Other app
- Social media (such as Twitter, Facebook, Instagram, YouTube)
- Google
- NHS Choices
- GP website
- Other website
- School or college course
- A mixture of these resources
- Other: please state

Question 11 Helpfulness of information

a. What, if any, information was helpful and if so why?

b. What, if any, information was unhelpful and if so why?

Question 12 Advice provided during the Stay Home period

a. Which of the following sources of advice have you seen about when to use health services while families are being told to Stay Home? Please tick all that apply

- Government sources (such as Government TV or radio broadcast, the letter from Boris Johnson, daily briefings from Government)
- NHS sources (such as NHS Choices website, GP website, 111 service announcements)
- Other expert online sources (such as University, research organisations etc)
- Experts on Social Media sites (such as Twitter, Facebook)
    - Family/Friends on Social Media sites
    - Other, please give details

b. Have you seen advice about when to (Please tick all that apply)
    - call NHS111?
    - call your GP/family doctor?
    - attend accident and emergency with your children?

c. What, if anything, was new about advice on health service use since you have been told to Stay Home?

For peer review only

Question 13 Have the changes to health services during the Stay Home period affected how ill your child has been? Yes, Maybe, No

d. If yes, please tell us how.

Question 14 Have the changes to health services during the Stay Home period affected any treatment your child received? Yes, Maybe, No, Not applicable

e. If yes, please us how.

Please tell us about your family

Question 15 How old are the children in the household?

**Age of children**	**Please tell us the number of children in each age group**
0-4 years	
5-11 years	
12-15 years	
16-18 years	

Question 16 What area do you live in?

- Scotland
- Wales
- Northern Ireland
- East of England
- London
- Midlands
- North East and Yorkshire
- North West
- South East
- South West

Question 17 Which of the following best describes the area in which you live?

- Rural
- Urban

Questions 18 What access do you have to technology: Please tick all that apply

For peer review only

a. What type of mobile phone do you have?
   - for calls and texting only
   - Smartphone

b. What type of mobile phone contract do you have?
   - Pay as you go mobile phone
   - Monthly contract mobile phone

c. What type of computer do you have?
   - laptop
   - desktop computer

d. How much WiFi access do you have?
   - Unlimited WiFi access
   - Limited WiFi access
   - Other access to the internet, please state

Question 19 Do you have access to outdoor space? Yes/No

Question 20 We know that some children are still able to attend school. Where were the school aged children in your family when the illness or injury happened?

- All of my children were attending school
- Some of my children were attending school
- All of my children were staying at home

Question 21 What if, anything else, would you like to tell the project team?

Thank you for taking the time to complete our questionnaire.

If you would like to receive a summary of the project findings please email one of the project leads:

Professor Sarah Neill *sarah.neill@plymouth.ac.uk* or Rachel Carter *r.carter@plymouth.ac.uk*

If you have any concerns or complaints about the project please contact the Research Administrator to the Faculty Research Ethics and Integrity Committee (*hhsethics@plymouth.ac.uk*)

For peer review only

SUPPLEMENTARY FILE 3

Appendix C: Adapted Dutch version of the online survey

Bedankt voor uw hulp bij onze enquête. Wij willen graag weten wat voor gevolgen de "lockdown periode" heeft gehad op de acties en beslissingen van ouders bij de zorg of het zoeken van hulp voor een ziek of gewond kind. Onze "intelligente lockdown" ging in op 23 maart 2020 toen de overheid beperkingen oplegde om zo de verspreiding van COVID-19 te stoppen. De resultaten zullen ons helpen om te leren van de ervaringen van ouders en de ondersteuning voor ouders met een ziek of gewond kind in de toekomst te verbeteren.

Indien uw kind ziek of gewond was terwijl u het advies van de overheid opvolgde om zoveel mogelijk thuis te blijven gedurende de intelligente lockdown periode van 23 maart tot 1 juni 2020, zouden we u zeer dankbaar zijn als u onze enquête wilt invullen. We willen weten wat uw ervaringen zijn met de zorg voor een ziek of gewond kind gedurende deze periode en of u besloot om wel of niet medische hulp te zoeken. We willen graag weten of uw kind een vorm van gezondheidszorg (zoals het raadplegen van de huisarts, de huisartsenpost of ziekenhuiszorg) nodig had.

Indien u de enquête volledig invult en indient, geeft u toestemming aan ons om uw antwoorden voor ons onderzoek te gebruiken. Uw antwoorden zijn volledig anoniem en niet naar u of uw kind te herleiden. Uw antwoorden gaan rechtstreeks naar het onderzoeksteam.

De enquête bestaat uit ongeveer 20 vragen en het invullen hiervan zal maximaal 10 minuten duren.

We zullen de resultaten van het onderzoek delen in wetenschappelijke tijdschriften en websites, in communicatie met artsen en beleidsmakers, op congressen, in onderwijs en in toekomstige onderzoeksprojecten. Als u opmerkingen in de vrije tekst invult, kunnen uw woorden worden gebruikt, maar is het niet mogelijk u te identificeren. Indien u een samenvatting van onze resultaten wilt, neem dan contact op met een van de projectleiders.

Dit project is een samenwerking tussen het Erasmus MC Sophia Kinderziekenhuis in Nederland en University of Plymouth en University College London in Engeland. Deze studie is goedgekeurd door de Commissie Wetenschappelijk Onderzoek COVID-19 van het Erasmus Medisch Centrum te Rotterdam (CWO COVID-19 EMC).

De projectleiders zijn professor Henriette Moll, kinderarts Dorine Borensztajn en arts-onderzoeker Chantal Tan.

Indien u nog vragen heeft over het onderzoek, kunt u contact opnemen met de projectleiders door een mail te sturen naar *ervaringen.ouders@erasmusmc.nl*.

For peer review only

Introductie vragen over deelname aan enquête en manier van werving

1) Ik kan bevestigen dat ik bovenstaande informatie heb gelezen en ik wil graag de enquête invullen en informatie geven voor het onderzoek.

- Ja
- Nee

2) Bent u een inwoner van Nederland en was uw kind ziek of gewond kind terwijl u het advies van de overheid opvolgde om zoveel mogelijk thuis te blijven gedurende de lockdown periode?

- Ja – u wordt doorverwezen naar de vragenlijst
- Nee – we danken u voor uw interesse, maar we zijn alleen op zoek naar ouders die in Nederland wonen en wiens kind(eren) ziek of gewond was tijdens de lockdown periode.

3). Hoe bent u bij deze vragenlijst terecht gekomen?

- Ik zag een bericht met de link naar dit formulier in een Facebook groep voor ouders/moeders
- Ik zag een bericht met de link naar dit formulier via-via op Facebook
- Ik zag een bericht met de link naar dit formulier op LinkedIn
- Ik zag een bericht met de link naar dit formulier op Twitter
- Ik kreeg een bericht met de link naar dit formulier via WhatsApp toegestuurd

1. Vragen over het zieke of gewonde kind

Het kan zijn dat uw kind meerdere ziekte episodes of verwondingen heeft gehad tijdens de "lockdown periode". We willen graag dat u de meest ernstige hiervan selecteert en de volgende vragen met die ziekte of verwonding in gedachte beantwoordt.

a). Hoe oud was uw kind ten tijde van de ziekte of verwonding?

- Jonger dan 12 maanden oud
- 12 maanden of ouders maar jonger dan 24 maanden oud
- 2 jaar of ouders maar jonger dan 5 jaar oud
- 5 jaar of ouder maar jonger dan 12 jaar oud
- 12 jaar of ouders maar jonger dan 16 jaar oud
- 16 of 17 jaar oud

b). Is uw kind een jongen of een meisje?

- Meisje
- Jongen

c). Is uw kind bekend met reeds bestaande ziekten, zoals chronische of langdurige ziekten, complexe behoeften of terugkerende ziekten?
Indien ja, graag toelichting welke ziekte(n) dit zijn:

For peer review only

2. Welke kenmerken of symptomen van ziekte of verwonding had uw kind.
Vink alles aan wat van toepassing is

- Huid en uiterlijk
    - Huid erg bleek of blauw, of binnenkant van de lippen en tong blauw
    - Vlekkerige huid
    - Uitslag die verdween wanneer erop werd gedrukt
    - Uitslag die niet verdween wanneer erop werd gedrukt
- Ademhalingsproblemen
    - Ademhaling stopte voor een lange periode (>10 seconden per keer)
    - Ademhaling onderbroken (ademstops) voor 5-10 seconden
    - Moeite hebben met ademen
    - Intrekkingen in de hals, en/of onder of tussen de ribben bij elke ademhaling
    - Kreunen bij de ademhaling
    - Sneller ademen dan normaal
    - Hoorbare ademhaling (gierende of piepende ademhaling)
- Lichaamstemperatuur
    - Jonger dan 3 maanden oud met een temperatuur boven de 38oC
    - Tussen de 3 en 6 maanden oud met een temperatuur boven de 39oC
    - Ouder dan 6 maanden oud met een temperatuur boven de 38oC voor langer dan 5 dagen
    - Jonger dan 1 maand oud met een temperatuur onder de 36oC
- Uitdroging
    - Droge mond
    - Ingevallen ogen
    - Geen tranen
    - Suf
    - Minder plassen dan normaal
    - Meer plassen dan normaal
    - Minder drinken dan normaal
    - Meer drinken dan normaal
- Pijn
    - Buikpijn
    - Rugpijn
    - Pijnlijke testikels
    - Hoofdpijn
    - Persisterende pijn in een gewond arm of been
    - Oorpijn
    - Keelpijn

For peer review only

- Verandering in gedrag
  - Geagiteerd (sneller boos of geïrriteerd zijn)
  - Aanhoudend huilen/ontroostbaar
  - Toeval of epileptische aanval
  - Verward
  - Niet wakker worden of niet wakker kunnen blijven
  - Niet reagerend
- Verwonding
  - Verbranding
  - Hoofdverwonding
  - Verwonding aan been of arm
  - Val
  - Snijwond
  - Bloeding
  - Mank lopen
  - Moeite om een lichaamsdeel te bewegen
- Andere ziekte of verwonding

Mocht de ziekte en/of verwonding van uw kind niet hierboven vermeld staan, graag hieronder uw toelichting

3. Wat zou uw gebruikelijke reactie zijn op deze ziekte of verwonding zijn geweest vóór de intelligente lockdown periode?

- Om medische hulp vragen → ga naar vraag 4
- Thuis voor mijn kind zorgen → ga naar vraag 5
- Anders → ga naar vraag 5

4. Waar zou u normaliter allereerst om medische hulp hebben gevraagd voor uw kind bij deze ziekte of verwonding?

- Eigen waarnemend huisarts (overdag)
- Telefonisch consult met de huisarts
- Inloop spreekuur bij de huisarts
- Website eigen huisarts
- Website thuisarts.nl
- Huisartsenpost (in de avond/nacht)
- Behandelend kinderarts, waar mijn kind onder controle is
- 112 bellen
- Spoedeisende hulp
- Anders

5. Heeft u om medische hulp gevraagd voor de ziekte of verwonding van uw kind tijdens de lockdown?

- Ja → ga naar vraag 6
- Nee → ga naar vraag 9

6. Waar heeft u om medische hulp gevraagd voor deze ziekte of verwonding tijdens de lockdown?

Vink alles aan wat van toepassing is

- Eigen waarnemend huisarts (overdag)
- Telefonisch consult met de huisarts
- Inloop spreekuur bij de huisarts
- Website eigen huisarts
- Website thuisarts.nl
- Huisartsenpost (in de avond/nacht)
- Behandelend kinderarts, waar mijn kind onder controle is
- 112 bellen
- Spoedeisende hulp
- Anders

7. Was uw kind opgenomen in het ziekenhuis vanwege de ziekte of verwonding tijdens de lockdown?

8. Wat zou u nog willen vertellen over uw ervaring? Bijvoorbeeld of het advies wat u heeft gekregen nuttig was of niet. → ga naar vraag 10

9. Waarom had u besloten om geen medische hulp te vragen? Vink alles aan wat van toepassing is

- Ik wist niet zeker of mijn kind ziek of gewond genoeg was om medische hulp te zoeken
- Ik was bezorgd dat ikzelf, mijn kind of een familielid COVID-19 zou krijgen
- Ik was bezorgd dat ik kritiek zou krijgen als ik medische zorg zou gebruiken als het geen noodgeval was
- Ik dacht dat het advies om zoveel mogelijk thuis te blijven tijdens de lockdown periode betekende dat ik niet naar een gezondheidsinstelling of ziekenhuis kon gaan
- Ik maakte me zorgen over het gebruik van medische zorg wanneer deze voor andere mensen meer nodig was
- Ik was bang dat de gezondheidsinstelling (huisarts of ziekenhuis) extreem druk zou zijn en dat ik lang zou moeten wachten
- Ik heb geen auto en ik wilde geen gebruik maken van het openbaar vervoer
- Ik heb niemand die op mijn andere kinderen kan passen

For peer review only

- Anders

10. Wat heeft u nog meer gedaan aan de ziekte of verwonding van uw kind?

- Ik heb gewacht om aan te kijken of mijn kind al dan niet beter werd
- Ik heb de ziekte of verwonding van mijn kind zelf behandeld. Vink alles aan wat van toepassing is
  - Paracetamol
  - Ibuprofen
  - Een voorgeschreven puffer
  - Vicks
  - Een koude kompres/koeling met ijs
  - Schoonmaken van de wond
  - Verbinden
  - Een draagdoek om een gewond arm te laten rusten
  - Een gewond ledemaat ondersteunen door het hoog te houden
  - Koude dranken
  - Warme dranken
  - Koud bad
  - Warm bad
  - Traktaties en aandacht
  - Mijn kind afleiden
  - Huismiddeltjes, graag uw toelichting
  - Anders
- Ik heb informatie gezocht over hoe ik thuis de ziekte of verwonding kon behandelen**.** Kunt u toelichten waar of via wie u deze informatie heeft opgezocht?
  - Familie
  - Vrienden
  - Een vriend of familielid die zorgmedewerker is
  - Boeken
  - TV
  - Tijdschriften
  - Kranten
  - Radio
  - Het internet
  - App: moet ik naar de dokter?
  - Andere apps
  - Sociale media (zoals Twitter, Facebook, Instagram, Youtube)
  - Google
  - Thuisarts.nl
  - Een andere website

For peer review only

   - Anders
- Ik heb informatie gebruikt welke ik al had. Kunt u toelichten waarvan of van wie u deze informatie al had gekregen?
   - Familie
   - Vrienden
   - Een vriend of familielid die zorgmedewerker is
   - Boeken
   - TV
   - Tijdschriften
   - Kranten
   - Radio
   - Het internet
   - App: moet ik naar de dokter?
   - Andere apps
   - Sociale media (zoals Twitter, Facebook, Instagram, Youtube)
   - Google
   - Thuisarts.nl
   - Een andere website
   - Anders
   -
- Niet van toepassing

11. Als de gevonden informatie nuttig was om u te helpen met de ziekte of verwonding van uw kind, wat vond u hier nuttig aan en waarom?
Als de gevonden informatie niet nuttig was, wat vond u hier niet nuttig aan en waarom?

12. Welke van de volgende bronnen voor advies bent u tegen gekomen wanneer u medische zorg nodig had gedurende de lockdown. Vink alles aan wat van toepassing is.

- Overheidsbronnen (dagelijks nieuws van overheid/RIVM)
- Gezondheidszorg bronnen (zoals website van uw eigen huisarts, thuisarts.nl, app moet ik naar de dokter?)
- Andere online expert bronnen (zoals universitaire of onderzoeksorganisaties etc)
- Deskundigen op sociale media (zoals Twitter, Facebook)
- Familie/vrienden op sociale media
- Anders

Heeft u advies gevonden in deze bron(nen) over wanneer u…

   - ... de huisartsenpost moest bellen?
   - … uw eigen huisarts moet bellen?

For peer review only

- … naar de spoedeisende hulp moet komen met uw kind?

Wat was nieuw aan het advies wat u heeft gezien omtrent het gebruik van medische zorg gedurende de lockdown?

13. Hebben veranderingen in de medische zorg gedurende de lockdown invloed gehad op hoe ziek uw kind was?

- Ja → graag uw toelichting
- Nee

14. Hebben veranderingen in medische zorg gedurende de lockdown invloed gehad op de behandeling die uw kind heeft gekregen?

- Ja → graag uw toelichting
- Nee
- Misschien
- Niet van toepassing

15. Hoe oud waren de kinderen in uw huishouden gedurende de lockdown periode? Vul het aantal kinderen per leeftijdsgroep in

- Leeftijd van de kinderen 0-4 jaar
- Leeftijd van de kinderen 5-11 jaar
- Leeftijd van de kinderen 12-15 jaar
- Leeftijd van de kinderen 16-17 jaar

16. In welke provincie woont u?

- Drenthe
- Flevoland
- Friesland
- Gelderland
- Groningen
- Limburg
- Noord-Brabant
- Noord-Holland
- Overijssel
- Utrecht
- Zeeland
- Zuid-Holland

Welke van de volgende opties omschrijft uw woonomgeving het beste?

For peer review only

- Grote stad
- Kleine stad/dorp
- Platteland

Had u de mogelijkheid om tijdens de lockdown buiten in uw tuin of op uw balkon te zijn?

- Ja
- Nee

17. Welke toegang heeft u tot technologie?

Wat voor mobiele telefoon heeft u ?

- Prepaid mobiele telefoon
- Maand abonnement
- Niet van toepassing

Wat voor soort computer heeft u?

- Een laptop
- Computer
- Ik heb geen computer

In welke mate heeft u toegang tot WiFi?

- Onbeperkte WiFi toegang
- Beperkte WiFi toegang
- Geen WiFi toegang
- Anders

18. We weten dan sommige kinderen nog naar school konden gaan. Waar waren de schoolgaande kinderen in uw gezin wanneer ze ziek of gewond waren gedurende de lockdown?

- Al mijn kinderen gingen naar school
- Sommige van mijn kinderen gingen naar school
- Al mijn kinderen bleven thuis

19. Heeft u nog opmerkingen voor het onderzoeksteam?

Hartelijk dank voor uw tijd en interesse om deze vragenlijst te beantwoorden.

For peer review only

Indien u een samenvatting van de bevindingen van het onderzoek zou willen ontvangen, stuur dan een mail naar de projectleider: Professor Henriette Moll (*ervaringen.ouders@erasmusmc.nl*)

Indien u enige zorgen of klachten heeft over het onderzoek, neem dan graag contact op met een onafhankelijk kinderarts (*secretariaatalkg@erasmusmc.nl*)

For peer review only

SUPPLEMENTARY FILE 4

Appendix D: Introduction of the survey on social media

Advertising text:

De coronaperiode is een zware dobber voor ouders. Is uw kind ziek of gewond geweest in de lockdown periode en wilt u bijdragen aan onderzoek van het Sophia Kinderziekenhuis, vul dan deze online enquête anoniem in (maximale duur 10 minuten):

h*ttps://forms.gle/GNyiP7G2xKnoSR9j8*

Alvast bedankt voor uw medewerking. Delen van dit bericht wordt zeer gewaardeerd!

Translation of advertising text:

The corona period is a tough one for parents. If your child has been ill or injured during the lockdown period and you want to contribute to research of the Sophia Children's Hospital, please complete this online survey anonymously (maximum duration 10 minutes):

https://forms.gle /GNyiP7G2xKnoSR9j8

Thank you in advance for your cooperation. Sharing this post is much appreciated!

Advertising image:

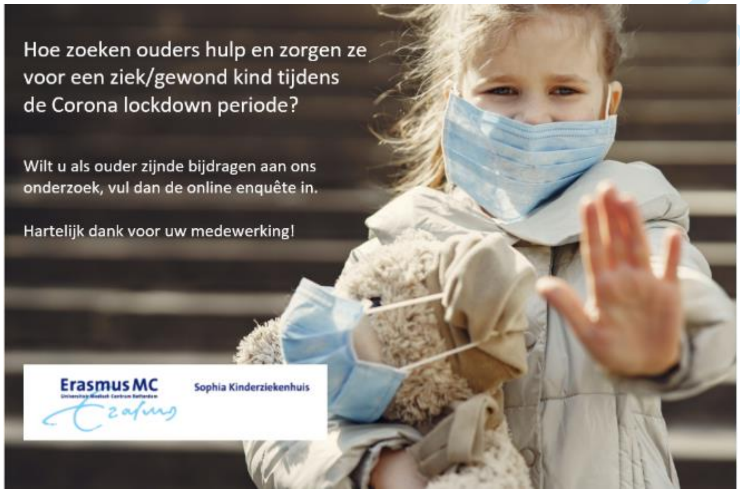

Translation of text on advertising image:

How do parents seek help and care for a sick or injured child during the corona lockdown period? Do you as a parent want to contribute to research, please fill in this online survey. Thank you very much for your cooperation!

For peer review only

SUPPLEMENTARY FILE 5

Appendix E: Table for McNemar's test

		Did seek help during lockdown		
**Would have sought help before lockdown**		*Yes*	*No*	*Total children*
	Yes	*83*	*4*	*87*
	No	*9*	*9*	*18*
	Total children	*92*	*13*	*105*

McNemar's test p=0.2

For peer review only

SUPPLEMENTARY FILE 6

Appendix F: Parents' help seeking behaviour related to children's characteristics

	**Sick/injured children (N=105)**	**Would have sought help before lockdown ¥**	**Did seek help during lockdown ¥**
**Age** (years)			
<5	41 (39)	37 (90)	39 (95)
5-11	43 (41)	33 (76)	36 (84)
12-17	21(20)	17(81)	17 (81)
**Gender**			
Boys	61 (58)	49 (80)	52 (85)
Girls	44 (42)	38 (86)	40 (91)
**Presenting symptom***			
Skin	22 (21)	18 (82)	21 (95)
Breathing	25 (24)	21 (84)	24 (96)
Body temperature	16 (15)	16 (100)	16 (100)
Dehydration	21 (20)	18 (86)	20 (95)
Pain	45 (45)	40 (89)	44 (94)
Change of behaviour	42 (40)	32 (76)	38 (91)
Injury	23 (22)	19(83)	23 (100)
Other	19 (18)	17 (89)	15 (75)
**Hospital admission**	37 (35)	36 (97)	37 (100)

Absolute numbers and percentages (%) are shown

* Possible to have more than 1 presenting symptom

¥ Percentage of children in that specific variable group

For peer review only

STROBE 2007 (v4) checklist of items to be included in reports of observational studies in epidemiology*
Checklist for cohort, case-control, and cross-sectional studies (combined)

Section/Topic	Item #	Recommendation	Reported on page #
**Title and abstract**	1	(*a*) Indicate the study's design with a commonly used term in the title or the abstract	The study design is stated in the title and abstract.
		(*b*) Provide in the abstract an informative and balanced summary of what was done and what was found	The abstract is written according to the *BMJ Open* guidelines with all required information.
**Introduction**			
Background/rationale	2	Explain the scientific background and rationale for the investigation being reported	The background and rationale are described in the first three paragraphs of the introduction.
Objectives	3	State specific objectives, including any pre-specified hypotheses	The objective is stated in the last paragraph of the introduction.
**Methods**			
Study design	4	Present key elements of study design early in the paper	Key elements of the study design are presented in the first paragraph of the methods section, and in detail explained in the consequent paragraphs.

For peer review only

Setting	5	Describe the setting, locations, and relevant dates, including periods of recruitment, exposure, follow-up, and data collection	The setting, location and relevant dates are described in the paragraphs 'Study population and setting' and 'Data collection' in the methods section.
Participants	6	(*a*) *Cohort study*—Give the eligibility criteria, and the sources and methods of selection of participants. Describe methods of follow-up<br>*Case-control study*—Give the eligibility criteria, and the sources and methods of case ascertainment and control selection. Give the rationale for the choice of cases and controls<br>*Cross-sectional study*—Give the eligibility criteria, and the sources and methods of selection of participants	Described in the paragraphs 'Study population and setting' and 'data collection' in the methods section. There was no follow-up.
		(*b*) *Cohort study*—For matched studies, give matching criteria and number of exposed and unexposed<br>*Case-control study*—For matched studies, give matching criteria and the number of controls per case	Not applicable.
Variables	7	Clearly define all outcomes, exposures, predictors, potential confounders, and effect modifiers. Give diagnostic criteria, if applicable	Described in the paragraphs 'Data collection' and 'Data analysis'.
Data sources/ measurement	8*	For each variable of interest, give sources of data and details of methods of assessment (measurement). Describe comparability of assessment methods if there is more than one group	Described in the paragraphs 'Data analysis'.
Bias	9	Describe any efforts to address potential sources of bias	The survey was disseminated via social media to reach the general population (minimise selection bias).
Study size	10	Explain how the study size was arrived at	A sample size

For peer review only

			calculation was performed described in the paragraph 'Study population and setting' in the methods section.
Quantitative variables	11	Explain how quantitative variables were handled in the analyses. If applicable, describe which groupings were chosen and why	Described in 'Data analysis'.
Statistical methods	12	(*a*) Describe all statistical methods, including those used to control for confounding	Described in 'Data analysis'.
		(*b*) Describe any methods used to examine subgroups and interactions	Described in 'Data analysis'.
		(*c*) Explain how missing data were addressed	Not applicable.
		(*d*) *Cohort study*—If applicable, explain how loss to follow-up was addressed<br>*Case-control study*—If applicable, explain how matching of cases and controls was addressed<br>*Cross-sectional study*—If applicable, describe analytical methods taking account of sampling strategy	Not applicable.
		(*e*) Describe any sensitivity analyses	Not applicable.
**Results**			
Participants	13*	(a) Report numbers of individuals at each stage of study—eg numbers potentially eligible, examined for eligibility, confirmed eligible, included in the study, completing follow-up, and analysed	Numbers included during the first round of the survey and the second round of the survey are stated in the first paragraph of the results section. All tables and figures included the number of patients that were analysed.
		(b) Give reasons for non-participation at each stage	Not applicable.
		(c) Consider use of a flow diagram	Not applicable.
Descriptive data	14*	(a) Give characteristics of study participants (eg demographic, clinical, social) and information on exposures and	Characteristics of the

For peer review only

		potential confounders	study population are described in table 1 and table 2.
		(b) Indicate number of participants with missing data for each variable of interest	Not applicable.
		(c) *Cohort study*—Summarise follow-up time (eg, average and total amount)	Not applicable.
Outcome data	15*	*Cohort study*—Report numbers of outcome events or summary measures over time	The numbers of the outcomes are reported in table 3 and table 4 and in the paragraphs 'Parents' response to a sick or injured child' and 'Impact of changes in health services'.
		Case-control study—Report numbers in each exposure category, or summary measures of exposure	Not applicable.
		Cross-sectional study—Report numbers of outcome events or summary measures	Not applicable.
Main results	16	(*a*) Give unadjusted estimates and, if applicable, confounder-adjusted estimates and their precision (eg, 95% confidence interval). Make clear which confounders were adjusted for and why they were included	Absolute numbers and percentages are given. Chi-squared tests were used for comparing groups stratified for children with and without chronic condition.
		(*b*) Report category boundaries when continuous variables were categorized	Described in section 'Data analysis' in methods section.
		(*c*) If relevant, consider translating estimates of relative risk into absolute risk for a meaningful time period	Not applicable.
Other analyses	17	Report other analyses done—eg analyses of subgroups and interactions, and sensitivity analyses	Additional thematic analysis for qualitative data was

For peer review only

			performed and described in 'Data analysis' and in the last paragraph of the results section.
**Discussion**			
Key results	18	Summarise key results with reference to study objectives	Key results are stated in the first paragraph and discussed in the subsequent paragraphs of the discussion section.
Limitations	19	Discuss limitations of the study, taking into account sources of potential bias or imprecision. Discuss both direction and magnitude of any potential bias	Limitations of the study are addressed in the paragraph 'Strengths and limitations' of the discussion section. Any potential bias is discussed as well.
Interpretation	20	Give a cautious overall interpretation of results considering objectives, limitations, multiplicity of analyses, results from similar studies, and other relevant evidence	Interpretation of the results is described in the discussion section.
Generalisability	21	Discuss the generalisability (external validity) of the study results	Generalizability of the results is discussed in the paragraph 'Strengths and limitations'.
**Other information**			
Funding	22	Give the source of funding and the role of the funders for the present study and, if applicable, for the original study on which the present article is based	Funding statement at

For peer review only

			the end of the manuscript.

*Give information separately for cases and controls in case-control studies and, if applicable, for exposed and unexposed groups in cohort and cross-sectional studies.
Note: An Explanation and Elaboration article discusses each checklist item and gives methodological background and published examples of transparent reporting. The STROBE checklist is best used in conjunction with this article (freely available on the Web sites of PLoS Medicine at http://www.plosmedicine.org/, Annals of Internal Medicine at http://www.annals.org/, and Epidemiology at http://www.epidem.com/). Information on the STROBE Initiative is available at www.strobe-statement.org.

For peer review only

BMJ Open

PARENTS' EXPERIENCES WITH A SICK OR INJURED CHILD DURING THE COVID-19 LOCKDOWN: AN ONLINE SURVEY IN THE NETHERLANDS

Journal:	*BMJ Open*
Manuscript ID	bmjopen-2021-055811.R1
Article Type:	Original research
Date Submitted by the Author:	13-Oct-2021

Complete List of Authors:	Tan, Chantal; Erasmus MC Sophia Children Hospital, General Paediatrics<br>Lutgert, Eveline; Erasmus MC Sophia Children Hospital, General Paediatrics<br>Neill, Sarah; University of Plymouth, Faculty of Health<br>Carter, Rachel; University of Plymouth, Faculty of Health<br>Jones, Ray; University of Plymouth, Faculty of Health, Education, and Society<br>Chynoweth, Jade; University of Plymouth, Faculty of Health<br>Borensztajn, Dorine; Erasmus MC Sophia Children Hospital, General Paediatrics<br>Lakhanpaul, Monica; University College London, Institute of Child Health<br>moll, Henriette; Erasmus MC Sophia Children Hospital, Department of Pediatrics

**Primary Subject Heading**:	Paediatrics
Secondary Subject Heading:	Health services research
Keywords:	COVID-19, PAEDIATRICS, Community child health < PAEDIATRICS

SCHOLARONE™
Manuscripts

I, the Submitting Author has the right to grant and does grant on behalf of all authors of the Work (as defined in the below author licence), an exclusive licence and/or a non-exclusive licence for contributions from authors who are: i) UK Crown employees; ii) where BMJ has agreed a CC-BY licence shall apply, and/or iii) in accordance with the terms applicable for US Federal Government officers or employees acting as part of their official duties; on a worldwide, perpetual, irrevocable, royalty-free basis to BMJ Publishing Group Ltd ("BMJ") its licensees and where the relevant Journal is co-owned by BMJ to the co-owners of the Journal, to publish the Work in this journal and any other BMJ products and to exploit all rights, as set out in our licence.

The Submitting Author accepts and understands that any supply made under these terms is made by BMJ to the Submitting Author unless you are acting as an employee on behalf of your employer or a postgraduate student of an affiliated institution which is paying any applicable article publishing charge ("APC") for Open Access articles. Where the Submitting Author wishes to make the Work available on an Open Access basis (and intends to pay the relevant APC), the terms of reuse of such Open Access shall be governed by a Creative Commons licence – details of these licences and which Creative Commons licence will apply to this Work are set out in our licence referred to above.

Other than as permitted in any relevant BMJ Author's Self Archiving Policies, I confirm this Work has not been accepted for publication elsewhere, is not being considered for publication elsewhere and does not duplicate material already published. I confirm all authors consent to publication of this Work and authorise the granting of this licence.

For peer review only

PARENTS' EXPERIENCES WITH A SICK OR INJURED CHILD DURING THE COVID-19 LOCKDOWN: AN ONLINE SURVEY IN THE NETHERLANDS

Chantal D. Tan[1], Eveline K. Lutgert[1], Sarah Neill[2], Rachel Carter[2], Ray Jones[2], Jade Chynoweth[2], Dorine M. Borensztajn[1], Monica Lakhanpaul[3], Henriette A. Moll[1]

Author affiliations:

1. Erasmus MC-Sophia Children's Hospital, Department of General Paediatrics, Rotterdam, the Netherlands
2. Faculty of Health, University of Plymouth, United Kingdom
3. Integrated Community Child Health Population, Policy & Practice Department, UCL Great Ormond Street Institute of Child Health, University College London, United Kingdom

Corresponding author:

Chantal D. Tan, MD
Department of General Paediatrics, Erasmus MC – Sophia Children's Hospital
P.O. Box 2060, 3000 CB, Rotterdam, the Netherlands
Telephone: +31 10 703 72 39
E-mail: c.tan@erasmusmc.nl

Word count: 3994 words

For peer review only

For peer review only - http://bmjopen.bmj.com/site/about/guidelines.xhtml

Abstract

Objective: To assess the impact of the COVID-19 lockdown on parents' help seeking behaviour and care for a sick or injured child in the Netherlands.

Design and setting: An online survey on parents' experiences with a sick or injured child during the COVID-19 lockdown periods was disseminated through social media.

Participants: Parents living in the Netherlands with a sick or injured child during the lockdown periods from March until June 2020 and from December 2020 until February 2021 were eligible to participate.

Outcome measures: Descriptive statistics and thematic analysis were used to analyse family and children's characteristics, parents' response to a sick or injured child, and the perceived impact of the lockdown on child's severity of illness and treatment reported by parents. Analyses were stratified for children with and without chronic conditions.

Results: Of the 105 parents who completed the survey, 83% reported they would have sought medical help before lockdown compared to 88% who did seek help during lockdown for the same specific medical problem. Parents reported that changes in health services affected their child's severity of illness (31%) and their treatment (39%), especially for children with chronic conditions. Negative experiences included long waiting lists, delayed referral and more consultations by telephone. During lockdown, there was no change in help seeking behaviour by parents of children with a chronic condition (N=51) compared to parents of children without a chronic condition.

Conclusion: Parents in the Netherlands who completed the survey were not deterred from seeking medical help for their sick or injured child during the COVID-19 lockdown periods. However, changes in health services affected child's severity of illness and treatment, especially for children with chronic conditions.

For peer review only

Article summary

Strengths and limitations of this study

• Online surveys enable the collection of anonymised data and facilitate the collection of data from a wide range of patients regardless of their residency.

• The survey was disseminated through social media to reach the general population.

• The survey had several 'other' options where parents could give a more detailed explanation of their answers in addition to the multiple-choice answers.

• The advertisement through social media could have caused selection bias as parents who do not have social media or parents with limited (digital) literacy could not have filled in the survey.

• Our study population might not be a good reflection of the general population of ill or injured children as almost 50% of the children had a chronic condition.

For peer review only

For peer review only - http://bmjopen.bmj.com/site/about/guidelines.xhtml

Background

Globally, health care professionals reported that the number of paediatric patients visiting medical services declined significantly while lockdowns were in effect due to the Coronavirus disease 2019 (COVID-19) pandemic: the estimated decline of paediatric visits to Emergency Departments (EDs) during lockdown ranged from 30% in the United Kingdom to 89% in Italy.(1-4) In the Netherlands all medical visits to Dutch EDs declined by 25% during the first lockdown from March 23rd until June 1st 2020.(5) The Dutch Healthcare Authority has reported that paediatric urgent referrals by general practitioners (GPs) declined more than referrals by GPs to other specialties.(6) This decline could partially be explained by a lower incidence of illness or injury in children during lockdown as limited contact with others reduced exposure to infectious diseases and restricted activities reduced accidental injuries.(7, 8) However, other specialties have shown a recuperation of the number of referrals after the first lockdown, while the number of paediatric referrals has not.(6) The Netherlands had two lockdown periods where civilians were strongly advised to mainly stay at home and schools were closed.

Other explanations for the decline in paediatric medical visits are parental concern about COVID-19 and barriers raised by healthcare professionals.(9) On the one hand, there may be positive outcomes from parents' self-care of their children as they develop ways of coping with and managing their child's illness or injury themselves. On the other hand, it is also important to notice the possible negative outcomes of the decrease in parents seeking help for a sick or injured child. In a survey set out by the Dutch Paediatric Society amongst 1400 Dutch paediatricians, there were 51 cases reporting unnecessary collateral harm as a result of delayed presentation to medical health services due to the COVID-19 pandemic. These reports include several cases of diabetic ketoacidosis, sepsis, and cancer.(10) Similar results were found in surveys set out in other European countries.(9, 11, 12) Unfortunately, these surveys do not differentiate between delay caused by parents or by health care professionals.

It is imperative that we try to unravel the reasoning behind the help seeking behaviour of parents for ill or injured children and the impact that the COVID-19 lockdown had on their behaviour. Special attention should be given to parents of children with chronic conditions. They may seek less medical care as they are afraid of COVID-19 infection for their vulnerable child. Concerns were also raised regarding the mental health of children and adolescents during these difficult times. Based on a survey in the United States, 35% of

For peer review only - http://bmjopen.bmj.com/site/about/guidelines.xhtml

adolescents receive mental health support solely in an educational setting.(13) With schools closing, it is possible that certain services could not be accessed properly.(14) Children also ended up spending more time at home and were socially isolated, which may have negatively affected their mental health.(15, 16)

We aimed to assess the impact of the COVID-19 lockdown on parents' experiences with a sick or injured child in the Netherlands, stratified for children with and without a chronic condition. In more detail, we focus on whether and where parents sought help and whether and why their help seeking behaviour had changed due to the lockdown period. In addition, we assessed parents' perceptions of the impact of the lockdown on their child's severity of illness and treatment.

For peer review only

Methods

Study design

This study was a COVID-19 related project in collaboration with the University College London and the University of Plymouth. It was an observational study consisting of an online survey assessing parents' help seeking behaviour for, and care of, a sick or injured child during two COVID-19 lockdown periods. Approval by the ethical committee of the Erasmus MC Sophia was obtained. Informed consent of the participating parents was obtained.

Study population and setting

Parents living in the Netherlands who self-identified as having a sick or injured child during the lockdown periods from March 23rd until June 1st 2020 and from December 15th 2020 until February 16th 2021 were included. The main measures taken during these lockdown periods are shown in chronological order in Appendix A. *(Supplementary file 1)* We excluded parents who did not live in the Netherlands or who did not have a sick or injured child during the afore mentioned lockdown periods. Our aim was to recruit about 100 respondents to our survey. For a sample size of 100, the 95% confidence interval for a 50% estimate of proportions is 40.2% to 59.8% to compare the proportion of parents who sought help before lockdown to the proportion of parents that sought help during the lockdown. For a sample size of 50 in each subgroup of children with and without comorbidity the 95% confidence interval for a 50% estimate proportion is 36.2% to 63.8%.(17, 18)

For peer review only - http://bmjopen.bmj.com/site/about/guidelines.xhtml

Data collection

The original English questionnaire was translated to the Dutch language and some answer options were adapted to the Dutch health care system. *(Supplementary file 2 Appendix B, Supplementary file 3 Appendix C)*. The questionnaire was launched as an online survey created in Google Forms, which enabled the collection of anonymised data. Online surveys facilitate the collection of data from a wide range of parents regardless of their residency. The survey was mainly composed of multiple-choice questions, but 'other' options to add free text were available. This 'other' option also enabled parents to give a more detailed explanation on their previous chosen multiple-choice answer. Respondents were recruited using social media and virtual snowball sampling, in which people helped to disseminate the survey to other parents by reposting the survey on their own social media channels. Information about the survey was posted by the research group on Facebook, Twitter, LinkedIn, Instagram, WhatsApp and professional contacts were sent a request to share the information about the study with their social contacts. Facebook was divided into personal Facebook accounts and Facebook groups, of which the latter included private Facebook groups for parents as well as public Facebook pages of the Erasmus MC and the Sophia Children's Hospital. A short introduction to the survey and a link to the survey was written to share on social media *(Supplementary file 4 Appendix D).* A reminder was posted on social media every two weeks until the end of the survey period. When the number of new respondents stagnated we decided to end the survey period. Key persons who had shared the survey on their social media and their estimated reach were recorded in a document to keep track of the spread of the survey and the number of respondents. There was no incentive provided to parents for participating.

Patient and public involvement

The public was involved in the design and conduct of the study. Parents have been involved in the project as research team members and reviewed the original survey developed in the United Kingdom. In the Netherlands, feedback on the survey and the total duration to fill in the survey came from two parents who were research team members and three parents not working in the medical field. Additionally, feedback on the survey was given by paediatricians and PhD students of the research group of General Paediatrics Erasmus MC Sophia. However, modifications were not needed and these research group members and parents helped disseminating the survey on social media. We plan to disseminate the results to clinicians and health policy makers so that decisions concerning access to health services and support for parents can be made based on evidence.

For peer review only - http://bmjopen.bmj.com/site/about/guidelines.xhtml

Ethical considerations

The study was approved by the Medical Ethics Committee Erasmus Medical Centre (MEC-2020-0627) and by the Scientific Research Committee SARS-CoV-2 and COVID-19 Erasmus Medical Centre. Information about the study and its purpose was explained at the beginning of the online survey. The survey introduction also included a statement about the anonymity of the responses, which explained that no personal identifiable data would be collected. At the end of this information section parents were asked to check a statement, which clearly stated that choosing to complete and submit responses to the survey gave consent to their responses being used in our study. Since we were only including parents in the survey and not the children themselves, there were no concerns about capacity to consent. Information on data usage and dissemination was also provided. Lastly, the respondents had the opportunity to contact the research team if they had additional questions or if they wanted to receive a summary of the study findings. Email addresses were provided for both the project lead and an independent paediatrician to whom any concerns or complaints could be directed.

Data analysis

Data were analysed using SPSS software version 25.0 and free text data was subjected to thematic analysis. Firstly, descriptive statistics were used for the characteristics of parents and families who completed the survey. Information on living area, access to WiFi, children in the family, and whether they have laptops, computers or mobile phones, was collected.

Secondly, descriptive statistics for children's characteristics, stratified for chronic condition, were used. The following question on chronic conditions was included in the survey: "Does your child have any pre-existing illnesses, such as chronic or long term illness, complex needs or a recurring illness?" When parents answered 'yes' they were asked to specify what kind of chronic condition or recurring illness their child had. Children's characteristics, including age, gender, presenting symptoms and hospital admission, were collected as well. Age was categorised into three predefined age groups: <5 years, 5-11 years and 12-17 years. Presenting symptoms were categorised into the following eight subgroups: skin, breathing, body temperature, dehydration, pain, change of behaviour, injury and other.

Thirdly, parents' response to a sick or injured child was described, stratified for chronic condition. The parents were asked what medical symptom(s) their child had during the lockdown and the subsequent questions were 1) whether they would have sought help for the same problem before lockdown and 2) whether they did seek help for this problem during lockdown. Lastly, we analysed whether changes in health services during the lockdown

For peer review only - http://bmjopen.bmj.com/site/about/guidelines.xhtml

affected severity of illness of the child and the child's treatment, stratified for chronic condition. McNemar's test and X^2-tests were used for quantitative analyses with a p-value <0.05 considered as statistically significant. Qualitative analyses using thematic analysis according to Braun and Clarke were used for free text answers from parents on questions regarding the impact of changes in health services on severity of illness of the child and the treatment the child received.(19) Two authors (CT and EL) categorised the free text answers independently to minimise subjectivity of a single researcher's judgement. A selection of quotes from parents regarding the perceived impact of changes in health services were translated (by CT and EL) and divided into three categories: health care services, parents' behaviour and reported health care professionals' behaviour. The quotes were categorized into negative and positive experiences perceived by parents.

Results

A total of 105 respondents filled in the online COVID-19 parent survey in the Netherlands. During the two-month period from October 19th to December 19th 2020, 76 respondents completed the survey regarding the first lockdown period. During the second lockdown, an additional 29 respondents completed the survey from January 16th to February 16th 2021. Figure 1 shows through which social media channel the online survey had reached parents. The online survey reached 60% of the parents via Facebook (personal and groups), 28% via WhatsApp, and 12% via Twitter and LinkedIn.

Parent and family characteristics

The parent and family characteristics are shown in table 1. Most families came from small cities in an urban area (69%) and nearly half were from the province South-Holland (47%). All parents had access to WiFi, nearly all had laptops or computers (95%), mobile phones (90%) and access to outdoor space during lockdown (97%). The majority of the school aged children in the family were staying at home as schools were closed (76%) when the illness or injury happened during lockdown.

For peer review only - http://bmjopen.bmj.com/site/about/guidelines.xhtml

Table 1: Parent and family characteristics (N=105)

	Parent of sick or injured child
**Description of living area**	
Rural – village	6 (6)
Urban - small city	72 (69)
Urban – big city	27 (26)
**Province**	
South Holland	49 (47)
Zeeland	15 (14)
North Brabant	12 (11)
The other 7 provinces	29 (28)
**WiFi access**	105 (100)
**Mobile phone**	95 (90)
**Laptop or computer**	100 (95)
**Access to outdoor space during lockdown** (garden/balcony)	102 (97)
**Where were the school aged children in the family when the illness/injury happened during the lockdown**	
All of the children were staying at home	80 (76)
All the children were attending school	14 (13)
Some of the children were attending school	11 (11)

Absolute numbers and percentages (%) are shown

Children's characteristics

In table 2 the children's characteristics are shown for the total population and stratified for chronic condition. Forty nine percent of the children had a chronic condition or recurring illness (51/105) such as psychomotor retardation/epilepsy, scoliosis, asthma, eczema or recurring otitis media. The majority of the children were below the age of twelve and were boys. The most common presenting symptoms were pain (45%) and change of behaviour (40%). There was a higher percentage of children aged 12-17 years with chronic conditions compared to children without chronic conditions (28% versus 13%). A higher percentage of children with chronic conditions reportedly had breathing problems (29% versus 19%) and a lower percentage had injuries (16% versus 28%) compared to children without chronic conditions. Other presenting symptoms were for example hearing problems, congenital malformations (cleft lip, scoliosis), and facial nerve paralysis. A lower percentage of children

For peer review only - http://bmjopen.bmj.com/site/about/guidelines.xhtml

with chronic conditions were admitted to the hospital during lockdown compared to children without chronic conditions (31% versus 39%).

Table 2: Children's characteristics stratified for chronic condition

	Total (N=105)	Children with chronic condition (N=51)	Children without chronic condition (N=54)
**Age** (years)			
<5	41 (39)	12 (24)	29 (54)
5-11	43 (41)	25 (49)	18 (33)
12-17	21 (20)	14 (28)	7 (13)
**Gender**			
Boys	61 (58)	28 (55)	33 (61)
Girls	44 (42)	23 (45)	21 (39)
**Presenting symptom***			
Skin and appearance	22 (21)	11 (22)	11 (20)
Breathing	25 (24)	15 (29)	10 (19)
Body temperature	16 (15)	9 (18)	7 (13)
Dehydration	21 (20)	11 (22)	10 (19)
Pain	45 (45)	24 (47)	23 (43)
Change of behaviour	42 (40)	21 (41)	21 (39)
Injury	23 (22)	8 (16)	15 (28)
Other	19 (18)	7 (14)	13 (24)
**Hospital admission**			
Admitted	37 (35)	16 (31)	21 (39)

Absolute numbers and percentages (%) are shown

* Possible to have more than 1 presenting symptom

Parents' response to a sick or injured child

Parents reported that 83% (87/105) would have sought help before lockdown, whereas 88% (92/105) of the parents did seek help during lockdown. Help seeking behaviour of 76 parents during the first lockdown and 29 parents during the second lockdown with comparable restrictions did not differ (data not shown). Four parents reported that they would have sought

For peer review only - http://bmjopen.bmj.com/site/about/guidelines.xhtml

help before lockdown but did not seek help during lockdown, whereas nine parents would not have sought help before lockdown but did seek help during lockdown. (McNemar's test, p=0.2) *(Supplementary file 5 Appendix E).* The thirteen parents (13/105, 12%) who decided not to ask for medical help during lockdown stated that they were not sure if their child was ill or injured enough to need medical help, were worried about themselves or a family member catching COVID-19, thought that the advice "to stay at home" meant that they could not go to a health centre or hospital or were worried about using health services when they were needed more urgently by other people. Before the lockdown, parents of children with a chronic condition would have sought help for their sick or injured child in 77% (39/51) compared to 89% (48/54) of the parents of children without a chronic condition (X^2-test, p=0.1). During the lockdown, parents of children with a chronic condition did seek help for their sick or injured child in 82% (42/51) compared to 93% (50/54) of the parents of children without a chronic condition (X^2-test, p=0.1). Help seeking behaviour before and during lockdown related to children's characteristics is shown in Appendix F. *(Supplementary file 6)* Parents of children below the age of five sought help more frequently and the percentage of parents seeking help decreased with increasing child's age. The percentage of parents seeking help for their sick or injured child was comparable for all presenting symptom groups except for the 'other' presenting symptom group.

The reported medical sources used by parents before lockdown were comparable with the reported sources used during lockdown. The majority of the parents contacted the GP and some parents sought help from their child's paediatrician or another medical professional such as the midwife. A small percentage of the parents called the Dutch emergency number or went to the ED. Parents were asked which other actions they undertook for their sick or injured child. Some parents stated that they chose to wait-and-see whether their child got better. When they did decide to treat the illness or injury themselves, they most often treated their child with paracetamol. Parents sought information on how to treat their child on the internet and by asking their social circle of friends and family. When parents stated that information and advice given were useful, this was mainly because they were reassured that what they were doing was right. A quote from a parent was "The information was useful since it reassured me, useful tips and information on when to seek medical help (again) were given". When parents stated that information and advice given were not useful, this was mainly because it was general advice and not child specific. A quote of a parent was "Every child is unique and requires an individual approach".

For peer review only - http://bmjopen.bmj.com/site/about/guidelines.xhtml

Impact of changes in health services

Parents reported that changes in health services during lockdown had impact on the severity of illness of their child in 31% (32/105) and on their child's treatment in 39% (41/105). Parents' explanations were mostly negative and included the following responses: long waiting list for diagnostics or treatment, postponed surgery or out-patient clinic consultation, no assessment by a physician, more consultations by telephone, fewer checks and doctor visits during admission. Lastly some parents stated they waited too long to seek help because they were anxious. Contrarily, there were also some positive responses including shorter turnaround time at the ED and more attention from medical staff during admission as the ward was almost empty. *(Supplementary file 7, Appendix G)* Parents of children with a chronic condition reported that changes in health services affected the severity of their child's illness in 33% (17/51) compared to 28% (15/54) of the parents of children without a chronic condition (X^2-test, p=0.5). Parents of children with a chronic condition reported that changes in health services affected their child's treatment in 47% (24/51) compared to 32% (17/54) of the parents of children without a chronic condition (X^2-test, p=0.1).

Discussion and conclusion

Main findings

Parents in the Netherlands with a sick or injured child during the COVID-19 lockdown and who completed the online survey, were not deterred from seeking medical help for their sick or injured child. Almost 50% of the sick or injured children in our study had a chronic condition or recurring illness. Parents of children without a chronic condition would have sought help more often before lockdown and did seek help more frequently during lockdown than parents of children with a chronic condition. This might be explained by the fact that parents of children with chronic conditions are better able to judge their child's illness or to treat the illness themselves since they have more medical experience. However, more parents of children with a chronic condition reported that changes in health services during lockdown had impact on their child's severity of illness and treatment compared to parents of children without a chronic condition. Parents of children with a chronic condition may have been more anxious during the COVID-19 pandemic about catching COVID-19 themselves or their child, who is perceived to be more vulnerable than a healthy child. The child's age was associated with parents' help seeking behaviour as parents of children below the age of five sought medical help more frequently, both before and during lockdown. This finding is also

For peer review only - http://bmjopen.bmj.com/site/about/guidelines.xhtml

described by Sands et al. where the majority (70%) of medical attendees in children were below the age of five.(20) Remarkably, the number of parents who did seek medical help during the lockdown was high in all presenting symptom groups. This might indicate that parents who completed the survey probably had a more severely ill child. This could also explain the high hospital admission rate (35%) in our study. It has been reported that there is an increase in mental health problems in children due to the lockdown.(21) Where breathing problems are a common presenting symptom to seek medical help, 40% of the children in our study had a change of behaviour as presenting symptom which is typical for the lockdown period.(20)

Strengths and limitations

This online survey was the first national survey in the Netherlands on parents' help seeking behaviour and care for a sick or injured child during the lockdown period due to the COVID-19 pandemic. The survey was circulated through social media to reach the general population, which was also geographically spread throughout the Netherlands. We could not estimate the reach of the survey since it was reposted by many people on social media through virtual snowball sampling. The social media channels of our hospital reached more than 10,000 people, but we could not calculate the response rate as we do not know how many of these children were ill or injured in the lockdown periods. The survey had several 'other' options where parents could give a more detailed explanation of their answers in addition to the multiple-choice answers. There were also some limitations. Firstly, the survey was advertised through social media, which could have caused selection bias since parents who do not have access to social media or who are not active on social media could not have filled in the survey. Parents with limited (digital) literacy would also have been unable to fill in the survey. Additionally, this study design was prone to sampling bias. We did not have data on parents with an injured of sick child who did not fill in the survey. It seemed that parents of children with more serious illness had filled in the survey as this was reflected in the high hospital admission rate of 35%. Therefore generalizability might be limited. The majority of the respondents live in the province South-Holland, which could be explained by the fact that Erasmus MC Sophia is located in South-Holland. This study was prone to information bias and recall bias as well. Parents were asked theoretically whether they would have sought help for the same medical problem before lockdown, which was a potential information bias. Recall bias could have occurred when parents where asked about the sickness/injury of their child during the lockdown periods as these events happened before they filled in the

For peer review only - http://bmjopen.bmj.com/site/about/guidelines.xhtml

questionnaire. However, we assumed that parents who filled in the survey do remember their child's sickness/injury well since these were probably parents of children with more serious illness reflected in the high hospital admission rate. Secondly, our study population might not be a good reflection of the general population of ill or injured children as almost 50% of the children had a chronic condition or recurring illness. This might be explained by the dissemination strategy or parents of children with a chronic condition being more motivated to participate. However, this allowed us to perform analyses stratified for chronic condition which is independent of our sample of parents who had completed the survey. Furthermore, children with a chronic condition are a vulnerable group, who are more prone to serious illness and this study provides important insights in the help seeking behaviour of their parents.

Implications for clinical practice

This study showed that the majority of parents who completed our survey did seek help for a sick or injured child during the lockdown period. However, there were barriers in the help seeking process such as postponed doctors' visits, no physician's assessment and fewer face-to-face consultations were undertaken as more were performed by telephone. A small group of parents decided not to seek help for their sick or injured child because they were not sure if their child was ill or injured enough to need medical help reflecting pre-pandemic research findings (22), were worried about catching COVID-19 or thought that the advice to stay at home meant that they could not attend medical health services. This finding is important since parents should have adequate information on when and where to seek help for a sick or injured child. However, throughout 2020 and 2021, healthcare professionals have been contacting the Dutch media to emphasize the message that EDs and hospitals continue to be available to provide care and treatment for all patients if they need medical help.(23-25)

Conclusion

Parents in the Netherlands who completed the online survey about help seeking behaviour and care for a sick or injured child during the COVID-19 lockdown were not deterred from seeking medical help for their sick or injured child. However, changes in health services did affect their child's severity of illness and treatment, especially for children with a chronic condition. Although the impact was mostly negative there were some positive consequences as well. These findings are important to ensure that we inform parents, especially of children with chronic conditions, on when and where to seek help during lockdown periods to prevent delayed presentation of children with illness or injury to health services.

For peer review only - http://bmjopen.bmj.com/site/about/guidelines.xhtml

References

1. Isba R, Edge R, Jenner R, Broughton E, Francis N, Butler J. Where have all the children gone? Decreases in paediatric emergency department attendances at the start of the COVID-19 pandemic of 2020. Arch Dis Child. 2020;105(7):704.
2. Raucci U, Musolino AM, Di Lallo D, Piga S, Barbieri MA, Pisani M, et al. Impact of the COVID-19 pandemic on the Emergency Department of a tertiary children's hospital. Ital J Pediatr. 2021;47(1):21.
3. Morello F, Bima P, Ferreri E, Chiarlo M, Balzaretti P, Tirabassi G, et al. After the first wave and beyond lockdown: long-lasting changes in emergency department visit number, characteristics, diagnoses, and hospital admissions. Intern Emerg Med. 2021:1-8.
4. Sokoloff WC, Krief WI, Giusto KA, Mohaimin T, Murphy-Hockett C, Rocker J, et al. Pediatric emergency department utilization during the COVID-19 pandemic in New York City. Am J Emerg Med. 2021;45:100-4.
5. Toet H, Sprik, E. & Blatter, B. Effecten van de Corona maatregelen op SEH-bezoeken? Stand van zaken tot en met september 2020. Kenniscentrum letselpreventie; 2020.
6. Zorgautoriteit dN. Analyse van de gevolgen van de coronacrisis voor de reguliere zorg 2020 [updated 29-04-2020. Available from: https://puc.overheid.nl/nza/doc/PUC_306627_22/1/.
7. Hassoun A, Pugh S, Merced I, Sharma M. The power of social isolation on paediatric emergency visits during COVID-19 lockdown. Acta Paediatr. 2020;109(11):2425-6.
8. Keays G, Friedman D, Gagnon I. Injuries in the time of COVID-19 Les blessures au temps de la COVID-19. Health Promot Chronic Dis Prev Can. 2020;40(11-12):336-41.
9. Lazzerini M, Barbi E, Apicella A, Marchetti F, Cardinale F, Trobia G. Delayed access or provision of care in Italy resulting from fear of COVID-19. Lancet Child Adolesc Health. 2020;4(5):e10-e1.
10. Jansen D, Illy KE. Delayed presentation to regular Dutch paediatric care in COVID-19 times: a national survey. BMJ Paediatr Open. 2020;4(1):e000834.
11. Lynn RM, Avis JL, Lenton S, Amin-Chowdhury Z, Ladhani SN. Delayed access to care and late presentations in children during the COVID-19 pandemic: a snapshot survey of 4075 paediatricians in the UK and Ireland. Arch Dis Child. 2021;106(2):e8.
12. Ciacchini B, Tonioli F, Marciano C, Faticato MG, Borali E, Pini Prato A, et al. Reluctance to seek pediatric care during the COVID-19 pandemic and the risks of delayed diagnosis. Ital J Pediatr. 2020;46(1):87.
13. Ali MM, West K, Teich JL, Lynch S, Mutter R, Dubenitz J. Utilization of Mental Health Services in Educational Setting by Adolescents in the United States. J Sch Health. 2019;89(5):393-401.
14. Golberstein E, Wen H, Miller BF. Coronavirus Disease 2019 (COVID-19) and Mental Health for Children and Adolescents. JAMA Pediatr. 2020;174(9):819-20.
15. Rauschenberg C, Schick A, Goetzl C, Roehr S, Riedel-Heller SG, Koppe G, et al. Social isolation, mental health, and use of digital interventions in youth during the COVID-19 pandemic: a nationally representative survey. Eur Psychiatry. 2021:1-32.
16. Chen X, Qi H, Liu R, Feng Y, Li W, Xiang M, et al. Depression, anxiety and associated factors among Chinese adolescents during the COVID-19 outbreak: a comparison of two cross-sectional studies. Transl Psychiatry. 2021;11(1):148.
17. Raosoft. Sample size calculator: Raosoft Inc.; [Available from: http://www.raosoft.com/samplesize.html.
18. Systems CR. Sample Size Calculator: Creative Research Systems; [Available from: https://www.surveysystem.com/sscalc.htm.
19. Braun V, Clarke V. Using thematic analysis in psychology. Qual Res Psychol 2006:3: 77 - 101.

For peer review only - http://bmjopen.bmj.com/site/about/guidelines.xhtml

20. Sands R, Shanmugavadivel D, Stephenson T, Wood D. Medical problems presenting to paediatric emergency departments: 10 years on. Emerg Med J. 2012;29(5):379-82.
21. Newlove-Delgado T, McManus S, Sadler K, Thandi S, Vizard T, Cartwright C, et al. Child mental health in England before and during the COVID-19 lockdown. Lancet Psychiatry. 2021.

22. Neill SJ, Jones CH, Lakhanpaul M, Roland DT, Thompson MJ. Parents' help-seeking behaviours during acute childhood illness at home: A contribution to explanatory theory. J Child Health Care. 2016;20(1):77-86.
23. Willems I. Ziekenhuis St Jansdal slaat alarm over zorgmijders: 'Er staat hier écht geen meneer corona voor de deur': De Stentor; 2021 [updated 19-02-2021. Available from: https://www.destentor.nl/veluwe/ziekenhuis-st-jansdal-slaat-alarm-over-zorgmijders-er-staat-hier-echt-geen-meneer-corona-voor-de-deur-br~a76a3e50/?referrer=https%3A%2F%2Fwww.google.com%2F.
24. Sevil M. Huisartsen: kom met serieuze klachten wel langs: Het Parool; 2020 [updated 30-03-2020. Available from: https://www.parool.nl/nieuws/huisartsen-kom-met-serieuze-klachten-wel-langs~b8e4495b/.
25. NOS. Zorgen op spoedeisende hulp om wegblijvende patiënten: 'Kom vooral wel': NOS; 2020 [updated 27-03-2020. Available from: https://nos.nl/artikel/2328538-zorgen-op-spoedeisende-hulp-om-wegblijvende-patienten-kom-vooral-wel.html.

For peer review only

For peer review only - http://bmjopen.bmj.com/site/about/guidelines.xhtml

Text for figure 1:

Figure 1: Pie chart demonstrating through which social media channel the online survey had reached parents

Authors contributions

Chantal Tan and Eveline Lutgert contributed to the conceptualisation and design of the study, coordinated the data collection, carried out the analyses, and drafted the initial and final manuscript. Dorine Borensztajn, Sarah Neill, Rachel Carter, Ray Jones, Jade Chynoweth and Monica Lakhanpaul contributed to the conceptualisation and design of the study, supervised the analyses and critically reviewed the manuscript. Henriette Moll contributed to conceptualisation and design of the study, contributed to interpretation of the data, critically reviewed the manuscript and supervised the study. All authors approved the final manuscript as submitted and agree to be accountable for all aspects of the work.

Funding statement:

This project has received funding from the European Union's Horizon 2020 research and innovation programme under grant agreement No. 848196. The funding sources had no role in the design and conduct of the study; collection, management, analysis, and interpretation of the data; preparation, review, or approval of the manuscript; and decision to submit the manuscript for publication.

Competing interests statement:

There are no competing interests.

Patient consent for publication:

Not required.

Ethics approval:

The study was approved by the Medical Ethics Committee Erasmus Medical Centre (MEC-2020-0627) and by the Scientific Research Committee SARS-CoV-2 and COVID-19 Erasmus Medical Centre.

For peer review only - http://bmjopen.bmj.com/site/about/guidelines.xhtml

For peer review only

Data availability statement:

Data are available upon request. Data from this study are available upon request to the corresponding author of the study (c.tan@erasmusmc.nl), subject to local rules and regulations.

For peer review only

For peer review only - http://bmjopen.bmj.com/site/about/guidelines.xhtml

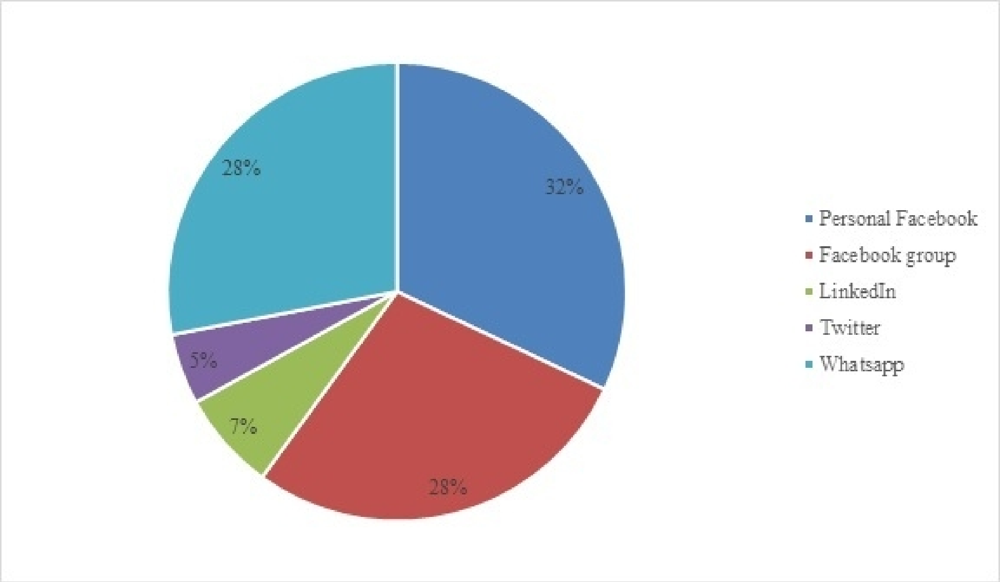

Figure 1: Pie chart demonstrating through which social media channel the online survey had reached parents

48x28mm (300 x 300 DPI)

SUPPLEMENTARY FILE 1

Appendix A: Timeline of measures taken during the first and second lockdown period

**Date**	**Measures**
	First lockdown March 23rd – June 1st 2020
March 23rd	- > 2 persons in public area prohibited with the exception of one household<br>- Group formation only with 1.5 meter distancing rule, maximum 100 persons<br>- Closing of catering industries and schools<br>- Contact professions prohibited<br>- Stores open with 1.5 meter distancing rule and hygiene measures<br>- Households can have maximum of 3 persons visiting them with 1.5 meter distancing rule<br>- Work from home as much as possible<br>- In case of violation 390 euro fine, companies 4350 euro fine
May 11th	- Children day-care and primary schools reopening (in smaller groups)<br>- Contact professions reopening<br>- Playing sports outside allowed with 1.5 meter distance<br>- Opening libraries
June 1st	- Face mask mandatory in public transport, only use public transport when necessary<br>- Restaurants / catering industries reopening for max 30 persons, booking in advance<br>- Museums, cinemas reopening for max 100 persons<br>- High school reopening
June 8th	- Primary school fully reopened
August 6th	- Announcement of the extension of a flight restriction to European countries, the United Kingdom, all countries on the Western hemisphere.
October 14th	- Closing of catering industries

For peer review only

	- A ban on selling alcohol between 8 pm and 7 am<br>- Maximum of 3 visitors per home, maximum of 30 people in interior spaces, outside no more than 4 people from other households.<br>- A ban on late night shopping
December 1st	- Facemasks become mandatory in public places, in schools, in public transport and for contact professions
	Second lockdown December 15th 2020 - February 16th 2021
December 15th	- Closing of non-essential shops<br>- Closing of theatres, cinemas, museums, zoo's, sauna's and libraries<br>- Contact professions closing (not including physiotherapists)<br>- Closing of restaurants and schools (schools starting from December 16th)<br>- Households can have maximal 2 persons visiting them with 1.5 meter distancing rule (on Christmas a maximum of 3 persons)<br>- Work from home as much as possible
December 29th	- Travellers to the Netherlands need to provide a negative PCR-test (not older than 72 hours)
January 6th	- Start of the vaccination program (healthcare workers from the emergency medicine)
January 23rd	- Mandatory curfew from 09 pm until 04.30 am
February 8th	Primary school and day-care reopening

For peer review only

SUPPLEMENTARY FILE 2

Appendix B: Original English version of the online survey

Thank you for helping us with our survey. We would like to find out what impact the Stay Home period is having on parent's actions and decision when caring for, or seeking help for, a sick or injured child. The Stay Home period is sometimes called the lockdown that started on the 23rd March when the government put restrictions on movements to stop the spread of COVID-19. The findings will help us to learn from parents' experiences and direct the development of support for parents with a sick or injured child in the future.

If your child has been ill or injured while you have been following government advice to Stay Home, we would be most grateful if you could complete our survey. We would like to know about your experiences of caring for a sick or injured child at this time, whether or not you decided to seek medical help. We would like parents to complete the survey whether or not their child needed hospital care.

If you do kindly complete and submit the survey this will indicate that you are happy for us to use your responses in our project. Your responses are, of course, anonymous so it will not be possible to identify your replies to remove them after you have completed the survey. Your survey responses will go directly to the survey team.

We will write up the findings from the project and share these in academic journals, in communication with health service leaders and policy makers, at conferences, in education, in future research projects and online. If you provide any free text comments your words may be used but it will not be possible to identify you. If you would like a summary of our findings please contact one of the project leads.

The leads for this project are Professor Sarah Neill sarah.neill@plymouth.ac.uk and Rachel Carter r.carter@plymouth.ac.uk

I can confirm that I have read the information above, I am happy to complete the questionnaire and I am happy for the information I give to be used for the project. Tick box

Are you a UK resident with a child who has been ill or injured since you were asked to Stay Home?

Yes – directed to the questionnaire

No - we thank you for your interest but we are only looking for parents living in the UK whose children have been ill or injured whilst being asked to Stay Home. We hope that you and your family stay well during this period.

For peer review only

It may be that your child has had a number of illnesses or injuries in the last few weeks since you were asked to stay at home. We would like you to select the most serious of these and answer the remaining questions with that in mind.

Question 1 About the child who was ill or injured

a. How old is your child?
   - Under 12 months old
   - 12 months and under 24 months old
   - 2 years and under 5 years old
   - 5 years and under 12 years old
   - 12 years and under 16 years old
   - 16 and under 18 years old

b. Was your child born a girl or boy?
   Girl
   Boy

c. Does your child have any pre-existing illnesses, such as chronic or long term illness, complex needs or a recurring illness?

If yes, please tell us what they are:

Question 2 What signs or symptoms of illness or injury did your child have? Please tick all that apply

Skin and appearance

- Skin very pale or blue, or the inside of their lips and tongue are blue
- Skin appeared mottled
- Rash which disappeared when pressed
- Rash which did not disappear with pressure (the 'Glass test')

Breathing difficulties

- Breathing stopped for a long time (more than 10 seconds at a time),
- Breathing paused (apnoeic episodes) for 5-10 seconds
- Struggling to breathe
- Sucking in muscles under their ribs, between the ribs or at the neck with each breath
- Grunting with the effort of trying to breathe
- Breathing faster than normal
- Noisy breathing (wheeze or a stridor)

Body temperature

- Under 3 months of age with a temperature of over 38°C/100.4^{o}F
- 3 and 6 months of age with a temperature over 39°C/102.2^{o}F

For peer review only

- Over 6 months old with a temperature above 38°C/100.4°F for more than 5 days
- Under 1 month of age and had a low temperature below 36°C/96.8°F

Dehydration (seemed to be too dry)

- Dry mouth
- Sunken eyes
- No tears
- Drowsy
- Passing less urine than usual
- Passing more urine than usual
- Drinking more than normal

Pain

- Tummy pain
- Back pain
- Painful testicles
- Headache
- Persistent pain in an injured arm or leg
- Earache
- Sore throat

Change in behaviour

- Agitated
- Persistent crying/inconsolable
- Fit/seizure
- Confused
- Wouldn't wake up or, if woken up, they wouldn't stay awake
- Unresponsive

Injury

- Burn
- Head injury
- Leg or arm injury
- Fall
- Cut(s)
- Bleeding
- Limping
- Difficulty in moving a part of the body

Other, please give details

For peer review only

Question 3 Before the advice to Stay Home, what would your *usual* response have been to this illness or injury? (Please tick one only)

- Care for my child at home
- Ask for medical help
- Something else, please give details

If you would *normally* have asked for medical help please go to Question 4

If you would *not normally* have asked for medical help please go to Question 5

If you answered *something else*, please go to Question 5

Question 4 Where would you *normally* have asked for medical help for your child with this illness or injury <u>in the first instance</u>? (Choose one only)

- NHS Direct 111
- GP surgery
- Via my GP's website
- Video consultation
- Walk in centre
- Urgent care centre
- Minor injuries unit
- GP Out of Hours service
- Calling 999
- Accident and Emergency
- Somewhere else, please say where……

Question 5 Did you ask for medical help for your child during this most recent illness or injury?

Yes, please continue to Question 6

No, please go to Question 9

Question 6 Where did you ask for medical help during this most recent illness or injury? (Please tick all that apply)

- NHS Direct 111
- GP surgery
- Via my GP's website

For peer review only

- Video consultation
- Walk in centre
- Urgent care centre
- Minor injuries unit
- GP Out of Hours service
- Called 999
- Accident and Emergency
- Somewhere else, please say where……

Question 7 Was your child admitted to hospital for this most recent illness or injury? Yes/No

If yes go to question 10

If no, continue to the next question

Question 8 What, if anything else, would you like to tell us about your experience, for example, whether advice was helpful or not helpful.

Go to Question 10 for all respondents

Question 9 Why did you decide not to ask for medical help? Please tick all that apply

- I was not sure if my child was ill or injured enough to need medical help
- I was worried about myself or a member of my family catching COVID19
- I was worried that I might be criticised for using the service if it wasn't an emergency
- I thought the Stay Home advice meant I couldn't go to a health centre or a hospital
- I was worried about using a service when it is needed more urgently by other people
- I was worried that the service would be extremely busy and that I would have to wait for too long
- I don't have a car and didn't want to use public transport
- I don't have anyone to look after my other children
- Something else - please give details

Question 10 What else did you do about your child's illness or injury? Please tick all that apply

a. I waited to see whether or not my child got better
b. I treated my child's illness/injury myself with:
    - paracetamol (Calpol)
    - ibuprofen (Brufen/Junifen)

For peer review only

- a prescribed inhaler
- Vicks or Karvol
- A cold compress/ice pack
- Cleansing
- Bandaging
- A sling to rest an injured arm
- Elevated an injured limb
- Cold drinks
- Warm drinks
- Cool bath
- Warm bath
- Treats and attention
- Distracting my child
- Home remedies, please explain what these were
- Other, please tell us what this was

c. I looked for information on how to manage the illness or injury at home from:

- family
- friends
- a family health professional
- books
- TV
- magazines
- newspapers
- radio
- the internet
- an NHS app
- Other app
- Social media (such as Twitter, Facebook, Instagram, YouTube)
- Google
- NHS Choices
- GP website
- Other website
- Other: please state

d. I used information I already had from (tick all that apply):

- family
- friends
- a family health professional
- books

For peer review only

- TV
- Magazines
- Newspapers
- radio
- an NHS app
- Other app
- Social media (such as Twitter, Facebook, Instagram, YouTube)
- Google
- NHS Choices
- GP website
- Other website
- School or college course
- A mixture of these resources
- Other: please state

Question 11 Helpfulness of information

a. What, if any, information was helpful and if so why?

b. What, if any, information was unhelpful and if so why?

Question 12 Advice provided during the Stay Home period

a. Which of the following sources of advice have you seen about when to use health services while families are being told to Stay Home? Please tick all that apply

- Government sources (such as Government TV or radio broadcast, the letter from Boris Johnson, daily briefings from Government)
- NHS sources (such as NHS Choices website, GP website, 111 service announcements)
- Other expert online sources (such as University, research organisations etc)
- Experts on Social Media sites (such as Twitter, Facebook)
  - Family/Friends on Social Media sites
  - Other, please give details

b. Have you seen advice about when to (Please tick all that apply)
   - call NHS111?
   - call your GP/family doctor?
   - attend accident and emergency with your children?

c. What, if anything, was new about advice on health service use since you have been told to Stay Home?

For peer review only

Question 13 Have the changes to health services during the Stay Home period affected how ill your child has been? Yes, Maybe, No

d. If yes, please tell us how.

Question 14 Have the changes to health services during the Stay Home period affected any treatment your child received? Yes, Maybe, No, Not applicable

e. If yes, please us how.

Please tell us about your family

Question 15 How old are the children in the household?

**Age of children**	**Please tell us the number of children in each age group**
0-4 years	
5-11 years	
12-15 years	
16-18 years	

Question 16 What area do you live in?

- Scotland
- Wales
- Northern Ireland
- East of England
- London
- Midlands
- North East and Yorkshire
- North West
- South East
- South West

Question 17 Which of the following best describes the area in which you live?

- Rural
- Urban

Questions 18 What access do you have to technology: Please tick all that apply

For peer review only

a. What type of mobile phone do you have?
   - for calls and texting only
   - Smartphone

b. What type of mobile phone contract do you have?
   - Pay as you go mobile phone
   - Monthly contract mobile phone

c. What type of computer do you have?
   - laptop
   - desktop computer

d. How much WiFi access do you have?
   - Unlimited WiFi access
   - Limited WiFi access
   - Other access to the internet, please state

Question 19 Do you have access to outdoor space? Yes/No

Question 20 We know that some children are still able to attend school. Where were the school aged children in your family when the illness or injury happened?

- All of my children were attending school
- Some of my children were attending school
- All of my children were staying at home

Question 21 What if, anything else, would you like to tell the project team?

Thank you for taking the time to complete our questionnaire.

If you would like to receive a summary of the project findings please email one of the project leads:

Professor Sarah Neill *sarah.neill@plymouth.ac.uk* or Rachel Carter *r.carter@plymouth.ac.uk*

If you have any concerns or complaints about the project please contact the Research Administrator to the Faculty Research Ethics and Integrity Committee (*hhsethics@plymouth.ac.uk*)

For peer review only

SUPPLEMENTARY FILE 3

Appendix C: Adapted Dutch version of the online survey

Bedankt voor uw hulp bij onze enquête. Wij willen graag weten wat voor gevolgen de "lockdown periode" heeft gehad op de acties en beslissingen van ouders bij de zorg of het zoeken van hulp voor een ziek of gewond kind. Onze "intelligente lockdown" ging in op 23 maart 2020 toen de overheid beperkingen oplegde om zo de verspreiding van COVID-19 te stoppen. De resultaten zullen ons helpen om te leren van de ervaringen van ouders en de ondersteuning voor ouders met een ziek of gewond kind in de toekomst te verbeteren.

Indien uw kind ziek of gewond was terwijl u het advies van de overheid opvolgde om zoveel mogelijk thuis te blijven gedurende de intelligente lockdown periode van 23 maart tot 1 juni 2020, zouden we u zeer dankbaar zijn als u onze enquête wilt invullen. We willen weten wat uw ervaringen zijn met de zorg voor een ziek of gewond kind gedurende deze periode en of u besloot om wel of niet medische hulp te zoeken. We willen graag weten of uw kind een vorm van gezondheidszorg (zoals het raadplegen van de huisarts, de huisartsenpost of ziekenhuiszorg) nodig had.

Indien u de enquête volledig invult en indient, geeft u toestemming aan ons om uw antwoorden voor ons onderzoek te gebruiken. Uw antwoorden zijn volledig anoniem en niet naar u of uw kind te herleiden. Uw antwoorden gaan rechtstreeks naar het onderzoeksteam.

De enquête bestaat uit ongeveer 20 vragen en het invullen hiervan zal maximaal 10 minuten duren.

We zullen de resultaten van het onderzoek delen in wetenschappelijke tijdschriften en websites, in communicatie met artsen en beleidsmakers, op congressen, in onderwijs en in toekomstige onderzoeksprojecten. Als u opmerkingen in de vrije tekst invult, kunnen uw woorden worden gebruikt, maar is het niet mogelijk u te identificeren. Indien u een samenvatting van onze resultaten wilt, neem dan contact op met een van de projectleiders.

Dit project is een samenwerking tussen het Erasmus MC Sophia Kinderziekenhuis in Nederland en University of Plymouth en University College London in Engeland. Deze studie is goedgekeurd door de Commissie Wetenschappelijk Onderzoek COVID-19 van het Erasmus Medisch Centrum te Rotterdam (CWO COVID-19 EMC).

De projectleiders zijn professor Henriette Moll, kinderarts Dorine Borensztajn en arts-onderzoeker Chantal Tan.

Indien u nog vragen heeft over het onderzoek, kunt u contact opnemen met de projectleiders door een mail te sturen naar *ervaringen.ouders@erasmusmc.nl*.

For peer review only

Introductie vragen over deelname aan enquête en manier van werving

1) Ik kan bevestigen dat ik bovenstaande informatie heb gelezen en ik wil graag de enquête invullen en informatie geven voor het onderzoek.

- Ja
- Nee

2) Bent u een inwoner van Nederland en was uw kind ziek of gewond kind terwijl u het advies van de overheid opvolgde om zoveel mogelijk thuis te blijven gedurende de lockdown periode?

- Ja – u wordt doorverwezen naar de vragenlijst
- Nee – we danken u voor uw interesse, maar we zijn alleen op zoek naar ouders die in Nederland wonen en wiens kind(eren) ziek of gewond was tijdens de lockdown periode.

3). Hoe bent u bij deze vragenlijst terecht gekomen?

- Ik zag een bericht met de link naar dit formulier in een Facebook groep voor ouders/moeders
- Ik zag een bericht met de link naar dit formulier via-via op Facebook
- Ik zag een bericht met de link naar dit formulier op LinkedIn
- Ik zag een bericht met de link naar dit formulier op Twitter
- Ik kreeg een bericht met de link naar dit formulier via WhatsApp toegestuurd

1. Vragen over het zieke of gewonde kind

Het kan zijn dat uw kind meerdere ziekte episodes of verwondingen heeft gehad tijdens de "lockdown periode". We willen graag dat u de meest ernstige hiervan selecteert en de volgende vragen met die ziekte of verwonding in gedachte beantwoordt.

a). Hoe oud was uw kind ten tijde van de ziekte of verwonding?

- Jonger dan 12 maanden oud
- 12 maanden of ouders maar jonger dan 24 maanden oud
- 2 jaar of ouders maar jonger dan 5 jaar oud
- 5 jaar of ouder maar jonger dan 12 jaar oud
- 12 jaar of ouders maar jonger dan 16 jaar oud
- 16 of 17 jaar oud

b). Is uw kind een jongen of een meisje?

- Meisje
- Jongen

c). Is uw kind bekend met reeds bestaande ziekten, zoals chronische of langdurige ziekten, complexe behoeften of terugkerende ziekten?

Indien ja, graag toelichting welke ziekte(n) dit zijn:

For peer review only

2. Welke kenmerken of symptomen van ziekte of verwonding had uw kind.
Vink alles aan wat van toepassing is

- Huid en uiterlijk
  - Huid erg bleek of blauw, of binnenkant van de lippen en tong blauw
  - Vlekkerige huid
  - Uitslag die verdween wanneer erop werd gedrukt
  - Uitslag die niet verdween wanneer erop werd gedrukt
- Ademhalingsproblemen
  - Ademhaling stopte voor een lange periode (>10 seconden per keer)
  - Ademhaling onderbroken (ademstops) voor 5-10 seconden
  - Moeite hebben met ademen
  - Intrekkingen in de hals, en/of onder of tussen de ribben bij elke ademhaling
  - Kreunen bij de ademhaling
  - Sneller ademen dan normaal
  - Hoorbare ademhaling (gierende of piepende ademhaling)
- Lichaamstemperatuur
  - Jonger dan 3 maanden oud met een temperatuur boven de 38oC
  - Tussen de 3 en 6 maanden oud met een temperatuur boven de 39oC
  - Ouder dan 6 maanden oud met een temperatuur boven de 38oC voor langer dan 5 dagen
  - Jonger dan 1 maand oud met een temperatuur onder de 36oC
- Uitdroging
  - Droge mond
  - Ingevallen ogen
  - Geen tranen
  - Suf
  - Minder plassen dan normaal
  - Meer plassen dan normaal
  - Minder drinken dan normaal
  - Meer drinken dan normaal
- Pijn
  - Buikpijn
  - Rugpijn
  - Pijnlijke testikels
  - Hoofdpijn
  - Persisterende pijn in een gewond arm of been
  - Oorpijn
  - Keelpijn

For peer review only

- Verandering in gedrag
  - Geagiteerd (sneller boos of geïrriteerd zijn)
  - Aanhoudend huilen/ontroostbaar
  - Toeval of epileptische aanval
  - Verward
  - Niet wakker worden of niet wakker kunnen blijven
  - Niet reagerend
- Verwonding
  - Verbranding
  - Hoofdverwonding
  - Verwonding aan been of arm
  - Val
  - Snijwond
  - Bloeding
  - Mank lopen
  - Moeite om een lichaamsdeel te bewegen
- Andere ziekte of verwonding

Mocht de ziekte en/of verwonding van uw kind niet hierboven vermeld staan, graag hieronder uw toelichting

3. Wat zou uw gebruikelijke reactie zijn op deze ziekte of verwonding zijn geweest vóór de intelligente lockdown periode?

- Om medische hulp vragen → ga naar vraag 4
- Thuis voor mijn kind zorgen → ga naar vraag 5
- Anders → ga naar vraag 5

4. Waar zou u normaliter allereerst om medische hulp hebben gevraagd voor uw kind bij deze ziekte of verwonding?

- Eigen waarnemend huisarts (overdag)
- Telefonisch consult met de huisarts
- Inloop spreekuur bij de huisarts
- Website eigen huisarts
- Website thuisarts.nl
- Huisartsenpost (in de avond/nacht)
- Behandelend kinderarts, waar mijn kind onder controle is
- 112 bellen
- Spoedeisende hulp
- Anders

For peer review only

5. Heeft u om medische hulp gevraagd voor de ziekte of verwonding van uw kind tijdens de lockdown?

- Ja → ga naar vraag 6
- Nee → ga naar vraag 9

6. Waar heeft u om medische hulp gevraagd voor deze ziekte of verwonding tijdens de lockdown?

Vink alles aan wat van toepassing is

- Eigen waarnemend huisarts (overdag)
- Telefonisch consult met de huisarts
- Inloop spreekuur bij de huisarts
- Website eigen huisarts
- Website thuisarts.nl
- Huisartsenpost (in de avond/nacht)
- Behandelend kinderarts, waar mijn kind onder controle is
- 112 bellen
- Spoedeisende hulp
- Anders

7. Was uw kind opgenomen in het ziekenhuis vanwege de ziekte of verwonding tijdens de lockdown?

8. Wat zou u nog willen vertellen over uw ervaring? Bijvoorbeeld of het advies wat u heeft gekregen nuttig was of niet. → ga naar vraag 10

9. Waarom had u besloten om geen medische hulp te vragen? Vink alles aan wat van toepassing is

- Ik wist niet zeker of mijn kind ziek of gewond genoeg was om medische hulp te zoeken
- Ik was bezorgd dat ikzelf, mijn kind of een familielid COVID-19 zou krijgen
- Ik was bezorgd dat ik kritiek zou krijgen als ik medische zorg zou gebruiken als het geen noodgeval was
- Ik dacht dat het advies om zoveel mogelijk thuis te blijven tijdens de lockdown periode betekende dat ik niet naar een gezondheidsinstelling of ziekenhuis kon gaan
- Ik maakte me zorgen over het gebruik van medische zorg wanneer deze voor andere mensen meer nodig was
- Ik was bang dat de gezondheidsinstelling (huisarts of ziekenhuis) extreem druk zou zijn en dat ik lang zou moeten wachten
- Ik heb geen auto en ik wilde geen gebruik maken van het openbaar vervoer
- Ik heb niemand die op mijn andere kinderen kan passen

For peer review only

- Anders

10. Wat heeft u nog meer gedaan aan de ziekte of verwonding van uw kind?

- Ik heb gewacht om aan te kijken of mijn kind al dan niet beter werd
- Ik heb de ziekte of verwonding van mijn kind zelf behandeld. Vink alles aan wat van toepassing is
    - Paracetamol
    - Ibuprofen
    - Een voorgeschreven puffer
    - Vicks
    - Een koude kompres/koeling met ijs
    - Schoonmaken van de wond
    - Verbinden
    - Een draagdoek om een gewond arm te laten rusten
    - Een gewond ledemaat ondersteunen door het hoog te houden
    - Koude dranken
    - Warme dranken
    - Koud bad
    - Warm bad
    - Traktaties en aandacht
    - Mijn kind afleiden
    - Huismiddeltjes, graag uw toelichting
    - Anders
- Ik heb informatie gezocht over hoe ik thuis de ziekte of verwonding kon behandelen**.** Kunt u toelichten waar of via wie u deze informatie heeft opgezocht?
    - Familie
    - Vrienden
    - Een vriend of familielid die zorgmedewerker is
    - Boeken
    - TV
    - Tijdschriften
    - Kranten
    - Radio
    - Het internet
    - App: moet ik naar de dokter?
    - Andere apps
    - Sociale media (zoals Twitter, Facebook, Instagram, Youtube)
    - Google
    - Thuisarts.nl
    - Een andere website

For peer review only

- Anders
- Ik heb informatie gebruikt welke ik al had. Kunt u toelichten waarvan of van wie u deze informatie al had gekregen?
  - Familie
  - Vrienden
  - Een vriend of familielid die zorgmedewerker is
  - Boeken
  - TV
  - Tijdschriften
  - Kranten
  - Radio
  - Het internet
  - App: moet ik naar de dokter?
  - Andere apps
  - Sociale media (zoals Twitter, Facebook, Instagram, Youtube)
  - Google
  - Thuisarts.nl
  - Een andere website
  - Anders
  -
- Niet van toepassing

11. Als de gevonden informatie nuttig was om u te helpen met de ziekte of verwonding van uw kind, wat vond u hier nuttig aan en waarom?
Als de gevonden informatie niet nuttig was, wat vond u hier niet nuttig aan en waarom?

12. Welke van de volgende bronnen voor advies bent u tegen gekomen wanneer u medische zorg nodig had gedurende de lockdown. Vink alles aan wat van toepassing is.

- Overheidsbronnen (dagelijks nieuws van overheid/RIVM)
- Gezondheidszorg bronnen (zoals website van uw eigen huisarts, thuisarts.nl, app moet ik naar de dokter?)
- Andere online expert bronnen (zoals universitaire of onderzoeksorganisaties etc)
- Deskundigen op sociale media (zoals Twitter, Facebook)
- Familie/vrienden op sociale media
- Anders

Heeft u advies gevonden in deze bron(nen) over wanneer u…

- ... de huisartsenpost moest bellen?
- … uw eigen huisarts moet bellen?

For peer review only

- … naar de spoedeisende hulp moet komen met uw kind?

Wat was nieuw aan het advies wat u heeft gezien omtrent het gebruik van medische zorg gedurende de lockdown?

13. Hebben veranderingen in de medische zorg gedurende de lockdown invloed gehad op hoe ziek uw kind was?

- Ja → graag uw toelichting
- Nee

14. Hebben veranderingen in medische zorg gedurende de lockdown invloed gehad op de behandeling die uw kind heeft gekregen?

- Ja → graag uw toelichting
- Nee
- Misschien
- Niet van toepassing

15. Hoe oud waren de kinderen in uw huishouden gedurende de lockdown periode? Vul het aantal kinderen per leeftijdsgroep in

- Leeftijd van de kinderen 0-4 jaar
- Leeftijd van de kinderen 5-11 jaar
- Leeftijd van de kinderen 12-15 jaar
- Leeftijd van de kinderen 16-17 jaar

16. In welke provincie woont u?

- Drenthe
- Flevoland
- Friesland
- Gelderland
- Groningen
- Limburg
- Noord-Brabant
- Noord-Holland
- Overijssel
- Utrecht
- Zeeland
- Zuid-Holland

Welke van de volgende opties omschrijft uw woonomgeving het beste?

For peer review only

- Grote stad
- Kleine stad/dorp
- Platteland

Had u de mogelijkheid om tijdens de lockdown buiten in uw tuin of op uw balkon te zijn?

- Ja
- Nee

17. Welke toegang heeft u tot technologie?

Wat voor mobiele telefoon heeft u ?

- Prepaid mobiele telefoon
- Maand abonnement
- Niet van toepassing

Wat voor soort computer heeft u?

- Een laptop
- Computer
- Ik heb geen computer

In welke mate heeft u toegang tot WiFi?

- Onbeperkte WiFi toegang
- Beperkte WiFi toegang
- Geen WiFi toegang
- Anders

18. We weten dan sommige kinderen nog naar school konden gaan. Waar waren de schoolgaande kinderen in uw gezin wanneer ze ziek of gewond waren gedurende de lockdown?

- Al mijn kinderen gingen naar school
- Sommige van mijn kinderen gingen naar school
- Al mijn kinderen bleven thuis

19. Heeft u nog opmerkingen voor het onderzoeksteam?

Hartelijk dank voor uw tijd en interesse om deze vragenlijst te beantwoorden.

For peer review only

Indien u een samenvatting van de bevindingen van het onderzoek zou willen ontvangen, stuur dan een mail naar de projectleider: Professor Henriette Moll (*ervaringen.ouders@erasmusmc.nl*)

Indien u enige zorgen of klachten heeft over het onderzoek, neem dan graag contact op met een onafhankelijk kinderarts (*secretariaatalkg@erasmusmc.nl*)

For peer review only

SUPPLEMENTARY FILE 4

Appendix D: Introduction of the survey on social media

Advertising text:

De coronaperiode is een zware dobber voor ouders. Is uw kind ziek of gewond geweest in de lockdown periode en wilt u bijdragen aan onderzoek van het Sophia Kinderziekenhuis, vul dan deze online enquête anoniem in (maximale duur 10 minuten):

h*ttps://forms.gle/GNyiP7G2xKnoSR9j8*

Alvast bedankt voor uw medewerking. Delen van dit bericht wordt zeer gewaardeerd!

Translation of advertising text:

The corona period is a tough one for parents. If your child has been ill or injured during the lockdown period and you want to contribute to research of the Sophia Children's Hospital, please complete this online survey anonymously (maximum duration 10 minutes):

https://forms.gle /GNyiP7G2xKnoSR9j8

Thank you in advance for your cooperation. Sharing this post is much appreciated!

Advertising image:

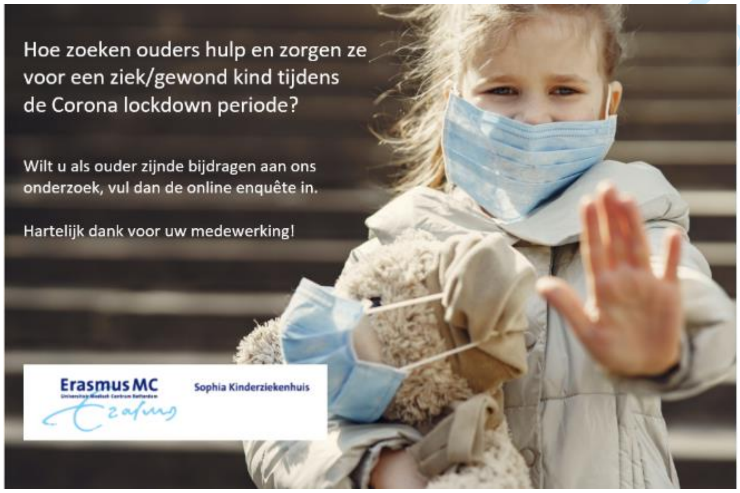

*The image was obtained from the following webpage and was freely available to use: https://www.pexels.com/photo/serious-girl-in-protective-mask-holding-plush-toy-in-mask-and-showing-palm-against-steps-4000605/

For peer review only

Translation of text on advertising image:

How do parents seek help and care for a sick or injured child during the corona lockdown period? Do you as a parent want to contribute to research, please fill in this online survey. Thank you very much for your cooperation!

For peer review only

SUPPLEMENTARY FILE 5

Appendix E: Table for McNemar's test

		Did seek help during lockdown		
**Would have sought help before lockdown**		*Yes*	*No*	*Total children*
	Yes	*83*	*4*	*87*
	No	*9*	*9*	*18*
	Total children	*92*	*13*	*105*

McNemar's test p=0.2

For peer review only

SUPPLEMENTARY FILE 6

Appendix F: Parents' help seeking behaviour related to children's characteristics

	**Sick/injured children (N=105)**	**Would have sought help before lockdown ¥**	**Did seek help during lockdown ¥**
**Age** (years)			
<5	41 (39)	37 (90)	39 (95)
5-11	43 (41)	33 (76)	36 (84)
12-17	21(20)	17(81)	17 (81)
**Gender**			
Boys	61 (58)	49 (80)	52 (85)
Girls	44 (42)	38 (86)	40 (91)
**Presenting symptom***			
Skin	22 (21)	18 (82)	21 (95)
Breathing	25 (24)	21 (84)	24 (96)
Body temperature	16 (15)	16 (100)	16 (100)
Dehydration	21 (20)	18 (86)	20 (95)
Pain	45 (45)	40 (89)	44 (94)
Change of behaviour	42 (40)	32 (76)	38 (91)
Injury	23 (22)	19(83)	23 (100)
Other	19 (18)	17 (89)	15 (75)
**Hospital admission**	37 (35)	36 (97)	37 (100)

Absolute numbers and percentages (%) are shown

* Possible to have more than 1 presenting symptom

¥ Percentage of children in that specific variable group

For peer review only

SUPPLEMENTARY FILE 6

Appendix G: Selection of quotes from parents

Severity of illness – quotes based on 32 open text answers

Positive experiences	Negative experiences
**Health care services**	
"Normally the waiting time at the emergency department can take hours. However, now we were seen and treated within 30 minutes. This spared my child hours of pain !!! Thus a very positive experience!!!!"	"My son had to undergo surgery in a tertiary hospital. Normal waiting time would be 2 weeks, but now it was extended to 1.5 month. During these times, he cried a lot and was in a lot of pain; pure hell."
"She underwent surgery on her heart defect during the lockdown."	"The doctor couldn't see my son, so the severity of the wound wasn't clear. The advice "to call back in a few days" wasn't of much help…"
	"Because my child was not seen at the GP out of office for his double ear infection, he received his antibiotic treatment later."
**Parents' behaviour**	
	"We waited a day / night too long to seek medical help out of fear."
**Reported health care professionals' behaviour**	
"The emergency department was empty and we were nearly the only ones at the paediatric ward. The doctors had a lot of time for us."	"They wanted the patients to leave as soon as possible."

Comparable quotes are not separately described

Treatment – quotes based on 39 open text answers

Positive experiences	Negative experiences
**Health care services**	
" All elective surgeries were postponed and the paediatric ward has never been this empty. We received the best medical care during this period. There was enough time to properly start palliative care."	"My son needed to wait much longer on his surgery than if there had not have been a lockdown."
	"My child did not undergo surgery but received a higher dose of antibiotics."
**Parents' behaviour**	
	"We waited longer to consult the GP. Normally we would have contacted the GP earlier, but we did not out of fear of getting infected with COVID-19 and the impact of the protective clothing health care workers wore on our child."

For peer review only

Reported health care professionals’ behaviour	
	“ We have spent hours in the emergency department in an isolated room without any communication. The nurses gave us little attention and we were asked to take care of the nightshift ourselves because it was difficult for the nurses to change into protective clothing. Reason for us to go home the next day.”
	“The doctor could not see us for a consultation. I felt unheard and not understood.”
	“Our GP was very worried, leading to daily consultations while it all started with a simple fever.”

Comparable quotes are not separately described

For peer review only

STROBE 2007 (v4) checklist of items to be included in reports of observational studies in epidemiology*
Checklist for cohort, case-control, and cross-sectional studies (combined)

Section/Topic	Item #	Recommendation	Reported on page #
**Title and abstract**	1	(*a*) Indicate the study's design with a commonly used term in the title or the abstract	The study design is stated in the title and abstract. Page 1-2
		(*b*) Provide in the abstract an informative and balanced summary of what was done and what was found	The abstract is written according to the *BMJ Open* guidelines with all required information. Page 1-2
**Introduction**			
Background/rationale	2	Explain the scientific background and rationale for the investigation being reported	The background and rationale are described in the first three paragraphs of the introduction. Page 4
Objectives	3	State specific objectives, including any pre-specified hypotheses	The objective is stated in the last paragraph of the introduction. Page 5
**Methods**			
Study design	4	Present key elements of study design early in the paper	Key elements of the study design are presented in the first paragraph of the methods section, and in detail explained in the consequent

For peer review only

			paragraphs. Page 5
Setting	5	Describe the setting, locations, and relevant dates, including periods of recruitment, exposure, follow-up, and data collection	The setting, location and relevant dates are described in the paragraphs 'Study population and setting' and 'Data collection' in the methods section. Page 5-6
Participants	6	(*a*) *Cohort study*—Give the eligibility criteria, and the sources and methods of selection of participants. Describe methods of follow-up<br>*Case-control study*—Give the eligibility criteria, and the sources and methods of case ascertainment and control selection. Give the rationale for the choice of cases and controls<br>*Cross-sectional study*—Give the eligibility criteria, and the sources and methods of selection of participants	Described in the paragraphs 'Study population and setting' and 'data collection' in the methods section. There was no follow-up. Page 5-6
		(*b*) *Cohort study*—For matched studies, give matching criteria and number of exposed and unexposed<br>*Case-control study*—For matched studies, give matching criteria and the number of controls per case	Not applicable.
Variables	7	Clearly define all outcomes, exposures, predictors, potential confounders, and effect modifiers. Give diagnostic criteria, if applicable	Described in the paragraphs 'Data collection' and 'Data analysis'. Page 6-8
Data sources/ measurement	8*	For each variable of interest, give sources of data and details of methods of assessment (measurement). Describe comparability of assessment methods if there is more than one group	Described in the paragraphs 'Data analysis'. Page 7-8
Bias	9	Describe any efforts to address potential sources of bias	The survey was disseminated via social media to reach the general population (minimise

For peer review only

			selection bias). Page 6
Study size	10	Explain how the study size was arrived at	A sample size calculation was performed described in the paragraph 'Study population and setting' in the methods section. Page 5
Quantitative variables	11	Explain how quantitative variables were handled in the analyses. If applicable, describe which groupings were chosen and why	Described in 'Data analysis'. Page 7-8
Statistical methods	12	(*a*) Describe all statistical methods, including those used to control for confounding	Described in 'Data analysis'. Page 7-8
		(*b*) Describe any methods used to examine subgroups and interactions	Described in 'Data analysis'. Page 7-8
		(*c*) Explain how missing data were addressed	Not applicable.
		(*d*) *Cohort study*—If applicable, explain how loss to follow-up was addressed<br>*Case-control study*—If applicable, explain how matching of cases and controls was addressed<br>*Cross-sectional study*—If applicable, describe analytical methods taking account of sampling strategy	Not applicable.
		(*e*) Describe any sensitivity analyses	Not applicable.
**Results**			
Participants	13*	(a) Report numbers of individuals at each stage of study—eg numbers potentially eligible, examined for eligibility, confirmed eligible, included in the study, completing follow-up, and analysed	Numbers included during the first round of the survey and the second round of the survey are stated in the first paragraph of the results section. All tables and figures included the number of patients that were analysed. Page 8-10

For peer review only

		(b) Give reasons for non-participation at each stage	Not applicable.
		(c) Consider use of a flow diagram	Not applicable.
Descriptive data	14*	(a) Give characteristics of study participants (eg demographic, clinical, social) and information on exposures and potential confounders	Characteristics of the study population are described in table 1 and table 2. Page 8-10
		(b) Indicate number of participants with missing data for each variable of interest	Not applicable.
		(c) *Cohort study*—Summarise follow-up time (eg, average and total amount)	Not applicable.
Outcome data	15*	*Cohort study*—Report numbers of outcome events or summary measures over time	The numbers of the outcomes are reported in the paragraphs 'Parents' response to a sick or injured child' and 'Impact of changes in health services'. Page 10-12
		Case-control study—Report numbers in each exposure category, or summary measures of exposure	Not applicable.
		Cross-sectional study—Report numbers of outcome events or summary measures	Not applicable.
Main results	16	(*a*) Give unadjusted estimates and, if applicable, confounder-adjusted estimates and their precision (eg, 95% confidence interval). Make clear which confounders were adjusted for and why they were included	Absolute numbers and percentages are given. Chi-squared tests were used for comparing groups stratified for children with and without chronic condition. Page 8-12
		(*b*) Report category boundaries when continuous variables were categorized	Described in section 'Data analysis' in methods section.

For peer review only

			Page 7
		(*c*) If relevant, consider translating estimates of relative risk into absolute risk for a meaningful time period	Not applicable.
Other analyses	17	Report other analyses done—eg analyses of subgroups and interactions, and sensitivity analyses	Additional thematic analysis for qualitative data was performed and described in 'Data analysis' and in the last paragraph of the results section. Page 8 and page 12
**Discussion**			
Key results	18	Summarise key results with reference to study objectives	Key results are stated in the first paragraph and discussed in the subsequent paragraphs of the discussion section. Page 12-13
Limitations	19	Discuss limitations of the study, taking into account sources of potential bias or imprecision. Discuss both direction and magnitude of any potential bias	Limitations of the study are addressed in the paragraph 'Strengths and limitations' of the discussion section. Several potential biases are described. Page 13-14
Interpretation	20	Give a cautious overall interpretation of results considering objectives, limitations, multiplicity of analyses, results from similar studies, and other relevant evidence	Interpretation of the results is described in the discussion

For peer review only

			section. Page 12-14
Generalisability	21	Discuss the generalisability (external validity) of the study results	Generalizability of the results is discussed in the paragraph 'Strengths and limitations'. Page 13-14
**Other information**			
Funding	22	Give the source of funding and the role of the funders for the present study and, if applicable, for the original study on which the present article is based	Funding statement at the end of the manuscript. Page 17

*Give information separately for cases and controls in case-control studies and, if applicable, for exposed and unexposed groups in cohort and cross-sectional studies.
Note: An Explanation and Elaboration article discusses each checklist item and gives methodological background and published examples of transparent reporting. The STROBE checklist is best used in conjunction with this article (freely available on the Web sites of PLoS Medicine at http://www.plosmedicine.org/, Annals of Internal Medicine at http://www.annals.org/, and Epidemiology at http://www.epidem.com/). Information on the STROBE Initiative is available at www.strobe-statement.org.

For peer review only

BMJ Open

PARENTS’ EXPERIENCES WITH A SICK OR INJURED CHILD DURING THE COVID-19 LOCKDOWN: AN ONLINE SURVEY IN THE NETHERLANDS

Journal:	*BMJ Open*
Manuscript ID	bmjopen-2021-055811.R2
Article Type:	Original research
Date Submitted by the Author:	15-Nov-2021
Complete List of Authors:	Tan, Chantal; Erasmus MC Sophia Children Hospital, General Paediatrics<br>Lutgert, Eveline; Erasmus MC Sophia Children Hospital, General Paediatrics<br>Neill, Sarah; University of Plymouth, Faculty of Health<br>Carter, Rachel; University of Plymouth, Faculty of Health<br>Jones, Ray; University of Plymouth, Faculty of Health, Education, and Society<br>Chynoweth, Jade; University of Plymouth, Faculty of Health<br>Borensztajn, Dorine; Erasmus MC Sophia Children Hospital, General Paediatrics<br>Lakhanpaul, Monica; University College London, Institute of Child Health<br>moll, Henriette; Erasmus MC Sophia Children Hospital, Department of Pediatrics
**Primary Subject Heading**:	Paediatrics
Secondary Subject Heading:	Health services research
Keywords:	COVID-19, PAEDIATRICS, Community child health < PAEDIATRICS

SCHOLARONE™
Manuscripts

I, the Submitting Author has the right to grant and does grant on behalf of all authors of the Work (as defined in the below author licence), an exclusive licence and/or a non-exclusive licence for contributions from authors who are: i) UK Crown employees; ii) where BMJ has agreed a CC-BY licence shall apply, and/or iii) in accordance with the terms applicable for US Federal Government officers or employees acting as part of their official duties; on a worldwide, perpetual, irrevocable, royalty-free basis to BMJ Publishing Group Ltd ("BMJ") its licensees and where the relevant Journal is co-owned by BMJ to the co-owners of the Journal, to publish the Work in this journal and any other BMJ products and to exploit all rights, as set out in our licence.

The Submitting Author accepts and understands that any supply made under these terms is made by BMJ to the Submitting Author unless you are acting as an employee on behalf of your employer or a postgraduate student of an affiliated institution which is paying any applicable article publishing charge ("APC") for Open Access articles. Where the Submitting Author wishes to make the Work available on an Open Access basis (and intends to pay the relevant APC), the terms of reuse of such Open Access shall be governed by a Creative Commons licence – details of these licences and which Creative Commons licence will apply to this Work are set out in our licence referred to above.

Other than as permitted in any relevant BMJ Author's Self Archiving Policies, I confirm this Work has not been accepted for publication elsewhere, is not being considered for publication elsewhere and does not duplicate material already published. I confirm all authors consent to publication of this Work and authorise the granting of this licence.

For peer review only

PARENTS' EXPERIENCES WITH A SICK OR INJURED CHILD DURING THE COVID-19 LOCKDOWN: AN ONLINE SURVEY IN THE NETHERLANDS

Chantal D. Tan[1], Eveline K. Lutgert[1], Sarah Neill[2], Rachel Carter[2], Ray Jones[2], Jade Chynoweth[2], Dorine M. Borensztajn[1], Monica Lakhanpaul[3], Henriette A. Moll[1]

Author affiliations:

1. Erasmus MC-Sophia Children's Hospital, Department of General Paediatrics, Rotterdam, the Netherlands
2. Faculty of Health, University of Plymouth, United Kingdom
3. Integrated Community Child Health Population, Policy & Practice Department, UCL Great Ormond Street Institute of Child Health, University College London, United Kingdom

Corresponding author:

Chantal D. Tan, MD

Department of General Paediatrics, Erasmus MC – Sophia Children's Hospital

P.O. Box 2060, 3000 CB, Rotterdam, the Netherlands

Telephone: +31 10 703 72 39

E-mail: c.tan@erasmusmc.nl

Word count: 3994 words

For peer review only

For peer review only - http://bmjopen.bmj.com/site/about/guidelines.xhtml

Abstract

Objective: To assess the impact of the COVID-19 lockdown on parents' health seeking behaviour and care for a sick or injured child in the Netherlands.

Design and setting: An online survey on parents' experiences with a sick or injured child during the COVID-19 lockdown periods was disseminated through social media.

Participants: Parents living in the Netherlands with a sick or injured child during the lockdown periods from March until June 2020 and from December 2020 until February 2021 were eligible to participate.

Outcome measures: Descriptive statistics and thematic analysis were used to analyse family and children's characteristics, parents' response to a sick or injured child, and the perceived impact of the lockdown on child's severity of illness and treatment reported by parents. Analyses were stratified for children with and without chronic conditions.

Results: Of the 105 parents who completed the survey, 83% reported they would have sought medical help before lockdown compared to 88% who did seek help during lockdown for the same specific medical problem. Parents reported that changes in health services affected their child's severity of illness (31%) and their treatment (39%), especially for children with chronic conditions. These changes included less availability of health care services and long waiting lists, which mostly led to worsening of the child's illness. During lockdown, there was no change in health seeking behaviour by parents of children with a chronic condition (N=51) compared to parents of children without a chronic condition.

Conclusion: Parents in the Netherlands who completed the survey were not deterred from seeking medical help for their sick or injured child during the COVID-19 lockdown periods. However, changes in health services affected child's severity of illness and treatment, especially for children with chronic conditions.

For peer review only

Article summary

Strengths and limitations of this study

• Online surveys enable the collection of anonymised data and facilitate the collection of data from a wide range of patients regardless of their residency.

• The survey was disseminated through social media to reach the general population.

• The survey had several 'other' options where parents could give a more detailed explanation of their answers in addition to the multiple-choice answers.

• The advertisement through social media could have caused selection bias as parents who do not have social media or parents with limited (digital) literacy could not have filled in the survey.

• Our study population might not be a good reflection of the general population of ill or injured children as almost 50% of the children had a chronic condition.

For peer review only

Background

Globally, health care professionals reported that the number of paediatric patients visiting medical services declined significantly while lockdowns were in effect due to the Coronavirus disease 2019 (COVID-19) pandemic: the estimated decline of paediatric visits to Emergency Departments (EDs) during lockdown ranged from 30% in the United Kingdom to 89% in Italy.(1-4) In the Netherlands all medical visits to Dutch EDs declined by 25% during the first lockdown from March 23rd until June 1st 2020.(5) The Dutch Healthcare Authority has reported that paediatric urgent referrals by general practitioners (GPs) declined more than referrals by GPs to other specialties.(6) This decline could partially be explained by a lower incidence of illness or injury in children during lockdown as limited contact with others reduced exposure to infectious diseases and restricted activities reduced accidental injuries.(7, 8) However, other specialties have shown a recuperation of the number of referrals after the first lockdown, while the number of paediatric referrals has not.(6) The Netherlands had two lockdown periods where civilians were strongly advised to mainly stay at home and schools were closed.

Other explanations for the decline in paediatric medical visits are parental concern about COVID-19 and barriers raised by healthcare professionals.(9) On the one hand, there may be positive outcomes from parents' self-care of their children as they develop ways of coping with and managing their child's illness or injury themselves. On the other hand, it is also important to notice the possible negative outcomes of the decrease in parents seeking help for a sick or injured child. In a survey set out by the Dutch Paediatric Society amongst 1400 Dutch paediatricians, there were 51 cases reporting unnecessary collateral harm as a result of delayed presentation to medical health services due to the COVID-19 pandemic. These reports include several cases of diabetic ketoacidosis, sepsis, and cancer.(10) Similar results were found in surveys set out in other European countries.(9, 11, 12) Unfortunately, these surveys do not differentiate between delay caused by parents or by health care professionals.

It is imperative that we try to unravel the reasoning behind the health seeking behaviour of parents for ill or injured children and the impact that the COVID-19 lockdown had on their behaviour. Special attention should be given to parents of children with chronic conditions. They may seek less medical care as they are afraid of COVID-19 infection for their vulnerable child. Concerns were also raised regarding the mental health of children and adolescents during these difficult times. Based on a survey in the United States, 35% of

For peer review only - http://bmjopen.bmj.com/site/about/guidelines.xhtml

adolescents receive mental health support solely in an educational setting.(13) With schools closing, it is possible that certain services could not be accessed properly.(14) Children also ended up spending more time at home and were socially isolated, which may have negatively affected their mental health.(15, 16)

We aimed to assess the impact of the COVID-19 lockdown on parents' experiences with a sick or injured child in the Netherlands, stratified for children with and without a chronic condition. In more detail, we focus on whether and where parents sought help and whether and why their health seeking behaviour had changed due to the lockdown period. In addition, we assessed parents' perceptions of the impact of the lockdown on their child's severity of illness and treatment.

For peer review only

Methods

Study design

This study was a COVID-19 related project in collaboration with the University College London and the University of Plymouth. It was a cross-sectional study consisting of an online survey assessing parents' health seeking behaviour for, and care of, a sick or injured child during two COVID-19 lockdown periods. Approval by the ethical committee of the Erasmus MC Sophia was obtained. Informed consent of the participating parents was obtained.

Study population and setting

Parents living in the Netherlands who self-identified as having a sick or injured child during the lockdown periods from March 23rd until June 1st 2020 and from December 15th 2020 until February 16th 2021 were included. The main measures taken during these lockdown periods are shown in chronological order in Appendix A. *(Supplementary file 1)* We excluded parents who did not live in the Netherlands or who did not have a sick or injured child during the afore mentioned lockdown periods. Our aim was to recruit about 100 respondents to our survey. For a sample size of 100, the 95% confidence interval for a 50% estimate of proportions is 40.2% to 59.8% to compare the proportion of parents who sought help before lockdown to the proportion of parents that sought help during the lockdown. For a sample size of 50 in each subgroup of children with and without comorbidity the 95% confidence interval for a 50% estimate proportion is 36.2% to 63.8%.(17, 18)

For peer review only - http://bmjopen.bmj.com/site/about/guidelines.xhtml

Data collection

The original English questionnaire was translated to the Dutch language. The questions were similar, but the answer options regarding the health care system were adapted to the Dutch health care system. *(Supplementary file 2 Appendix B, Supplementary file 3 Appendix C)*. The questionnaire was launched as an online survey created in Google Forms, which enabled the collection of anonymised data. Online surveys facilitate the collection of data from a wide range of parents regardless of their residency. The survey was mainly composed of multiple-choice questions, but 'other' options to add free text were available. This 'other' option also enabled parents to give a more detailed explanation on their previous chosen multiple-choice answer. Respondents were recruited using social media and virtual snowball sampling, in which people helped to disseminate the survey to other parents by reposting the survey on their own social media channels. Information about the survey was posted by the research group on Facebook, Twitter, LinkedIn, Instagram, WhatsApp and professional contacts were sent a request to share the information about the study with their social contacts. Facebook was divided into personal Facebook accounts and Facebook groups, of which the latter included private Facebook groups for parents as well as public Facebook pages of the Erasmus MC and the Sophia Children's Hospital. A short introduction to the survey and a link to the survey was written to share on social media *(Supplementary file 4 Appendix D).* A reminder was posted on social media every two weeks until the end of the survey period. When the number of new respondents stagnated we decided to end the survey period. Key persons who had shared the survey on their social media and their estimated reach were recorded in a document to keep track of the spread of the survey and the number of respondents. There was no incentive provided to parents for participating.

Patient and public involvement

The public was involved in the design and conduct of the study. Parents have been involved in the project as research team members and reviewed the original survey developed in the United Kingdom. In the Netherlands, feedback on the survey and the total duration to fill in the survey came from two parents who were research team members and three parents not working in the medical field. Additionally, feedback on the survey was given by paediatricians and PhD students of the research group of General Paediatrics Erasmus MC Sophia. However, modifications were not needed and these research group members and parents helped disseminating the survey on social media. We plan to disseminate the results to

For peer review only

For peer review only - http://bmjopen.bmj.com/site/about/guidelines.xhtml

clinicians and health policy makers so that decisions concerning access to health services and support for parents can be made based on evidence.

Ethical considerations

The study was approved by the Medical Ethics Committee Erasmus Medical Centre (MEC-2020-0627) and by the Scientific Research Committee SARS-CoV-2 and COVID-19 Erasmus Medical Centre. Information about the study and its purpose was explained at the beginning of the online survey. The survey introduction also included a statement about the anonymity of the responses, which explained that no personal identifiable data would be collected. At the end of this information section parents were asked to check a statement, which clearly stated that choosing to complete and submit responses to the survey gave consent to their responses being used in our study. Since we were only including parents in the survey and not the children themselves, there were no concerns about capacity to consent. Information on data usage and dissemination was also provided. Lastly, the respondents had the opportunity to contact the research team if they had additional questions or if they wanted to receive a summary of the study findings. Email addresses were provided for both the project lead and an independent paediatrician to whom any concerns or complaints could be directed.

Data analysis

Data were analysed using SPSS software version 25.0 and free text data was subjected to thematic analysis. Firstly, descriptive statistics were used for the characteristics of parents and families who completed the survey. Information on living area, access to WiFi, children in the family, and whether they have laptops, computers or mobile phones, was collected.

Secondly, descriptive statistics for children's characteristics, stratified for chronic condition, were used. The following question on chronic conditions was included in the survey: "Does your child have any pre-existing illnesses, such as chronic or long term illness, complex needs or a recurring illness?" When parents answered 'yes' they were asked to specify what kind of chronic condition or recurring illness their child had. Children's characteristics, including age, gender, presenting symptoms and hospital admission, were collected as well. Age was categorised into three predefined age groups: <5 years, 5-11 years and 12-17 years. Presenting symptoms were categorised into the following eight subgroups: skin, breathing, body temperature, dehydration, pain, change of behaviour, injury and other.

Thirdly, parents' response to a sick or injured child was described, stratified for chronic condition. The parents were asked what medical symptom(s) their child had during the lockdown and the subsequent questions were 1) whether they would have sought help for the

For peer review only - http://bmjopen.bmj.com/site/about/guidelines.xhtml

same problem before lockdown and 2) whether they did seek help for this problem during lockdown. Lastly, we analysed whether changes in health services during the lockdown affected severity of illness of the child and the child's treatment, stratified for chronic condition. McNemar's test and X^2-tests were used for quantitative analyses with a p-value <0.05 considered as statistically significant. Qualitative analyses using thematic analysis according to Braun and Clarke were used for free text answers from parents on questions regarding the impact of changes in health services on severity of illness of the child and the treatment the child received.(19) Two authors (CT and EL) categorised the free text answers independently to minimise subjectivity of a single researcher's judgement. A selection of quotes from parents regarding the perceived impact of changes in health services were translated (by CT and EL) and divided into three categories: health care services, parents' behaviour and reported health care professionals' behaviour. The quotes were categorized into negative and positive experiences perceived by parents.

Results

A total of 105 respondents filled in the online COVID-19 parent survey in the Netherlands. During the two-month period from October 19th to December 19th 2020, 76 respondents completed the survey regarding the first lockdown period. During the second lockdown, an additional 29 respondents completed the survey from January 16th to February 16th 2021. Figure 1 shows through which social media channel the online survey had reached parents. The online survey reached 60% of the parents via Facebook (personal and groups), 28% via WhatsApp, and 12% via Twitter and LinkedIn.

Parent and family characteristics

The parent and family characteristics are shown in table 1. Most families came from small cities in an urban area (69%) and nearly half were from the province South-Holland (47%). All parents had access to WiFi, nearly all had laptops or computers (95%), mobile phones (90%) and access to outdoor space during lockdown (97%). The majority of the school aged children in the family were staying at home as schools were closed (76%) when the illness or injury happened during lockdown.

For peer review only

Table 1: Parent and family characteristics (N=105)

	Parent of sick or injured child
**Description of living area**	
Rural – village	6 (6)
Urban - small city	72 (69)
Urban – big city	27 (26)
**Province**	
South Holland	49 (47)
Zeeland	15 (14)
North Brabant	12 (11)
The other 7 provinces	29 (28)
**WiFi access**	105 (100)
**Mobile phone**	95 (90)
**Laptop or computer**	100 (95)
**Access to outdoor space during lockdown** (garden/balcony)	102 (97)
**Where were the school aged children in the family when the illness/injury happened during the lockdown**	
All of the children were staying at home	80 (76)
All the children were attending school	14 (13)
Some of the children were attending school	11 (11)

Absolute numbers and percentages (%) are shown

Children's characteristics

In table 2 the children's characteristics are shown for the total population and stratified for chronic condition. Forty nine percent of the children had a chronic condition or recurring illness (51/105) such as psychomotor retardation/epilepsy, scoliosis, asthma, eczema or recurring otitis media. The majority of the children were below the age of twelve and were boys. The most common presenting symptoms were pain (45%) and change of behaviour (40%). There was a higher percentage of children aged 12-17 years with chronic conditions compared to children without chronic conditions (28% versus 13%). A higher percentage of children with chronic conditions reportedly had breathing problems (29% versus 19%) and a

For peer review only - http://bmjopen.bmj.com/site/about/guidelines.xhtml

For peer review only

lower percentage had injuries (16% versus 28%) compared to children without chronic conditions. Other presenting symptoms were for example hearing problems, congenital malformations (cleft lip, scoliosis), and facial nerve paralysis. A lower percentage of children with chronic conditions were admitted to the hospital during lockdown compared to children without chronic conditions (31% versus 39%).

Table 2: Children's characteristics stratified for chronic condition

	**Total (N=105)**	**Children with chronic condition (N=51)**	**Children without chronic condition (N=54)**
**Age** (years)			
<5	41 (39)	12 (24)	29 (54)
5-11	43 (41)	25 (49)	18 (33)
12-17	21 (20)	14 (28)	7 (13)
**Gender**			
Boys	61 (58)	28 (55)	33 (61)
Girls	44 (42)	23 (45)	21 (39)
**Presenting symptom***			
Skin and appearance	22 (21)	11 (22)	11 (20)
Breathing	25 (24)	15 (29)	10 (19)
Body temperature	16 (15)	9 (18)	7 (13)
Dehydration	21 (20)	11 (22)	10 (19)
Pain	45 (45)	24 (47)	23 (43)
Change of behaviour	42 (40)	21 (41)	21 (39)
Injury	23 (22)	8 (16)	15 (28)
Other	19 (18)	7 (14)	13 (24)
**Hospital admission**			
Admitted	37 (35)	16 (31)	21 (39)

Absolute numbers and percentages (%) are shown

* Possible to have more than 1 presenting symptom

Parents' response to a sick or injured child

Parents reported that 83% (87/105) would have sought help before lockdown, whereas 88% (92/105) of the parents did seek help during lockdown. Health seeking behaviour of 76

For peer review only - http://bmjopen.bmj.com/site/about/guidelines.xhtml

parents during the first lockdown and 29 parents during the second lockdown with comparable restrictions did not differ (data not shown). Four parents reported that they would have sought help before lockdown but did not seek help during lockdown, whereas nine parents would not have sought help before lockdown but did seek help during lockdown. (McNemar's test, p=0.2) *(Supplementary file 5 Appendix E).* The thirteen parents (13/105, 12%) who decided not to ask for medical help during lockdown stated that they were not sure if their child was ill or injured enough to need medical help, were worried about themselves or a family member catching COVID-19, thought that the advice "to stay at home" meant that they could not go to a health centre or hospital or were worried about using health services when they were needed more urgently by other people. Before the lockdown, parents of children with a chronic condition would have sought help for their sick or injured child in 77% (39/51) compared to 89% (48/54) of the parents of children without a chronic condition (X^2-test, p=0.1). During the lockdown, parents of children with a chronic condition did seek help for their sick or injured child in 82% (42/51) compared to 93% (50/54) of the parents of children without a chronic condition (X^2-test, p=0.1). Health seeking behaviour before and during lockdown related to children's characteristics is shown in Appendix F. *(Supplementary file 6)* Parents of children below the age of five sought help more frequently and the percentage of parents seeking help decreased with increasing child's age. The percentage of parents seeking help for their sick or injured child was comparable for all presenting symptom groups except for the 'other' presenting symptom group.

The reported medical sources used by parents before lockdown were comparable with the reported sources used during lockdown. The majority of the parents contacted the GP and some parents sought help from their child's paediatrician or another medical professional such as the midwife. A small percentage of the parents called the Dutch emergency number or went to the ED. Parents were asked which other actions they undertook for their sick or injured child. Some parents stated that they chose to wait-and-see whether their child got better. When they did decide to treat the illness or injury themselves, they most often treated their child with paracetamol. Parents sought information on how to treat their child on the internet and by asking their social circle of friends and family. When parents stated that information and advice given were useful, this was mainly because they were reassured that what they were doing was right. A quote from a parent was "The information was useful since it reassured me, useful tips and information on when to seek medical help (again) were given". When parents stated that information and advice given were not useful, this was

For peer review only - http://bmjopen.bmj.com/site/about/guidelines.xhtml

mainly because it was general advice and not child specific. A quote of a parent was “Every child is unique and requires an individual approach”.

Impact of changes in health services

Parents reported that changes in health services during lockdown had impact on the severity of illness of their child in 31% (32/105) and on their child’s treatment in 39% (41/105). Parents’ explanations were mostly negative and included the following responses: long waiting list for diagnostics or treatment, postponed surgery or out-patient clinic consultation, no assessment by a physician, more consultations by telephone, fewer checks and doctor visits during admission. Lastly some parents stated they waited too long to seek help because they were anxious. Contrarily, there were also some positive responses including shorter turnaround time at the ED and more attention from medical staff during admission as the ward was almost empty. *(Supplementary file 7, Appendix G)* Parents of children with a chronic condition reported that changes in health services affected the severity of their child’s illness in 33% (17/51) compared to 28% (15/54) of the parents of children without a chronic condition (X^2-test, p=0.5). Parents of children with a chronic condition reported that changes in health services affected their child’s treatment in 47% (24/51) compared to 32% (17/54) of the parents of children without a chronic condition (X^2-test, p=0.1).

Discussion and conclusion

Main findings

Parents in the Netherlands with a sick or injured child during the COVID-19 lockdown and who completed the online survey, were not deterred from seeking medical help for their sick or injured child. Almost 50% of the sick or injured children in our study had a chronic condition or recurring illness. Parents of children without a chronic condition would have sought help more often before lockdown and did seek help more frequently during lockdown than parents of children with a chronic condition. This might be explained by the fact that parents of children with chronic conditions are better able to judge their child’s illness or to treat the illness themselves since they have more medical experience. However, more parents of children with a chronic condition reported that changes in health services during lockdown had impact on their child’s severity of illness and treatment compared to parents of children without a chronic condition. Parents of children with a chronic condition may have been more anxious during the COVID-19 pandemic about catching COVID-19 themselves or their child,

For peer review only - http://bmjopen.bmj.com/site/about/guidelines.xhtml

who is perceived to be more vulnerable than a healthy child. The child's age was associated with parents' health seeking behaviour as parents of children below the age of five sought medical help more frequently, both before and during lockdown. This finding is also described by Sands et al. where the majority (70%) of medical attendees in children were below the age of five.(20) Remarkably, the number of parents who did seek medical help during the lockdown was high in all presenting symptom groups. This might indicate that parents who completed the survey probably had a more severely ill child. This could also explain the high hospital admission rate (35%) in our study. It has been reported that there is an increase in mental health problems in children due to the lockdown.(21) Where breathing problems are a common presenting symptom to seek medical help, 40% of the children in our study had a change of behaviour as presenting symptom which is typical for the lockdown period.(20)

Strengths and limitations

This online survey was the first national survey in the Netherlands on parents' health seeking behaviour and care for a sick or injured child during the lockdown period due to the COVID-19 pandemic. The survey was circulated through social media to reach the general population, which was also geographically spread throughout the Netherlands. We could not estimate the reach of the survey since it was reposted by many people on social media through virtual snowball sampling. The social media channels of our hospital reached more than 10,000 people, but we could not calculate the response rate as we do not know how many of these children were ill or injured in the lockdown periods. The survey had several 'other' options where parents could give a more detailed explanation of their answers in addition to the multiple-choice answers. There were also some limitations. Firstly, the survey was advertised through social media, which could have caused selection bias since parents who do not have access to social media or who are not active on social media could not have filled in the survey. Parents with limited (digital) literacy would also have been unable to fill in the survey. Additionally, this study design was prone to sampling bias. We did not have data on parents with an injured of sick child who did not fill in the survey. It seemed that parents of children with more serious illness had filled in the survey as this was reflected in the high hospital admission rate of 35%. Therefore generalizability might be limited. The majority of the respondents live in the province South-Holland, which could be explained by the fact that Erasmus MC Sophia is located in South-Holland. This study was prone to information bias and recall bias as well. Parents were asked theoretically whether they would have sought help

For peer review only - http://bmjopen.bmj.com/site/about/guidelines.xhtml

for the same medical problem before lockdown, which was a potential information bias. Recall bias could have occurred when parents where asked about the sickness/injury of their child during the lockdown periods as these events happened before they filled in the questionnaire. However, we assumed that parents who filled in the survey do remember their child's sickness/injury well since these were probably parents of children with more serious illness reflected in the high hospital admission rate. Secondly, our study population might not be a good reflection of the general population of ill or injured children as almost 50% of the children had a chronic condition or recurring illness. This might be explained by the dissemination strategy or parents of children with a chronic condition being more motivated to participate. However, this allowed us to perform analyses stratified for chronic condition which is independent of our sample of parents who had completed the survey. Furthermore, children with a chronic condition are a vulnerable group, who are more prone to serious illness and this study provides important insights in the health seeking behaviour of their parents.

Implications for clinical practice

This study showed that the majority of parents who completed our survey did seek help for a sick or injured child during the lockdown period. However, there were barriers in the health seeking process such as postponed doctors' visits, no physician's assessment and fewer face-to-face consultations were undertaken as more were performed by telephone. A small group of parents decided not to seek help for their sick or injured child because they were not sure if their child was ill or injured enough to need medical help reflecting pre-pandemic research findings (22), were worried about catching COVID-19 or thought that the advice to stay at home meant that they could not attend medical health services. This finding is important since parents should have adequate information on when and where to seek help for a sick or injured child. However, throughout 2020 and 2021, healthcare professionals have been contacting the Dutch media to emphasize the message that EDs and hospitals continue to be available to provide care and treatment for all patients if they need medical help.(23-25)

Conclusion

Parents in the Netherlands who completed the online survey about health seeking behaviour and care for a sick or injured child during the COVID-19 lockdown were not deterred from seeking medical help for their sick or injured child. However, changes in health services did affect their child's severity of illness and treatment, especially for children with a chronic condition. Although the impact was mostly negative there were some positive consequences

For peer review only - http://bmjopen.bmj.com/site/about/guidelines.xhtml

as well. These findings are important to ensure that we inform parents, especially of children with chronic conditions, on when and where to seek help during lockdown periods to prevent delayed presentation of children with illness or injury to health services.

For peer review only

For peer review only - http://bmjopen.bmj.com/site/about/guidelines.xhtml

References

1. Isba R, Edge R, Jenner R, Broughton E, Francis N, Butler J. Where have all the children gone? Decreases in paediatric emergency department attendances at the start of the COVID-19 pandemic of 2020. Arch Dis Child. 2020;105(7):704.
2. Raucci U, Musolino AM, Di Lallo D, Piga S, Barbieri MA, Pisani M, et al. Impact of the COVID-19 pandemic on the Emergency Department of a tertiary children's hospital. Ital J Pediatr. 2021;47(1):21.
3. Morello F, Bima P, Ferreri E, Chiarlo M, Balzaretti P, Tirabassi G, et al. After the first wave and beyond lockdown: long-lasting changes in emergency department visit number, characteristics, diagnoses, and hospital admissions. Intern Emerg Med. 2021:1-8.
4. Sokoloff WC, Krief WI, Giusto KA, Mohaimin T, Murphy-Hockett C, Rocker J, et al. Pediatric emergency department utilization during the COVID-19 pandemic in New York City. Am J Emerg Med. 2021;45:100-4.
5. Toet H, Sprik, E. & Blatter, B. Effecten van de Corona maatregelen op SEH-bezoeken? Stand van zaken tot en met september 2020. Kenniscentrum letselpreventie; 2020.
6. Zorgautoriteit dN. Analyse van de gevolgen van de coronacrisis voor de reguliere zorg 2020 [updated 29-04-2020. Available from: https://puc.overheid.nl/nza/doc/PUC_306627_22/1/.
7. Hassoun A, Pugh S, Merced I, Sharma M. The power of social isolation on paediatric emergency visits during COVID-19 lockdown. Acta Paediatr. 2020;109(11):2425-6.
8. Keays G, Friedman D, Gagnon I. Injuries in the time of COVID-19 Les blessures au temps de la COVID-19. Health Promot Chronic Dis Prev Can. 2020;40(11-12):336-41.
9. Lazzerini M, Barbi E, Apicella A, Marchetti F, Cardinale F, Trobia G. Delayed access or provision of care in Italy resulting from fear of COVID-19. Lancet Child Adolesc Health. 2020;4(5):e10-e1.
10. Jansen D, Illy KE. Delayed presentation to regular Dutch paediatric care in COVID-19 times: a national survey. BMJ Paediatr Open. 2020;4(1):e000834.
11. Lynn RM, Avis JL, Lenton S, Amin-Chowdhury Z, Ladhani SN. Delayed access to care and late presentations in children during the COVID-19 pandemic: a snapshot survey of 4075 paediatricians in the UK and Ireland. Arch Dis Child. 2021;106(2):e8.
12. Ciacchini B, Tonioli F, Marciano C, Faticato MG, Borali E, Pini Prato A, et al. Reluctance to seek pediatric care during the COVID-19 pandemic and the risks of delayed diagnosis. Ital J Pediatr. 2020;46(1):87.
13. Ali MM, West K, Teich JL, Lynch S, Mutter R, Dubenitz J. Utilization of Mental Health Services in Educational Setting by Adolescents in the United States. J Sch Health. 2019;89(5):393-401.
14. Golberstein E, Wen H, Miller BF. Coronavirus Disease 2019 (COVID-19) and Mental Health for Children and Adolescents. JAMA Pediatr. 2020;174(9):819-20.
15. Rauschenberg C, Schick A, Goetzl C, Roehr S, Riedel-Heller SG, Koppe G, et al. Social isolation, mental health, and use of digital interventions in youth during the COVID-19 pandemic: a nationally representative survey. Eur Psychiatry. 2021:1-32.
16. Chen X, Qi H, Liu R, Feng Y, Li W, Xiang M, et al. Depression, anxiety and associated factors among Chinese adolescents during the COVID-19 outbreak: a comparison of two cross-sectional studies. Transl Psychiatry. 2021;11(1):148.
17. Raosoft. Sample size calculator: Raosoft Inc.; [Available from: http://www.raosoft.com/samplesize.html.
18. Systems CR. Sample Size Calculator: Creative Research Systems; [Available from: https://www.surveysystem.com/sscalc.htm.
19. Braun V, Clarke V. Using thematic analysis in psychology. Qual Res Psychol 2006:3: 77 - 101.

For peer review only - http://bmjopen.bmj.com/site/about/guidelines.xhtml

20. Sands R, Shanmugavadivel D, Stephenson T, Wood D. Medical problems presenting to paediatric emergency departments: 10 years on. Emerg Med J. 2012;29(5):379-82.
21. Newlove-Delgado T, McManus S, Sadler K, Thandi S, Vizard T, Cartwright C, et al. Child mental health in England before and during the COVID-19 lockdown. Lancet Psychiatry. 2021.

22. Neill SJ, Jones CH, Lakhanpaul M, Roland DT, Thompson MJ. Parents' help-seeking behaviours during acute childhood illness at home: A contribution to explanatory theory. J Child Health Care. 2016;20(1):77-86.
23. Willems I. Ziekenhuis St Jansdal slaat alarm over zorgmijders: ‘Er staat hier écht geen meneer corona voor de deur’: De Stentor; 2021 [updated 19-02-2021. Available from: https://www.destentor.nl/veluwe/ziekenhuis-st-jansdal-slaat-alarm-over-zorgmijders-er-staat-hier-echt-geen-meneer-corona-voor-de-deur-br~a76a3e50/?referrer=https%3A%2F%2Fwww.google.com%2F.
24. Sevil M. Huisartsen: kom met serieuze klachten wel langs: Het Parool; 2020 [updated 30-03-2020. Available from: https://www.parool.nl/nieuws/huisartsen-kom-met-serieuze-klachten-wel-langs~b8e4495b/.
25. NOS. Zorgen op spoedeisende hulp om wegblijvende patiënten: 'Kom vooral wel': NOS; 2020 [updated 27-03-2020. Available from: https://nos.nl/artikel/2328538-zorgen-op-spoedeisende-hulp-om-wegblijvende-patienten-kom-vooral-wel.html.

For peer review only

For peer review only - http://bmjopen.bmj.com/site/about/guidelines.xhtml

Text for figure 1:

Figure 1: Pie chart demonstrating through which social media channel the online survey had reached parents

Authors contributions

Chantal Tan and Eveline Lutgert contributed to the conceptualisation and design of the study, coordinated the data collection, carried out the analyses, and drafted the initial and final manuscript. Dorine Borensztajn, Sarah Neill, Rachel Carter, Ray Jones, Jade Chynoweth and Monica Lakhanpaul contributed to the conceptualisation and design of the study, supervised the analyses and critically reviewed the manuscript. Henriette Moll contributed to conceptualisation and design of the study, contributed to interpretation of the data, critically reviewed the manuscript and supervised the study. All authors approved the final manuscript as submitted and agree to be accountable for all aspects of the work.

Funding statement:

This project has received funding from the European Union's Horizon 2020 research and innovation programme under grant agreement No. 848196. The funding sources had no role in the design and conduct of the study; collection, management, analysis, and interpretation of the data; preparation, review, or approval of the manuscript; and decision to submit the manuscript for publication.

Competing interests statement:

There are no competing interests.

Patient consent for publication:

Not required.

Ethics approval:

The study was approved by the Medical Ethics Committee Erasmus Medical Centre (MEC-2020-0627) and by the Scientific Research Committee SARS-CoV-2 and COVID-19 Erasmus Medical Centre.

For peer review only - http://bmjopen.bmj.com/site/about/guidelines.xhtml

Data availability statement:

Data are available upon request. Data from this study are available upon request to the corresponding author of the study (c.tan@erasmusmc.nl), subject to local rules and regulations.

For peer review only

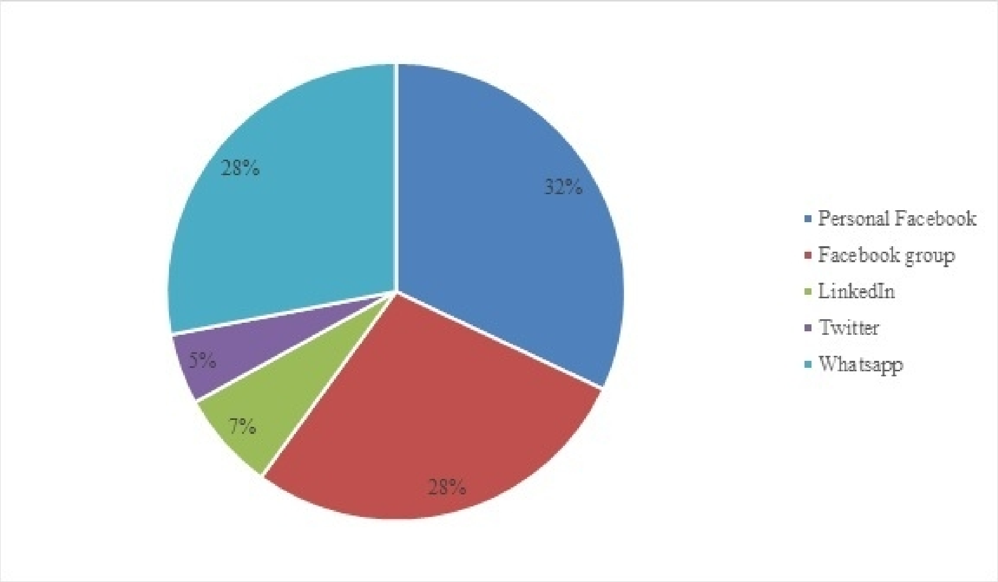

Figure 1: Pie chart demonstrating through which social media channel the online survey had reached parents

48x28mm (300 x 300 DPI)

SUPPLEMENTARY FILE 1

Appendix A: Timeline of measures taken during the first and second lockdown period

**Date**	**Measures**
	First lockdown March 23rd – June 1st 2020
March 23rd	- > 2 persons in public area prohibited with the exception of one household<br>- Group formation only with 1.5 meter distancing rule, maximum 100 persons<br>- Closing of catering industries and schools<br>- Contact professions prohibited<br>- Stores open with 1.5 meter distancing rule and hygiene measures<br>- Households can have maximum of 3 persons visiting them with 1.5 meter distancing rule<br>- Work from home as much as possible<br>- In case of violation 390 euro fine, companies 4350 euro fine
May 11th	- Children day-care and primary schools reopening (in smaller groups)<br>- Contact professions reopening<br>- Playing sports outside allowed with 1.5 meter distance<br>- Opening libraries
June 1st	- Face mask mandatory in public transport, only use public transport when necessary<br>- Restaurants / catering industries reopening for max 30 persons, booking in advance<br>- Museums, cinemas reopening for max 100 persons<br>- High school reopening
June 8th	- Primary school fully reopened
August 6th	- Announcement of the extension of a flight restriction to European countries, the United Kingdom, all countries on the Western hemisphere.
October 14th	- Closing of catering industries

For peer review only

	- A ban on selling alcohol between 8 pm and 7 am<br>- Maximum of 3 visitors per home, maximum of 30 people in interior spaces, outside no more than 4 people from other households.<br>- A ban on late night shopping
December 1st	- Facemasks become mandatory in public places, in schools, in public transport and for contact professions
	Second lockdown December 15th 2020 - February 16th 2021
December 15th	- Closing of non-essential shops<br>- Closing of theatres, cinemas, museums, zoo's, sauna's and libraries<br>- Contact professions closing (not including physiotherapists)<br>- Closing of restaurants and schools (schools starting from December 16th)<br>- Households can have maximal 2 persons visiting them with 1.5 meter distancing rule (on Christmas a maximum of 3 persons)<br>- Work from home as much as possible
December 29th	- Travellers to the Netherlands need to provide a negative PCR-test (not older than 72 hours)
January 6th	- Start of the vaccination program (healthcare workers from the emergency medicine)
January 23rd	- Mandatory curfew from 09 pm until 04.30 am
February 8th	Primary school and day-care reopening

For peer review only

SUPPLEMENTARY FILE 2

Appendix B: Original English version of the online survey

Thank you for helping us with our survey. We would like to find out what impact the Stay Home period is having on parent's actions and decision when caring for, or seeking help for, a sick or injured child. The Stay Home period is sometimes called the lockdown that started on the 23rd March when the government put restrictions on movements to stop the spread of COVID-19. The findings will help us to learn from parents' experiences and direct the development of support for parents with a sick or injured child in the future.

If your child has been ill or injured while you have been following government advice to Stay Home, we would be most grateful if you could complete our survey. We would like to know about your experiences of caring for a sick or injured child at this time, whether or not you decided to seek medical help. We would like parents to complete the survey whether or not their child needed hospital care.

If you do kindly complete and submit the survey this will indicate that you are happy for us to use your responses in our project. Your responses are, of course, anonymous so it will not be possible to identify your replies to remove them after you have completed the survey. Your survey responses will go directly to the survey team.

We will write up the findings from the project and share these in academic journals, in communication with health service leaders and policy makers, at conferences, in education, in future research projects and online. If you provide any free text comments your words may be used but it will not be possible to identify you. If you would like a summary of our findings please contact one of the project leads.

The leads for this project are Professor Sarah Neill sarah.neill@plymouth.ac.uk and Rachel Carter r.carter@plymouth.ac.uk

I can confirm that I have read the information above, I am happy to complete the questionnaire and I am happy for the information I give to be used for the project. Tick box

Are you a UK resident with a child who has been ill or injured since you were asked to Stay Home?

Yes – directed to the questionnaire

No - we thank you for your interest but we are only looking for parents living in the UK whose children have been ill or injured whilst being asked to Stay Home. We hope that you and your family stay well during this period.

For peer review only

It may be that your child has had a number of illnesses or injuries in the last few weeks since you were asked to stay at home. We would like you to select the most serious of these and answer the remaining questions with that in mind.

Question 1 About the child who was ill or injured

a. How old is your child?
   - ○ Under 12 months old
   - ○ 12 months and under 24 months old
   - ○ 2 years and under 5 years old
   - ○ 5 years and under 12 years old
   - ○ 12 years and under 16 years old
   - ○ 16 and under 18 years old

b. Was your child born a girl or boy?
   Girl
   Boy

c. Does your child have any pre-existing illnesses, such as chronic or long term illness, complex needs or a recurring illness?

If yes, please tell us what they are:

Question 2 What signs or symptoms of illness or injury did your child have? Please tick all that apply

Skin and appearance

- Skin very pale or blue, or the inside of their lips and tongue are blue
- Skin appeared mottled
- Rash which disappeared when pressed
- Rash which did not disappear with pressure (the 'Glass test')

Breathing difficulties

- Breathing stopped for a long time (more than 10 seconds at a time),
- Breathing paused (apnoeic episodes) for 5-10 seconds
- Struggling to breathe
- Sucking in muscles under their ribs, between the ribs or at the neck with each breath
- Grunting with the effort of trying to breathe
- Breathing faster than normal
- Noisy breathing (wheeze or a stridor)

Body temperature

- Under 3 months of age with a temperature of over 38°C/100.4°F
- 3 and 6 months of age with a temperature over 39°C/102.2°F

For peer review only

- Over 6 months old with a temperature above 38ºC/100.4ºF for more than 5 days
- Under 1 month of age and had a low temperature below 36ºC/96.8ºF

Dehydration (seemed to be too dry)

- Dry mouth
- Sunken eyes
- No tears
- Drowsy
- Passing less urine than usual
- Passing more urine than usual
- Drinking more than normal

Pain

- Tummy pain
- Back pain
- Painful testicles
- Headache
- Persistent pain in an injured arm or leg
- Earache
- Sore throat

Change in behaviour

- Agitated
- Persistent crying/inconsolable
- Fit/seizure
- Confused
- Wouldn't wake up or, if woken up, they wouldn't stay awake
- Unresponsive

Injury

- Burn
- Head injury
- Leg or arm injury
- Fall
- Cut(s)
- Bleeding
- Limping
- Difficulty in moving a part of the body

Other, please give details

For peer review only

Question 3 Before the advice to Stay Home, what would your *usual* response have been to this illness or injury? (Please tick one only)

- Care for my child at home
- Ask for medical help
- Something else, please give details

If you would *normally* have asked for medical help please go to Question 4

If you would *not normally* have asked for medical help please go to Question 5

If you answered *something else*, please go to Question 5

Question 4 Where would you *normally* have asked for medical help for your child with this illness or injury <u>in the first instance</u>? (Choose one only)

- NHS Direct 111
- GP surgery
- Via my GP's website
- Video consultation
- Walk in centre
- Urgent care centre
- Minor injuries unit
- GP Out of Hours service
- Calling 999
- Accident and Emergency
- Somewhere else, please say where……

Question 5 Did you ask for medical help for your child during this most recent illness or injury?

Yes, please continue to Question 6

No, please go to Question 9

Question 6 Where did you ask for medical help during this most recent illness or injury? (Please tick all that apply)

- NHS Direct 111
- GP surgery
- Via my GP's website

For peer review only

- Video consultation
- Walk in centre
- Urgent care centre
- Minor injuries unit
- GP Out of Hours service
- Called 999
- Accident and Emergency
- Somewhere else, please say where……

Question 7 Was your child admitted to hospital for this most recent illness or injury? Yes/No

If yes go to question 10

If no, continue to the next question

Question 8 What, if anything else, would you like to tell us about your experience, for example, whether advice was helpful or not helpful.

Go to Question 10 for all respondents

Question 9 Why did you decide not to ask for medical help? Please tick all that apply

- I was not sure if my child was ill or injured enough to need medical help
- I was worried about myself or a member of my family catching COVID19
- I was worried that I might be criticised for using the service if it wasn't an emergency
- I thought the Stay Home advice meant I couldn't go to a health centre or a hospital
- I was worried about using a service when it is needed more urgently by other people
- I was worried that the service would be extremely busy and that I would have to wait for too long
- I don't have a car and didn't want to use public transport
- I don't have anyone to look after my other children
- Something else - please give details

Question 10 What else did you do about your child's illness or injury? Please tick all that apply

a. I waited to see whether or not my child got better
b. I treated my child's illness/injury myself with:
   - paracetamol (Calpol)
   - ibuprofen (Brufen/Junifen)

For peer review only

- a prescribed inhaler
- Vicks or Karvol
- A cold compress/ice pack
- Cleansing
- Bandaging
- A sling to rest an injured arm
- Elevated an injured limb
- Cold drinks
- Warm drinks
- Cool bath
- Warm bath
- Treats and attention
- Distracting my child
- Home remedies, please explain what these were
- Other, please tell us what this was

c. I looked for information on how to manage the illness or injury at home from:
- family
- friends
- a family health professional
- books
- TV
- magazines
- newspapers
- radio
- the internet
- an NHS app
- Other app
- Social media (such as Twitter, Facebook, Instagram, YouTube)
- Google
- NHS Choices
- GP website
- Other website
- Other: please state

d. I used information I already had from (tick all that apply):
- family
- friends
- a family health professional
- books

For peer review only

- TV
- Magazines
- Newspapers
- radio
- an NHS app
- Other app
- Social media (such as Twitter, Facebook, Instagram, YouTube)
- Google
- NHS Choices
- GP website
- Other website
- School or college course
- A mixture of these resources
- Other: please state

Question 11 Helpfulness of information

a. What, if any, information was helpful and if so why?

b. What, if any, information was unhelpful and if so why?

Question 12 Advice provided during the Stay Home period

a. Which of the following sources of advice have you seen about when to use health services while families are being told to Stay Home? Please tick all that apply

- Government sources (such as Government TV or radio broadcast, the letter from Boris Johnson, daily briefings from Government)
- NHS sources (such as NHS Choices website, GP website, 111 service announcements)
- Other expert online sources (such as University, research organisations etc)
- Experts on Social Media sites (such as Twitter, Facebook)
  - Family/Friends on Social Media sites
  - Other, please give details

b. Have you seen advice about when to (Please tick all that apply)
  - call NHS111?
  - call your GP/family doctor?
  - attend accident and emergency with your children?

c. What, if anything, was new about advice on health service use since you have been told to Stay Home?

For peer review only

Question 13 Have the changes to health services during the Stay Home period affected how ill your child has been? Yes, Maybe, No

d. If yes, please tell us how.

Question 14 Have the changes to health services during the Stay Home period affected any treatment your child received? Yes, Maybe, No, Not applicable

e. If yes, please us how.

Please tell us about your family

Question 15 How old are the children in the household?

**Age of children**	**Please tell us the number of children in each age group**
0-4 years	
5-11 years	
12-15 years	
16-18 years	

Question 16 What area do you live in?

- Scotland
- Wales
- Northern Ireland
- East of England
- London
- Midlands
- North East and Yorkshire
- North West
- South East
- South West

Question 17 Which of the following best describes the area in which you live?

- Rural
- Urban

Questions 18 What access do you have to technology: Please tick all that apply

For peer review only

a. What type of mobile phone do you have?
    - for calls and texting only
    - Smartphone
b. What type of mobile phone contract do you have?
    - Pay as you go mobile phone
    - Monthly contract mobile phone
c. What type of computer do you have?
    - laptop
    - desktop computer
d. How much WiFi access do you have?
    - Unlimited WiFi access
    - Limited WiFi access
    - Other access to the internet, please state

Question 19 Do you have access to outdoor space? Yes/No

Question 20 We know that some children are still able to attend school. Where were the school aged children in your family when the illness or injury happened?

- All of my children were attending school
- Some of my children were attending school
- All of my children were staying at home

Question 21 What if, anything else, would you like to tell the project team?

Thank you for taking the time to complete our questionnaire.

If you would like to receive a summary of the project findings please email one of the project leads:

Professor Sarah Neill *sarah.neill@plymouth.ac.uk* or Rachel Carter *r.carter@plymouth.ac.uk*

If you have any concerns or complaints about the project please contact the Research Administrator to the Faculty Research Ethics and Integrity Committee (*hhsethics@plymouth.ac.uk*)

For peer review only

SUPPLEMENTARY FILE 3

Appendix C: Adapted Dutch version of the online survey

Bedankt voor uw hulp bij onze enquête. Wij willen graag weten wat voor gevolgen de “lockdown periode” heeft gehad op de acties en beslissingen van ouders bij de zorg of het zoeken van hulp voor een ziek of gewond kind. Onze “intelligente lockdown” ging in op 23 maart 2020 toen de overheid beperkingen oplegde om zo de verspreiding van COVID-19 te stoppen. De resultaten zullen ons helpen om te leren van de ervaringen van ouders en de ondersteuning voor ouders met een ziek of gewond kind in de toekomst te verbeteren.

Indien uw kind ziek of gewond was terwijl u het advies van de overheid opvolgde om zoveel mogelijk thuis te blijven gedurende de intelligente lockdown periode van 23 maart tot 1 juni 2020, zouden we u zeer dankbaar zijn als u onze enquête wilt invullen. We willen weten wat uw ervaringen zijn met de zorg voor een ziek of gewond kind gedurende deze periode en of u besloot om wel of niet medische hulp te zoeken. We willen graag weten of uw kind een vorm van gezondheidszorg (zoals het raadplegen van de huisarts, de huisartsenpost of ziekenhuiszorg) nodig had.

Indien u de enquête volledig invult en indient, geeft u toestemming aan ons om uw antwoorden voor ons onderzoek te gebruiken. Uw antwoorden zijn volledig anoniem en niet naar u of uw kind te herleiden. Uw antwoorden gaan rechtstreeks naar het onderzoeksteam.

De enquête bestaat uit ongeveer 20 vragen en het invullen hiervan zal maximaal 10 minuten duren.

We zullen de resultaten van het onderzoek delen in wetenschappelijke tijdschriften en websites, in communicatie met artsen en beleidsmakers, op congressen, in onderwijs en in toekomstige onderzoeksprojecten. Als u opmerkingen in de vrije tekst invult, kunnen uw woorden worden gebruikt, maar is het niet mogelijk u te identificeren. Indien u een samenvatting van onze resultaten wilt, neem dan contact op met een van de projectleiders.

Dit project is een samenwerking tussen het Erasmus MC Sophia Kinderziekenhuis in Nederland en University of Plymouth en University College London in Engeland. Deze studie is goedgekeurd door de Commissie Wetenschappelijk Onderzoek COVID-19 van het Erasmus Medisch Centrum te Rotterdam (CWO COVID-19 EMC).

De projectleiders zijn professor Henriette Moll, kinderarts Dorine Borensztajn en arts-onderzoeker Chantal Tan.

Indien u nog vragen heeft over het onderzoek, kunt u contact opnemen met de projectleiders door een mail te sturen naar *ervaringen.ouders@erasmusmc.nl*.

For peer review only

Introductie vragen over deelname aan enquête en manier van werving

1) Ik kan bevestigen dat ik bovenstaande informatie heb gelezen en ik wil graag de enquête invullen en informatie geven voor het onderzoek.

- Ja
- Nee

2) Bent u een inwoner van Nederland en was uw kind ziek of gewond kind terwijl u het advies van de overheid opvolgde om zoveel mogelijk thuis te blijven gedurende de lockdown periode?

- Ja – u wordt doorverwezen naar de vragenlijst
- Nee – we danken u voor uw interesse, maar we zijn alleen op zoek naar ouders die in Nederland wonen en wiens kind(eren) ziek of gewond was tijdens de lockdown periode.

3). Hoe bent u bij deze vragenlijst terecht gekomen?

- Ik zag een bericht met de link naar dit formulier in een Facebook groep voor ouders/moeders
- Ik zag een bericht met de link naar dit formulier via-via op Facebook
- Ik zag een bericht met de link naar dit formulier op LinkedIn
- Ik zag een bericht met de link naar dit formulier op Twitter
- Ik kreeg een bericht met de link naar dit formulier via WhatsApp toegestuurd

1. Vragen over het zieke of gewonde kind

Het kan zijn dat uw kind meerdere ziekte episodes of verwondingen heeft gehad tijdens de "lockdown periode". We willen graag dat u de meest ernstige hiervan selecteert en de volgende vragen met die ziekte of verwonding in gedachte beantwoordt.

a). Hoe oud was uw kind ten tijde van de ziekte of verwonding?

- Jonger dan 12 maanden oud
- 12 maanden of ouders maar jonger dan 24 maanden oud
- 2 jaar of ouders maar jonger dan 5 jaar oud
- 5 jaar of ouder maar jonger dan 12 jaar oud
- 12 jaar of ouders maar jonger dan 16 jaar oud
- 16 of 17 jaar oud

b). Is uw kind een jongen of een meisje?

- Meisje
- Jongen

c). Is uw kind bekend met reeds bestaande ziekten, zoals chronische of langdurige ziekten, complexe behoeften of terugkerende ziekten?

Indien ja, graag toelichting welke ziekte(n) dit zijn:

For peer review only

2. Welke kenmerken of symptomen van ziekte of verwonding had uw kind.
Vink alles aan wat van toepassing is

- Huid en uiterlijk
  - Huid erg bleek of blauw, of binnenkant van de lippen en tong blauw
  - Vlekkerige huid
  - Uitslag die verdween wanneer erop werd gedrukt
  - Uitslag die niet verdween wanneer erop werd gedrukt
- Ademhalingsproblemen
  - Ademhaling stopte voor een lange periode (>10 seconden per keer)
  - Ademhaling onderbroken (ademstops) voor 5-10 seconden
  - Moeite hebben met ademen
  - Intrekkingen in de hals, en/of onder of tussen de ribben bij elke ademhaling
  - Kreunen bij de ademhaling
  - Sneller ademen dan normaal
  - Hoorbare ademhaling (gierende of piepende ademhaling)
- Lichaamstemperatuur
  - Jonger dan 3 maanden oud met een temperatuur boven de 38oC
  - Tussen de 3 en 6 maanden oud met een temperatuur boven de 39oC
  - Ouder dan 6 maanden oud met een temperatuur boven de 38oC voor langer dan 5 dagen
  - Jonger dan 1 maand oud met een temperatuur onder de 36oC
- Uitdroging
  - Droge mond
  - Ingevallen ogen
  - Geen tranen
  - Suf
  - Minder plassen dan normaal
  - Meer plassen dan normaal
  - Minder drinken dan normaal
  - Meer drinken dan normaal
- Pijn
  - Buikpijn
  - Rugpijn
  - Pijnlijke testikels
  - Hoofdpijn
  - Persisterende pijn in een gewond arm of been
  - Oorpijn
  - Keelpijn

For peer review only

- Verandering in gedrag
    - Geagiteerd (sneller boos of geïrriteerd zijn)
    - Aanhoudend huilen/ontroostbaar
    - Toeval of epileptische aanval
    - Verward
    - Niet wakker worden of niet wakker kunnen blijven
    - Niet reagerend
- Verwonding
    - Verbranding
    - Hoofdverwonding
    - Verwonding aan been of arm
    - Val
    - Snijwond
    - Bloeding
    - Mank lopen
    - Moeite om een lichaamsdeel te bewegen
- Andere ziekte of verwonding

Mocht de ziekte en/of verwonding van uw kind niet hierboven vermeld staan, graag hieronder uw toelichting

3. Wat zou uw gebruikelijke reactie zijn op deze ziekte of verwonding zijn geweest vóór de intelligente lockdown periode?

- Om medische hulp vragen → ga naar vraag 4
- Thuis voor mijn kind zorgen → ga naar vraag 5
- Anders → ga naar vraag 5

4. Waar zou u normaliter allereerst om medische hulp hebben gevraagd voor uw kind bij deze ziekte of verwonding?

- Eigen waarnemend huisarts (overdag)
- Telefonisch consult met de huisarts
- Inloop spreekuur bij de huisarts
- Website eigen huisarts
- Website thuisarts.nl
- Huisartsenpost (in de avond/nacht)
- Behandelend kinderarts, waar mijn kind onder controle is
- 112 bellen
- Spoedeisende hulp
- Anders

For peer review only

5. Heeft u om medische hulp gevraagd voor de ziekte of verwonding van uw kind tijdens de lockdown?

- Ja → ga naar vraag 6
- Nee → ga naar vraag 9

6. Waar heeft u om medische hulp gevraagd voor deze ziekte of verwonding tijdens de lockdown?

Vink alles aan wat van toepassing is

- Eigen waarnemend huisarts (overdag)
- Telefonisch consult met de huisarts
- Inloop spreekuur bij de huisarts
- Website eigen huisarts
- Website thuisarts.nl
- Huisartsenpost (in de avond/nacht)
- Behandelend kinderarts, waar mijn kind onder controle is
- 112 bellen
- Spoedeisende hulp
- Anders

7. Was uw kind opgenomen in het ziekenhuis vanwege de ziekte of verwonding tijdens de lockdown?

8. Wat zou u nog willen vertellen over uw ervaring? Bijvoorbeeld of het advies wat u heeft gekregen nuttig was of niet. → ga naar vraag 10

9. Waarom had u besloten om geen medische hulp te vragen? Vink alles aan wat van toepassing is

- Ik wist niet zeker of mijn kind ziek of gewond genoeg was om medische hulp te zoeken
- Ik was bezorgd dat ikzelf, mijn kind of een familielid COVID-19 zou krijgen
- Ik was bezorgd dat ik kritiek zou krijgen als ik medische zorg zou gebruiken als het geen noodgeval was
- Ik dacht dat het advies om zoveel mogelijk thuis te blijven tijdens de lockdown periode betekende dat ik niet naar een gezondheidsinstelling of ziekenhuis kon gaan
- Ik maakte me zorgen over het gebruik van medische zorg wanneer deze voor andere mensen meer nodig was
- Ik was bang dat de gezondheidsinstelling (huisarts of ziekenhuis) extreem druk zou zijn en dat ik lang zou moeten wachten
- Ik heb geen auto en ik wilde geen gebruik maken van het openbaar vervoer
- Ik heb niemand die op mijn andere kinderen kan passen

For peer review only

- Anders

10. Wat heeft u nog meer gedaan aan de ziekte of verwonding van uw kind?

- Ik heb gewacht om aan te kijken of mijn kind al dan niet beter werd
- Ik heb de ziekte of verwonding van mijn kind zelf behandeld. Vink alles aan wat van toepassing is
    - Paracetamol
    - Ibuprofen
    - Een voorgeschreven puffer
    - Vicks
    - Een koude kompres/koeling met ijs
    - Schoonmaken van de wond
    - Verbinden
    - Een draagdoek om een gewond arm te laten rusten
    - Een gewond ledemaat ondersteunen door het hoog te houden
    - Koude dranken
    - Warme dranken
    - Koud bad
    - Warm bad
    - Traktaties en aandacht
    - Mijn kind afleiden
    - Huismiddeltjes, graag uw toelichting
    - Anders
- Ik heb informatie gezocht over hoe ik thuis de ziekte of verwonding kon behandelen**.** Kunt u toelichten waar of via wie u deze informatie heeft opgezocht?
    - Familie
    - Vrienden
    - Een vriend of familielid die zorgmedewerker is
    - Boeken
    - TV
    - Tijdschriften
    - Kranten
    - Radio
    - Het internet
    - App: moet ik naar de dokter?
    - Andere apps
    - Sociale media (zoals Twitter, Facebook, Instagram, Youtube)
    - Google
    - Thuisarts.nl
    - Een andere website

For peer review only

- Anders
- Ik heb informatie gebruikt welke ik al had. Kunt u toelichten waarvan of van wie u deze informatie al had gekregen?
    - Familie
    - Vrienden
    - Een vriend of familielid die zorgmedewerker is
    - Boeken
    - TV
    - Tijdschriften
    - Kranten
    - Radio
    - Het internet
    - App: moet ik naar de dokter?
    - Andere apps
    - Sociale media (zoals Twitter, Facebook, Instagram, Youtube)
    - Google
    - Thuisarts.nl
    - Een andere website
    - Anders
    -
- Niet van toepassing

11. Als de gevonden informatie nuttig was om u te helpen met de ziekte of verwonding van uw kind, wat vond u hier nuttig aan en waarom?
Als de gevonden informatie niet nuttig was, wat vond u hier niet nuttig aan en waarom?

12. Welke van de volgende bronnen voor advies bent u tegen gekomen wanneer u medische zorg nodig had gedurende de lockdown. Vink alles aan wat van toepassing is.

- Overheidsbronnen (dagelijks nieuws van overheid/RIVM)
- Gezondheidszorg bronnen (zoals website van uw eigen huisarts, thuisarts.nl, app moet ik naar de dokter?)
- Andere online expert bronnen (zoals universitaire of onderzoeksorganisaties etc)
- Deskundigen op sociale media (zoals Twitter, Facebook)
- Familie/vrienden op sociale media
- Anders

Heeft u advies gevonden in deze bron(nen) over wanneer u…

- ... de huisartsenpost moest bellen?
- … uw eigen huisarts moet bellen?

For peer review only

- … naar de spoedeisende hulp moet komen met uw kind?

Wat was nieuw aan het advies wat u heeft gezien omtrent het gebruik van medische zorg gedurende de lockdown?

13. Hebben veranderingen in de medische zorg gedurende de lockdown invloed gehad op hoe ziek uw kind was?

- Ja → graag uw toelichting
- Nee

14. Hebben veranderingen in medische zorg gedurende de lockdown invloed gehad op de behandeling die uw kind heeft gekregen?

- Ja → graag uw toelichting
- Nee
- Misschien
- Niet van toepassing

15. Hoe oud waren de kinderen in uw huishouden gedurende de lockdown periode? Vul het aantal kinderen per leeftijdsgroep in

- Leeftijd van de kinderen 0-4 jaar
- Leeftijd van de kinderen 5-11 jaar
- Leeftijd van de kinderen 12-15 jaar
- Leeftijd van de kinderen 16-17 jaar

16. In welke provincie woont u?

- Drenthe
- Flevoland
- Friesland
- Gelderland
- Groningen
- Limburg
- Noord-Brabant
- Noord-Holland
- Overijssel
- Utrecht
- Zeeland
- Zuid-Holland

Welke van de volgende opties omschrijft uw woonomgeving het beste?

For peer review only

- Grote stad
- Kleine stad/dorp
- Platteland

Had u de mogelijkheid om tijdens de lockdown buiten in uw tuin of op uw balkon te zijn?

- Ja
- Nee

17. Welke toegang heeft u tot technologie?

Wat voor mobiele telefoon heeft u ?

- Prepaid mobiele telefoon
- Maand abonnement
- Niet van toepassing

Wat voor soort computer heeft u?

- Een laptop
- Computer
- Ik heb geen computer

In welke mate heeft u toegang tot WiFi?

- Onbeperkte WiFi toegang
- Beperkte WiFi toegang
- Geen WiFi toegang
- Anders

18. We weten dan sommige kinderen nog naar school konden gaan. Waar waren de schoolgaande kinderen in uw gezin wanneer ze ziek of gewond waren gedurende de lockdown?

- Al mijn kinderen gingen naar school
- Sommige van mijn kinderen gingen naar school
- Al mijn kinderen bleven thuis

19. Heeft u nog opmerkingen voor het onderzoeksteam?

Hartelijk dank voor uw tijd en interesse om deze vragenlijst te beantwoorden.

For peer review only

Indien u een samenvatting van de bevindingen van het onderzoek zou willen ontvangen, stuur dan een mail naar de projectleider: Professor Henriette Moll (*ervaringen.ouders@erasmusmc.nl*)

Indien u enige zorgen of klachten heeft over het onderzoek, neem dan graag contact op met een onafhankelijk kinderarts (*secretariaatalkg@erasmusmc.nl*)

For peer review only

SUPPLEMENTARY FILE 4

Appendix D: Introduction of the survey on social media

Advertising text:

De coronaperiode is een zware dobber voor ouders. Is uw kind ziek of gewond geweest in de lockdown periode en wilt u bijdragen aan onderzoek van het Sophia Kinderziekenhuis, vul dan deze online enquête anoniem in (maximale duur 10 minuten):

h*ttps://forms.gle/GNyiP7G2xKnoSR9j8*

Alvast bedankt voor uw medewerking. Delen van dit bericht wordt zeer gewaardeerd!

Translation of advertising text:

The corona period is a tough one for parents. If your child has been ill or injured during the lockdown period and you want to contribute to research of the Sophia Children's Hospital, please complete this online survey anonymously (maximum duration 10 minutes):

https://forms.gle /GNyiP7G2xKnoSR9j8

Thank you in advance for your cooperation. Sharing this post is much appreciated!

Advertising image:

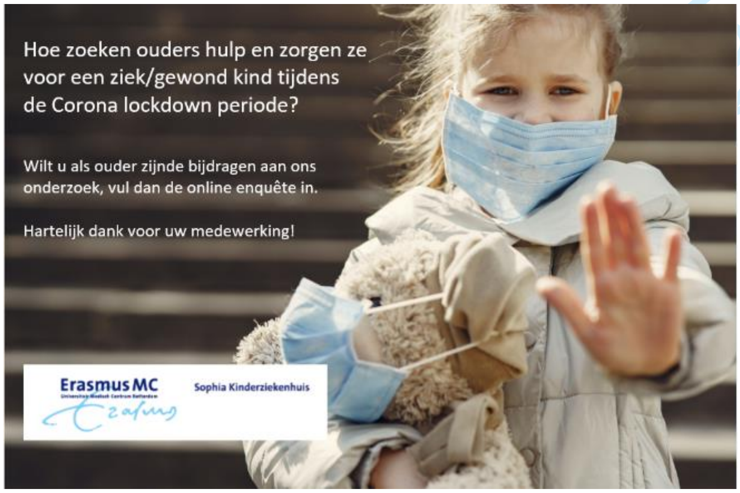

*The image was obtained from the following webpage and was freely available to use: https://www.pexels.com/photo/serious-girl-in-protective-mask-holding-plush-toy-in-mask-and-showing-palm-against-steps-4000605/

Translation of text on advertising image:

How do parents seek help and care for a sick or injured child during the corona lockdown period? Do you as a parent want to contribute to research, please fill in this online survey. Thank you very much for your cooperation!

For peer review only

SUPPLEMENTARY FILE 5

Appendix E: Table for McNemar's test

		**Did seek help during lockdown**		
**Would have sought help before lockdown**		*Yes*	*No*	*Total children*
	Yes	*83*	*4*	*87*
	No	*9*	*9*	*18*
	Total children	*92*	*13*	*105*

McNemar's test p=0.2

For peer review only

SUPPLEMENTARY FILE 6

Appendix F: Parents' help seeking behaviour related to children's characteristics

	**Sick/injured children (N=105)**	**Would have sought help before lockdown ¥**	**Did seek help during lockdown ¥**
**Age** (years)			
<5	41 (39)	37 (90)	39 (95)
5-11	43 (41)	33 (76)	36 (84)
12-17	21(20)	17(81)	17 (81)
**Gender**			
Boys	61 (58)	49 (80)	52 (85)
Girls	44 (42)	38 (86)	40 (91)
**Presenting symptom***			
Skin	22 (21)	18 (82)	21 (95)
Breathing	25 (24)	21 (84)	24 (96)
Body temperature	16 (15)	16 (100)	16 (100)
Dehydration	21 (20)	18 (86)	20 (95)
Pain	45 (45)	40 (89)	44 (94)
Change of behaviour	42 (40)	32 (76)	38 (91)
Injury	23 (22)	19(83)	23 (100)
Other	19 (18)	17 (89)	15 (75)
**Hospital admission**	37 (35)	36 (97)	37 (100)

Absolute numbers and percentages (%) are shown

* Possible to have more than 1 presenting symptom

¥ Percentage of children in that specific variable group

For peer review only

SUPPLEMENTARY FILE 6

Appendix G: Selection of quotes from parents

Severity of illness – quotes based on 32 open text answers

Positive experiences	Negative experiences
**Health care services**	
"Normally the waiting time at the emergency department can take hours. However, now we were seen and treated within 30 minutes. This spared my child hours of pain !!! Thus a very positive experience!!!!"	"My son had to undergo surgery in a tertiary hospital. Normal waiting time would be 2 weeks, but now it was extended to 1.5 month. During these times, he cried a lot and was in a lot of pain; pure hell."
"She underwent surgery on her heart defect during the lockdown."	"The doctor couldn't see my son, so the severity of the wound wasn't clear. The advice "to call back in a few days" wasn't of much help…"
	"Because my child was not seen at the GP out of office for his double ear infection, he received his antibiotic treatment later."
**Parents' behaviour**	
	"We waited a day / night too long to seek medical help out of fear."
**Reported health care professionals' behaviour**	
"The emergency department was empty and we were nearly the only ones at the paediatric ward. The doctors had a lot of time for us."	"They wanted the patients to leave as soon as possible."

Comparable quotes are not separately described

Treatment – quotes based on 39 open text answers

Positive experiences	Negative experiences
**Health care services**	
" All elective surgeries were postponed and the paediatric ward has never been this empty. We received the best medical care during this period. There was enough time to properly start palliative care."	"My son needed to wait much longer on his surgery than if there had not have been a lockdown."
	"My child did not undergo surgery but received a higher dose of antibiotics."
**Parents' behaviour**	
	"We waited longer to consult the GP. Normally we would have contacted the GP earlier, but we did not out of fear of getting infected with COVID-19 and the impact of the protective clothing health care workers wore on our child."

**Reported health care professionals' behaviour**	
	" We have spent hours in the emergency department in an isolated room without any communication. The nurses gave us little attention and we were asked to take care of the nightshift ourselves because it was difficult for the nurses to change into protective clothing. Reason for us to go home the next day."
	"The doctor could not see us for a consultation. I felt unheard and not understood."
	"Our GP was very worried, leading to daily consultations while it all started with a simple fever."

Comparable quotes are not separately described

For peer review only

STROBE 2007 (v4) checklist of items to be included in reports of observational studies in epidemiology*
Checklist for cohort, case-control, and cross-sectional studies (combined)

Section/Topic	Item #	Recommendation	Reported on page #
**Title and abstract**	1	(*a*) Indicate the study's design with a commonly used term in the title or the abstract	The study design is stated in the title and abstract. Page 1-2
		(*b*) Provide in the abstract an informative and balanced summary of what was done and what was found	The abstract is written according to the *BMJ Open* guidelines with all required information. Page 1-2
**Introduction**			
Background/rationale	2	Explain the scientific background and rationale for the investigation being reported	The background and rationale are described in the first three paragraphs of the introduction. Page 4
Objectives	3	State specific objectives, including any pre-specified hypotheses	The objective is stated in the last paragraph of the introduction. Page 5
**Methods**			
Study design	4	Present key elements of study design early in the paper	Key elements of the study design are presented in the first paragraph of the methods section, and in detail explained in the consequent

For peer review only

			paragraphs. Page 5
Setting	5	Describe the setting, locations, and relevant dates, including periods of recruitment, exposure, follow-up, and data collection	The setting, location and relevant dates are described in the paragraphs 'Study population and setting' and 'Data collection' in the methods section. Page 5-6
Participants	6	(*a*) *Cohort study*—Give the eligibility criteria, and the sources and methods of selection of participants. Describe methods of follow-up<br>*Case-control study*—Give the eligibility criteria, and the sources and methods of case ascertainment and control selection. Give the rationale for the choice of cases and controls<br>*Cross-sectional study*—Give the eligibility criteria, and the sources and methods of selection of participants	Described in the paragraphs 'Study population and setting' and 'data collection' in the methods section. There was no follow-up. Page 5-6
		(*b*) *Cohort study*—For matched studies, give matching criteria and number of exposed and unexposed<br>*Case-control study*—For matched studies, give matching criteria and the number of controls per case	Not applicable.
Variables	7	Clearly define all outcomes, exposures, predictors, potential confounders, and effect modifiers. Give diagnostic criteria, if applicable	Described in the paragraphs 'Data collection' and 'Data analysis'. Page 6-8
Data sources/ measurement	8*	For each variable of interest, give sources of data and details of methods of assessment (measurement). Describe comparability of assessment methods if there is more than one group	Described in the paragraphs 'Data analysis'. Page 7-8
Bias	9	Describe any efforts to address potential sources of bias	The survey was disseminated via social media to reach the general population (minimise

For peer review only

			selection bias). Page 6
Study size	10	Explain how the study size was arrived at	A sample size calculation was performed described in the paragraph 'Study population and setting' in the methods section. Page 5
Quantitative variables	11	Explain how quantitative variables were handled in the analyses. If applicable, describe which groupings were chosen and why	Described in 'Data analysis'. Page 7-8
Statistical methods	12	(*a*) Describe all statistical methods, including those used to control for confounding	Described in 'Data analysis'. Page 7-8
		(*b*) Describe any methods used to examine subgroups and interactions	Described in 'Data analysis'. Page 7-8
		(*c*) Explain how missing data were addressed	Not applicable.
		(*d*) *Cohort study*—If applicable, explain how loss to follow-up was addressed<br>*Case-control study*—If applicable, explain how matching of cases and controls was addressed<br>*Cross-sectional study*—If applicable, describe analytical methods taking account of sampling strategy	Not applicable.
		(*e*) Describe any sensitivity analyses	Not applicable.
**Results**			
Participants	13*	(a) Report numbers of individuals at each stage of study—eg numbers potentially eligible, examined for eligibility, confirmed eligible, included in the study, completing follow-up, and analysed	Numbers included during the first round of the survey and the second round of the survey are stated in the first paragraph of the results section. All tables and figures included the number of patients that were analysed. Page 8-10

For peer review only

		(b) Give reasons for non-participation at each stage	Not applicable.
		(c) Consider use of a flow diagram	Not applicable.
Descriptive data	14*	(a) Give characteristics of study participants (eg demographic, clinical, social) and information on exposures and potential confounders	Characteristics of the study population are described in table 1 and table 2. Page 8-10
		(b) Indicate number of participants with missing data for each variable of interest	Not applicable.
		(c) *Cohort study*—Summarise follow-up time (eg, average and total amount)	Not applicable.
Outcome data	15*	*Cohort study*—Report numbers of outcome events or summary measures over time	The numbers of the outcomes are reported in the paragraphs 'Parents' response to a sick or injured child' and 'Impact of changes in health services'. Page 10-12
		Case-control study—Report numbers in each exposure category, or summary measures of exposure	Not applicable.
		Cross-sectional study—Report numbers of outcome events or summary measures	Not applicable.
Main results	16	(*a*) Give unadjusted estimates and, if applicable, confounder-adjusted estimates and their precision (eg, 95% confidence interval). Make clear which confounders were adjusted for and why they were included	Absolute numbers and percentages are given. Chi-squared tests were used for comparing groups stratified for children with and without chronic condition. Page 8-12
		(*b*) Report category boundaries when continuous variables were categorized	Described in section 'Data analysis' in methods section.

For peer review only

			Page 7
		(*c*) If relevant, consider translating estimates of relative risk into absolute risk for a meaningful time period	Not applicable.
Other analyses	17	Report other analyses done—eg analyses of subgroups and interactions, and sensitivity analyses	Additional thematic analysis for qualitative data was performed and described in 'Data analysis' and in the last paragraph of the results section. Page 8 and page 12
**Discussion**			
Key results	18	Summarise key results with reference to study objectives	Key results are stated in the first paragraph and discussed in the subsequent paragraphs of the discussion section. Page 12-13
Limitations	19	Discuss limitations of the study, taking into account sources of potential bias or imprecision. Discuss both direction and magnitude of any potential bias	Limitations of the study are addressed in the paragraph 'Strengths and limitations' of the discussion section. Several potential biases are described. Page 13-14
Interpretation	20	Give a cautious overall interpretation of results considering objectives, limitations, multiplicity of analyses, results from similar studies, and other relevant evidence	Interpretation of the results is described in the discussion

For peer review only

			section. Page 12-14
Generalisability	21	Discuss the generalisability (external validity) of the study results	Generalizability of the results is discussed in the paragraph 'Strengths and limitations'. Page 13-14
**Other information**			
Funding	22	Give the source of funding and the role of the funders for the present study and, if applicable, for the original study on which the present article is based	Funding statement at the end of the manuscript. Page 17

*Give information separately for cases and controls in case-control studies and, if applicable, for exposed and unexposed groups in cohort and cross-sectional studies.
Note: An Explanation and Elaboration article discusses each checklist item and gives methodological background and published examples of transparent reporting. The STROBE checklist is best used in conjunction with this article (freely available on the Web sites of PLoS Medicine at http://www.plosmedicine.org/, Annals of Internal Medicine at http://www.annals.org/, and Epidemiology at http://www.epidem.com/). Information on the STROBE Initiative is available at www.strobe-statement.org.

For peer review only
